# Mechanical Overloading‐Induced Nanomineral Crystal Perturbation from the Osteochondral Interface: A Potential Initiator of Osteoarthritis

**DOI:** 10.1002/advs.202516893

**Published:** 2026-01-26

**Authors:** Nan Jiang, Rong Ren, Zhan Su, Jiahao Zhou, Pinyin Cao, Sicheng Zhang, Zhen Li, Chao Li, Songsong Zhu

**Affiliations:** ^1^ State Key Laboratory of Oral Diseases and National Center for Stomatology & National Clinical Research Center for Oral Diseases West China Hospital of Stomatology Sichuan University Chengdu Sichuan P. R. China; ^2^ Department of Orthognathic and TMJ Surgery West China Hospital of Stomatology Sichuan University Chengdu Sichuan P. R. China; ^3^ Department of Head and Neck Surgery Sichuan Clinical Research Center for Cancer Sichuan Cancer Center Sichuan Cancer Hospital & Institute Affiliated Cancer Hospital of University of Electronic Science and Technology of China Chengdu P. R. China; ^4^ AO Research Institute Davos Davos Switzerland

**Keywords:** cartilage pathological mineralization, nano mineral crystals, osteoarthritis, osteochondral interface

## Abstract

As a common degenerative joint disease, osteoarthritis (OA) has been thoroughly studied for its pathogenesis, including cell phenotype and histological morphology changes. Yet, the nano‐micro scale spatiotemporal pathological progression, especially its original site and actuating events during OA progression remain largely unknown. Here, we first proposed that interface‐derived nanocrystal fragmentation and ectopic deposition subjected to mechanical overloading may be a potential initiator for OA progression. Ectopic deposition of nanocrystals results in mechanical stiffness of osteochondral interface extracellular matrix (ECM), which subsequently upregulates fibronectin and vitronectin expression from hypertrophic chondrocytes. Hyper‐mineralized vesicles, secreted by hypertrophic chondrocytes, facilitates the ECM bottom‐up mineralization and top‐down degeneration. Furthermore, mechanical overloading removal can block the ECM mineralization and degradation, and effectively reverse the OA pathology. The present work advances our comprehension of the underlying mechanisms of mechanical overloading induced OA progression and offers a foundation for potential OA therapeutic strategies.

## Graphical abstract

Laser‐induced graphene (LIG) provides a scalable, laser‐direct‐written route to porous graphene architecture with tunable chemistry and defect density. Through heterojunction engineering, catalytic functionalization, and intrinsic self‐heating, LIG achieves highly sensitive and selective detection of NOX, NH3, H2, and humidity, supporting next‐generation wearable and standalone gas‐sensing systems.

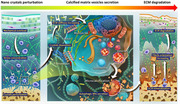

## 1 Introduction

Osteoarthritis (OA) is one of the leading causes of physical disability. This pathological condition is characterized by progressive degradation of the articular cartilage, remodeling of the subchondral bone (SB), and consequent articular dysfunction [1, 2, 3]. OA has been associated with chronic pain, structural deformities, and potentially increased all‐cause mortality [4]. Recent reports showed that the global age standardized prevalence of knee OA and hip OA were 3.8 and 0.85%, respectively [4, 5, 6]. The healthcare expenditures of OA have exceeded $46.17 billion and approximately $60.7 billion in lost productivity costs per year [4]. While significant progress has been made, the complete pathophysiological mechanism of OA remains incompletely understood. OA patients typically undergo joint replacement surgery at the end stage of the disease after experiencing joint pain and stiffness for decades.

OA is a complex, heterogeneous disease that is initiated by multiple factors and also involves multiple pathological mechanisms including genetic, molecular, cellular, metabolic, inflammatory, mechanical. Current studies on OA mechanism and progression primarily focuses on the macroscopic phenotypes, histological analysis, and biomechanical related aspects [7, 8, 9, 10]. From the histological perspective, OA is described as collagen fibrillation, chondrocyte aggregation in early stage, and extracellular matrix (ECM) degradation as well as osteophyte formation in advanced stage [11, 12]. Concomitantly, the mechanical properties of cartilage undergo alteration following the onset of OA [13, 14, 15]. Previous studies have demonstrated that the macroscopic mechanical properties of OA cartilage undergo a significant decline [16, 17]. However, the progression of phenotypes at the micro and nanoscales, as well as the spatial‐temporal characteristics of cartilage mechanical properties, remain largely unknown. This knowledge gap may limit our current understanding of OA etiology and pathogenesis, potentially affecting therapeutic target identification.

Investigation of OA initiation site represents a significant challenge in the field of phenotypic analysis [7]. Previous studies have indicated that OA originates from the articular cartilage surface [7, 18]. Early stage of OA are characterized by ECM fibrillation and surface erosion, which progressed by a top‐down pattern [7]. However, another research has proposed an alternative hypothesis, namely that OA commences from the SB [19, 20]. The findings indicated that mechanical overloading could result in gradual pathological bone remodeling in OA early stage, ultimately leading to the formation of denser SB plates in advanced stage [11, 19]. Both early bone loss and late osteosclerosis will serve to further exacerbate the mechanical overloading of the overlying cartilage, thereby triggering ECM continuous degeneration and OA progression [21]. Thus, the primary initiation site of OA continues to be debated in the field. Recent studies have increasingly focused on the osteochondral interface, which integrates articular cartilage and SB as a functional osteochondral unit, serving as both a mechanical pivot and a frontier of mineralization [3, 22]. However, the mechanisms governing the osteochondral interface remain incompletely understood, particularly its pivotal role in the pathogenesis of OA. The osteochondral interface is now recognized as a critical regulator of joint homeostasis, where crosstalk between cartilage and SB maintains structural and metabolic equilibrium [3]. In OA, disruption of this interface marked by aberrant mineralization, vascular invasion, and inflammatory signaling triggers a vicious cycle of cartilage degradation and SB remodeling. This pathological interplay accelerates joint dysfunction, highlighting the interface as a key therapeutic target for early OA intervention. While multiple pathways can initiate OA, this study focuses specifically on mechanical overloading induced osteochondral interface disruption as one potential triggering mechanism. Deciphering the molecular and biomechanical mechanisms of the osteochondral interface is thus essential.

Pathological cartilage mineralization appears to be an important pathogenic feature of osteochondral interface, usually resulting in disruption of interface mechanics and structural homeostasis and the onset and progression of OA [23, 24, 25]. Recent studies showed that cartilage mineralization, identified in almost all osteoarthritic joints of patients, strongly correlates with the severity of OA [26, 27]. It has been demonstrated that pathological cartilage mineralization may be mediated by the hypertrophic differentiation of OA chondrocytes. The release of calcified matrix vesicles (MVs) provides a source of minerals for cartilage mineralization [28, 29]. Other studies have indicated that pathological cartilage mineralization may serve as a primary target for the combined effects of mechanical stimulus, cartilage degradation, and SB remodeling [7]. Conversely, the inhibition of pathological mineralization has been demonstrated to impede the progression of OA [26]. These observations suggest that pathological mineralization may contribute to OA progression beyond being a secondary consequence [23]. Exploring the mechanisms of pathological mineralization is crucial in underlying mechanisms of OA progression and developing new therapeutic targets [23].

Mechanical overloading has been identified as an important contributing factor to pathological mineralization in OA [13, 16]. Lots of clinical risk factors for OA cartilage pathological mineralization are pointed to cartilage mechanical overloading [30]. The most common clinical examples are irreparable anterior disk displacement (ADD) of the temporomandibular joint (TMJ) and destabilization of the medial meniscus (DMM) in knee joint. Additionally, mechanical overloading of the knee has also been observed in cases of obesity‐induced osteoarthritis [31, 32]. Analogous OA animal models have been constructed by a variety of mechanical destabilization methods [33, 34]. Clinical evidence also demonstrates that the reduction of displaced disk or meniscus can significantly relieve the mechanical stress on articular cartilage and contribute to the reversal of cartilage phenotype [35, 36]. Nevertheless, the micropathological mechanism underlying these phenomena remains unclear and urgently needs to be elucidated.

The TMJ demonstrates high susceptibility to OA development, with some studies suggesting comparable or greater susceptibility than the knee joint in certain populations, and its biomechanical interplay with dental occlusion is well‐documented [37, 38]. A key driver of OA pathogenesis in the TMJ is mechanical overloading of the cartilage, often resulting from ADD. To systematically investigate OA mechanisms triggered by mechanical stress, we established a rabbit model of ADD‐induced TMJOA as our primary platform, supplemented by murine and rat models of TMJOA and knee OA for comparative analysis. We further validated key findings using human and porcine TMJOA samples. Leveraging these integrated models, our study aims to: (i) construct a high‐resolution spatiotemporal atlas of OA cartilage; (ii) delineate the role of dysregulated osteochondral nanomineral crystals in disease progression; and (iii) decode the mechanisms driving pathological cartilage mineralization (Figure S1). Although OA is a whole‐joint disorder, its initial triggers and progression are often joint‐specific. Our strategy utilizes the TMJ, a fibrocartilaginous joint with well‐defined loading conditions, as a discovery platform for high‐resolution mechanistic insights, while confirming the broader relevance of our findings in knee OA models [39]. This approach may unveil key physicochemical processes in mechanically driven OA and identify new therapeutic targets.

## 2 Results

### 2.1 Nano‐Micro Structure and Composition Characteristics of Cartilage During OA Development

**FIGURE 1:**
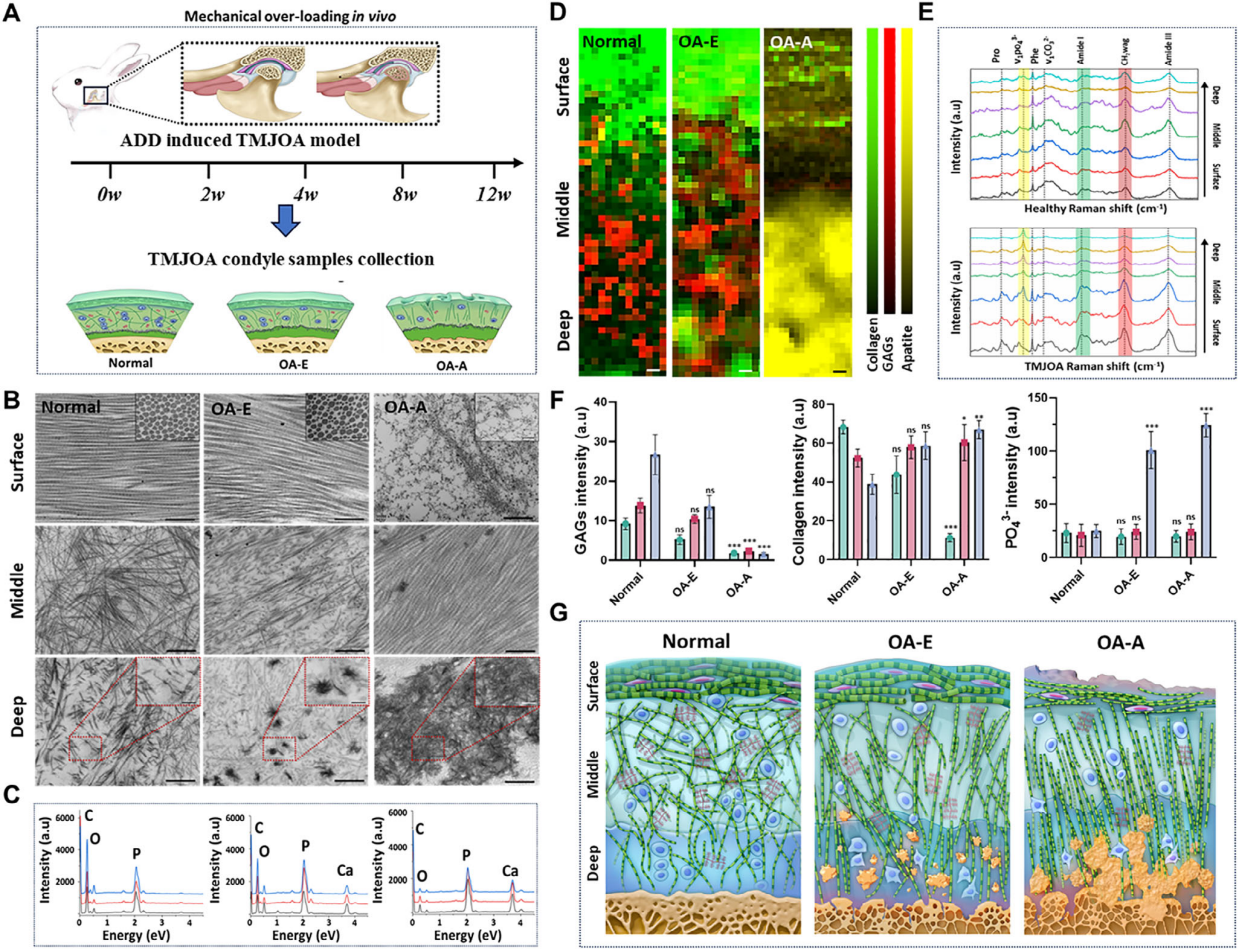
Nano‐micro structure and composition characteristics of cartilage during TMJOA development. (A) Schematic diagram of mechanical overloading induced TMJOA. (B) TEM images of the rabbit TMJOA cartilage from normal to OA‐A stages. Scale bar, 200 nm. High magnification of the area in surface layer showed cross section of collagen fibrils. Scale bar, 100 nm. High magnification of the area depicted by the red rectangles showed calcified particulates and mineralized deposits. Scale bar, 100 nm. (C) Elemental analysis of the nano mineral crystals indicated by the deep layer in (B). (D) Collected Raman maps of different stages cartilage showing minerals (960 cm^−1^), collagen (1247 cm^−1^) and GAGs contents (1490 cm^−1^). Scale bar, 10 µm. (E) Raman spectra of the rabbit TMJOA cartilage tissues from surface to deep layer of normal and OA samples. (F) Quantitative analysis of collagen, GAGs and PO_4_^3−^ distribution in the cartilage. (*n* = 3). Results are presented as means ± SD, ns, no significance. *p < 0.05, **p < 0.01, and ***p < 0.001; statistical significance was calculated using one‐way analysis of variance (ANOVA) followed by Tukey's post‐hoc test for multiple comparisons, all tests were two‐sided. (G) Schematic diagram of nano‐micro structure and composition characteristics of cartilage during OA development.

OA cartilage samples were studied from four different animals and OA patients (Figure 1A). The condyles from rabbit TMJOA model induced by ADD were presented as the representative and categorized into normal, OA‐early (OA‐E), and OA‐advanced (OA‐A) stages based on osteoarthritis research society international (OARSI) scores (Figure S2). After the differences in histological features were investigated (Figures S3–S6), the nano‐micro structural and compositional changes along the depth dependent levels were identified. In normal samples, collagen fibrils near the articular surface were tightly packed with visible transverse striations, while middle and deep layers exhibited loose, random fibril arrangements around chondrocytes (Figure 1B,C and Figure S7, S8). OA‐E samples displayed sporadic needle‐like mineral crystals between deep‐layer fibrils, with surface and middle layers remaining intact. In OA‐A samples, cartilage erosion was prominent, with deep‐layer mineralization evident as spherites, calcified fibrils, and dense deposits. Surface fibrils degraded into fragments, while middle‐layer fibrils reoriented into tightly aligned bundles (Figure 1B,C and Figures S7, S8). Similar structural changes were observed in human, porcine, rat, and mouse TMJOA condyles, as well as rodent knee OA specimens (Figures S9 and S10), which validated the cross‐species and cross‐joint universality of OA cartilage nano‐micro structural changes.

Variations in the nano‐micro structure of OA cartilage prompted us to next analyze the detailed compositional changes using microscopic Raman spectrum (Figure S11). Raman spectroscopy revealed a depth‐dependent gradient in normal samples: collagen (1247 cm^−^^1^) decreased from surface to deep layers, while glycosaminoglycans (GAGs) (1490 cm^−^^1^) increased (Figure 1D–F). OA‐E samples showed phosphate mineral deposition (960 cm^−^^1^) in deep layers without surface and middle matrix degradation. In OA‐A samples, mineralization (960 cm^−^^1^) intensified, while collagen (surface) and GAGs (middle) significantly degraded (Figure 1D–F). Transmission electron microscopy‐energy dispersive X‐ray spectroscopy (TEM–EDS) confirmed Ca/P‐rich hydroxyapatite (HAP) polycrystalline deposits in deep layers, with selected area electron diffraction (SAED) verifying crystallinity (Figure 1C and Figure S7, S12). The Ca/P ratio indicated increasing mineral maturity with OA progression (Figure S12). A 3D schematic summarized these structural and compositional transitions (Figure 1G).

### 2.2 Spatiotemporal Differences in Loading Energy Dissipation Responded to Nano‐Micro Structural and Compositional Changes

**FIGURE 2:**
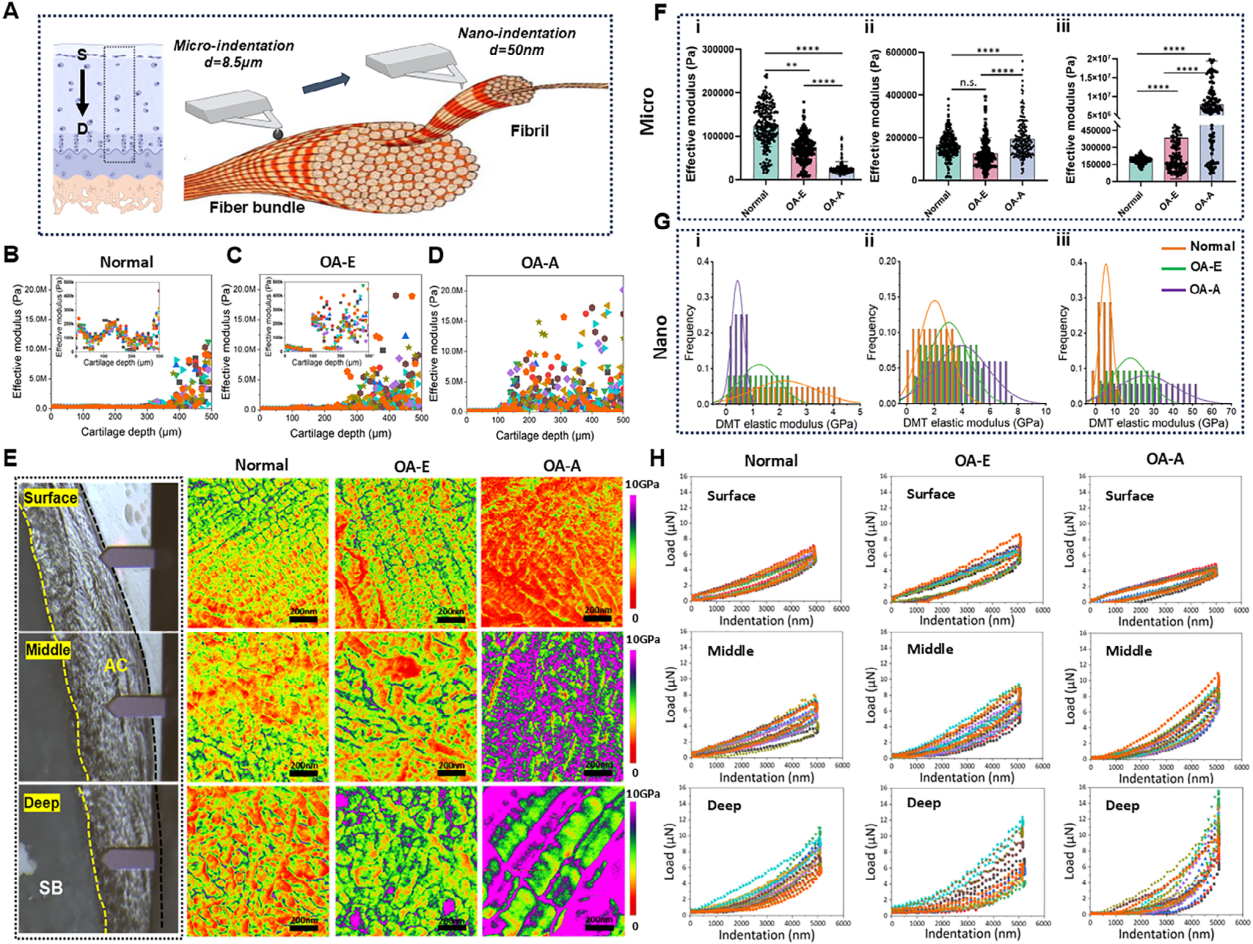
Spatiotemporal differences in loading energy dissipation responded to nano‐micro structural and compositional changes. (A) Schematic diagram showing mechanical properties of cartilage tested by nanoindentation (micro scale, tip diameter = 8.5 µm) and AFM (nano scale, tip diameter = 50 nm). (B–D) Elastic modulus distribution measured by nanoindentation (*d* = 8.5) from surface to subchondral bone in normal, OA‐E and OA‐A. (E) Consecutive AFM stiffness maps of selected regions in surface, middle and deep layer for different samples. AC, articular cartilage; SB, subchondral bone. (F) Quantitative analysis of elastic modulus in nanoindentation test (*n* = 100). Results are presented as means ± SD, ns, no significance. **p < 0.01, and ****p < 0.0001; Statistical significance was calculated using one‐way ANOVA followed by Tukey's post‐hoc test for multiple comparisons, all tests were two‐sided. (G) Corresponding stiffness distributions of AFM‐indentation in E. (H) Force displacement curve obtained from nanoindentation test to reveal loading energy dissipation (the slope represents the modulus and the area enclosed by the curve represents the energy dissipation).

Differences in nano‐micro structure and composition during OA progression may lead to different responses of mechanical properties. We then mapped the microscale stiffness of OA cartilage in a depth‐dependent direction (Figure 2A). Nanoindentation (tip radius = 8.5 µm) revealed increasing Young's modulus from surface to deep layers in normal condylar cartilage (surface: 124.32 ± 47.93 kPa; middle: 165.78 ± 60.05 kPa; deep: 189.27 ± 32.07 kPa) (Figure 2B–E). This gradient aligned with structural and compositional differences (Figure 2B–D and Figure S13). In OA‐E, the surface and middle moduli remained stable, while the deep layer modulus increased significantly (383.01 ± 35.98 kPa), consistent with early mineralization (Figure 2E). In OA‐A, deep‐layer mineralization expanded, elevating stiffness (7.99 ± 11.46 MPa), while the middle layer showed a slight increase (192.23 ± 85.17 kPa) and the surface modulus decreased (26.82 ± 14.82 kPa) due to degradation (Figure 2E and Figure S13). AFM‐based nanoindentation (tip radius = 50 nm) resolved ECM stiffness heterogeneity at the fibril level (Figure 2A,F). Normal cartilage exhibited increasing stiffness with depth (surface: 2.19 ± 1.21 GPa; middle: 2.01 ± 1.10 GPa; deep: 5.23 ± 3.02 GPa). OA‐E: deep‐layer stiffness sharply increased (17.87 ± 9.23 GPa; range: 8.64–27.10 GPa), while surface/middle layers remained near‐normal (Figure 2G and Figure S14). OA‐A: deep stiffness peaked higher (26.01 ± 15.04 GPa), middle stiffness increased (3.92 ± 1.95 GPa), and surface stiffness declined (0.42 ± 0.23 GPa). OA‐E and OA‐A samples exhibited broader stiffness distributions in deep layers compared to normal (Figure 2G and Figure S14). These changes led to increased energy dissipation in deep layers and reduced dissipation in surface layers (Figure 2H and Figure S15).

### 2.3 OA May Initiate from the Osteochondral Interface by Bottom‐Up Mineralization

**FIGURE 3:**
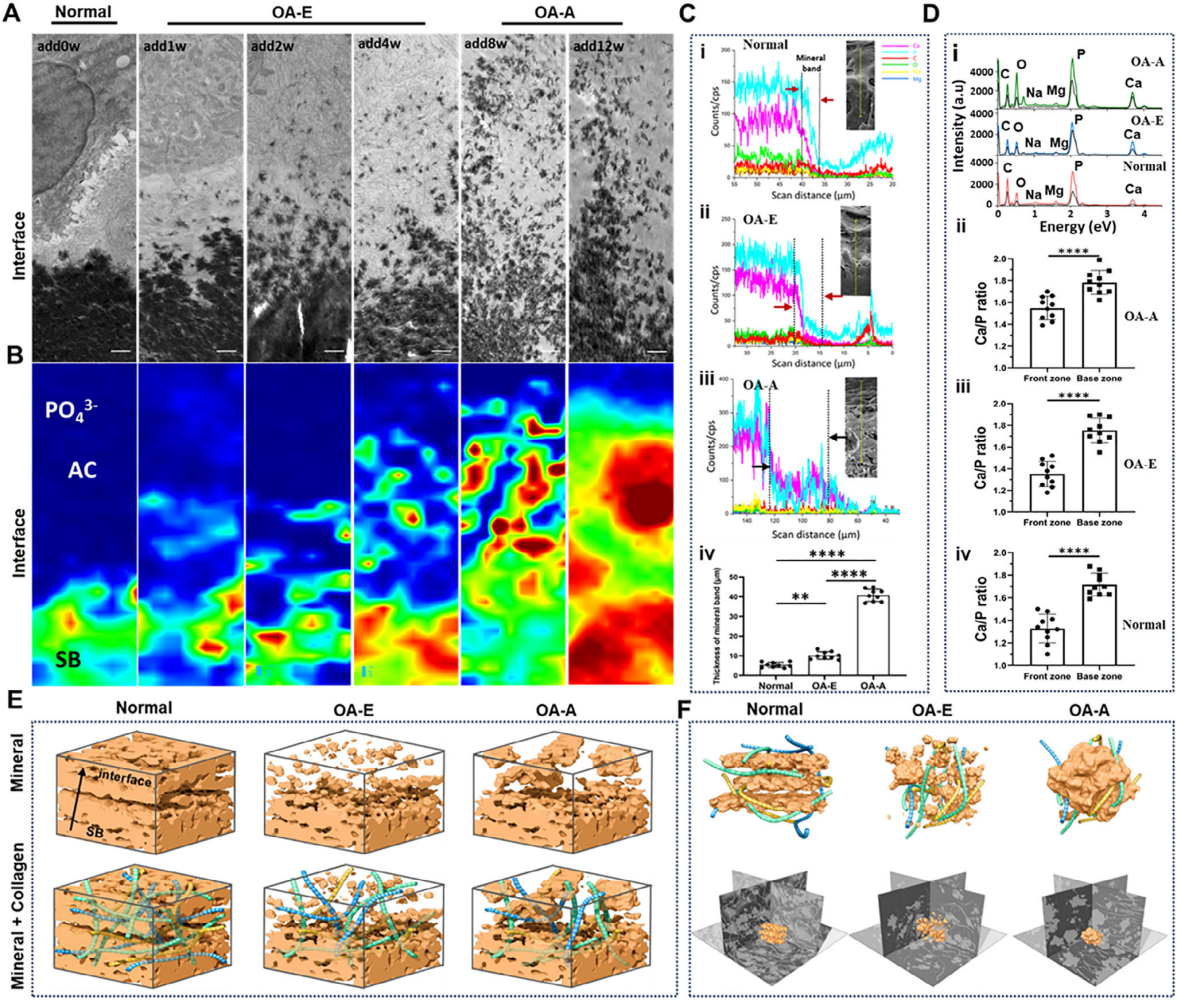
OA may initiate from the osteochondral interface by bottom‐up mineralization. (A) TEM images of osteochondral interface area in different OA stages. Mineral front and base zone were divided from AC to SB. AC, articular cartilage; SB, subchondral bone. Scale bar 2 µm. (B) Collected Raman maps showing HAP contents (960 cm^−1^) revealing the mineral crystals across the interface from normal to OA‐A. Spatial resolution was 1 µm and scale bar 5 µm. (C) The corresponding SEM–EDS line scan showing minerals distribution at osteochondral interface from mineral front zone to base zone (i–iii). Exponential increment regions were calculated as the thickness of mineral band. (iv) in (C) showed the thickness of mineral band for different samples. Results are presented as means ± SD, **p < 0.01, and ****p < 0.0001; statistical significance was calculated using one‐way ANOVA followed by Tukey's post‐hoc test for multiple comparisons, all tests were two‐sided. (D) Chemical composition of minerals in mineral front and base zone. The corresponding Ca/P ratios of minerals within each zone for different samples also calculated (ii–iv). Results are presented as means ± SD, ****p < 0.0001; statistical significance was calculated using two‐tailed Student's *t*‐test for multiple comparisons, all tests were two‐sided. (E) 3D schematic diagram of the mineralization front of osteochondral interface showing distinct mineral assembling patterns and void distribution for different samples. (F) 3D schematic diagram of the mineral spherites (orange) and the fibrils within minerals delineating the spatial relationship between mineral crystals and fibrils (upper). 3D schematic diagram of typical mineral particles intersecting at the mineralization front of interface region, demonstrating the mineral appearance and interior structures under pathological condition (lower).

Osteochondral interface is the boundary of minerals distribution and hinge of mechanical load transfer. To further elucidate the relationship between mechanical loading and mineralization patterns at the interface, we characterized the mineralization microstructures in the ADD‐induced TMJOA model. Collagen hybridizing peptide (F‐CHP) staining revealed that triple‐helix damage occurs first at the interface in OA‐E, which was induced by mechanical stress concentration at the osteochondral interface (Figure S16), as demonstrated by finite element analysis (FEA) (Figure S17). Von Kossa and Alizarin Red S staining revealed progressive calcium deposition, with OA‐E and OA‐A exhibiting initial and advanced mineralization, respectively (Figures S18–S20). Quantification confirmed significantly larger calcified areas in both OA groups versus controls. TEM and Raman mapping demonstrated structural mineralization gradients in normal samples, which manifested a narrow mineral front zone adjacent to cartilage and a highly mineralized base zone (Figure 3A,B). OA‐E disrupted this spatial organization, allowing microcracks and fragmented nanocrystals to infiltrate the cartilage matrix. Notably, in OA‐A, mineral deposition further expanded into deep cartilage, forming calcified plaques and a thickened mineral band (Figure 3A,B and Figure S20). Scanning electron microscopy–energy dispersive X‐ray spectroscopy (SEM–EDS) confirmed interface thickening in OA, supporting a bottom‐up mineralization pattern (Figure 3C). Mineral composition varied between front and base zones, with Ca/P ratios increasing toward the base (Figure 3D and Figure S21). Nanocrystal morphology analysis revealed distinct mineralization patterns. Normal samples exhibited homogeneous mineral packing with uniform voids in both zones (Figure 3E), whereas OA‐E samples displayed fragmented needle‐like crystals in the front zone and compacted materials with smaller voids in the base zone (Figure 3E,F). In OA‐A, front zone exhibited larger, irregular aggregates with increased void formation; base zone maintained dense packing but with finer, evenly distributed voids (Figure 3F). A 3D schematic summarized these nanoscale mineral structural changes during OA progression (Figure 3F).

### 2.4 Pathological Mineralization Originates from Interface‐Derived Nanocrystal Fragmentation and Ectopic Deposition

**FIGURE 4:**
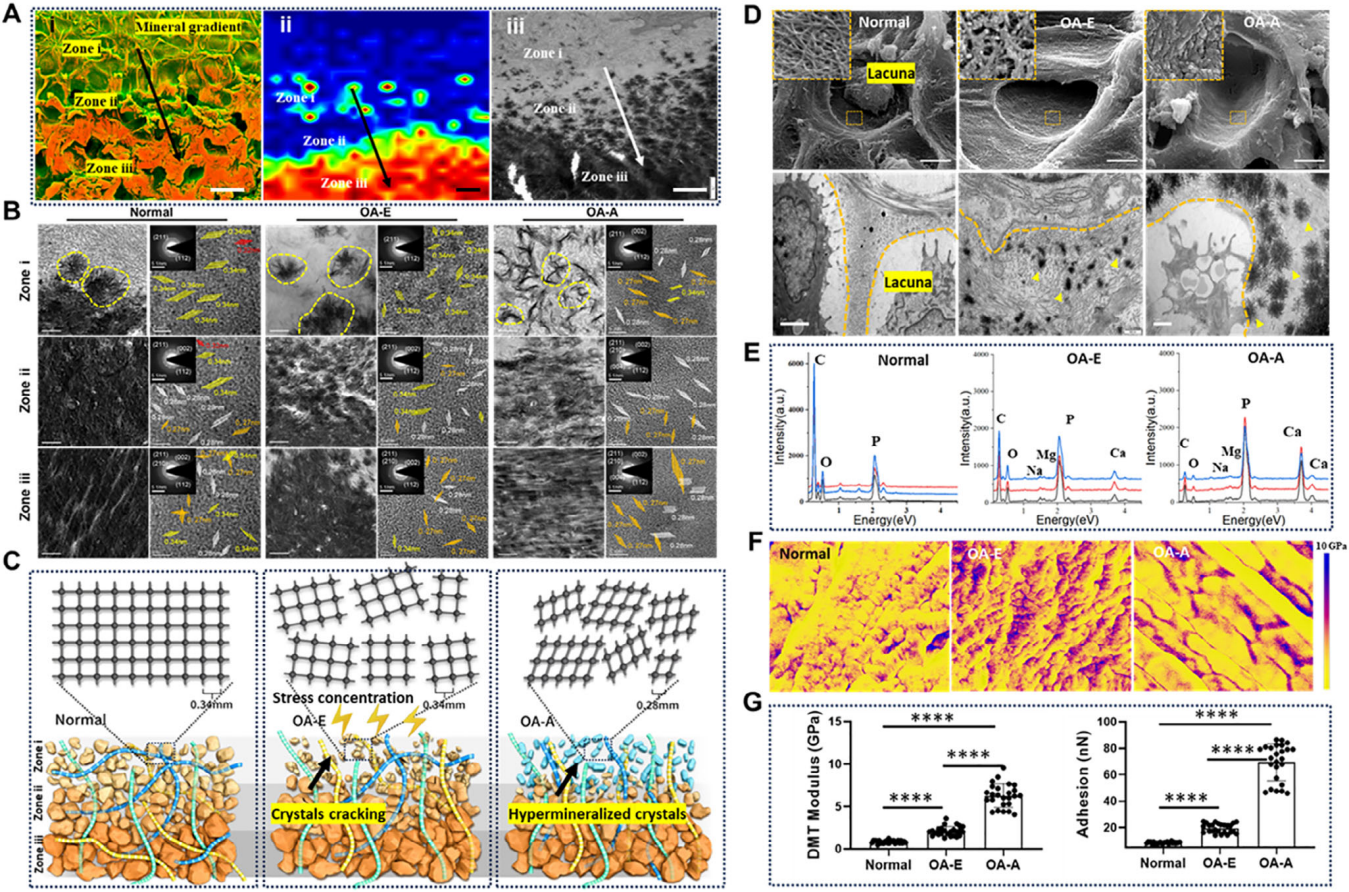
Pathological mineralization originates from interface‐derived nanocrystal fragmentation and ectopic deposition. (A) The enlarged density dependent color SEM micrographs (scale bar 50 µm), Raman mapping (scale bar 5 µm), and TEM image (scale bar 1 µm) showed the microcrystals perturbation in OA samples and divided mineral region into three micro‐areas from zone i to iii. (B) HRTEM and SAED images revealing mineral assemblies and their crystallinities at zone i–iii of osteochondral interface in different samples. Scale bar in TEM = 500 nm, in HRTEM = 5 nm, in SAED = 5 1/nm. (C) 3D schematic diagram of nanocrystals perturbation at osteochondral interface. (D) SEM and TEM images showed the mineral crystals deposited in the lacuna of hypertrophic chondrocytes in front of interface in different OA stages. Scale bar in SEM 10 µm, scale bar in TEM 2 µm, 500 nm, and 1 µm, respectively. High magnification of area depicted by the orange rectangles in SEM showed mineral particles deposited between the fibrils of lacuna. Scale bar 200 nm. (E) Elemental analysis of the mineral particulates indicated by the yellow arrow in (D). (F) AFM stiffness maps of interesting area inside of lacuna matrix in different OA stages. (G) Quantitative analysis of modulus and adhesion force in (F) (*n* = 3). Results are presented as means ± SD, ****p < 0.0001; statistical significance was calculated using one‐way ANOVA followed by Tukey's post‐hoc test for multiple comparisons, all tests were two‐sided.

Pathological mineralization in OA initiates through nanoscale crystal perturbation, progressing through three distinct microregions (zones i–iii) (Figure 4A). High‐resolution transmission electron microscopy (HRTEM)/SAED analyses revealed: (i) normal samples displayed gradually increasing crystal sizes (zone i: 3 ± 0.5 nm; zone ii: 8 ± 1.2 nm) with fused structures in zone iii; (ii) OA‐E samples contained fractured nanocrystals with 40 ± 5% size reduction (*p* = 0.003) but preserved lattice integrity (d‐spacing 0.344 ± 0.002 nm); (iii) OA‐A samples exhibited amorphous precursor clusters and hyper‐mineralized crystals showing 210 ± 15% size increase (*p* < 0.001) and aspect ratio of 2.8 ± 0.3 (Figure 4B,C). EDS quantification confirmed comparable Ca/P ratios between OA‐E (0.85 ± 0.25) and normal groups (0.47 ± 0.33) (*p* = 0.21), while OA‐A samples showed significantly elevated ratios (1.82 ± 0.04, *p* < 0.001) (Figure 4D,E and Figure S21). Raman spectroscopy confirmed carbonate‐substituted hydroxyapatite predominance, with OA‐E exhibiting higher carbonate incorporation (CO_3_^2^^−^/PO_4_^3^^−^ = 1.25 ± 0.5) and lower mineralization (mineral/matrix = 0.25 ± 0.05) compared to OA‐A advanced mineralization (mineral/matrix = 0.51 ± 0.05, *p* = 0.004) with reduced carbonate substitution (CO_3_^2^^−^/PO_4_^3^^−^ = 0.45±0.1, *p* = 0.008) (Figures S22–S24). These compositional changes correlated with mechanical property alterations, including increased pericellular matrix stiffness (Derjaguin–Muller–Toporov, DMT modulus: 2.52 ± 0.3 to 6.21 ± 0.5 GPa, *p* < 0.001) and enhanced chondrocyte adhesion (20.7 ± 1.1 to 71.5 ± 10.2 nN, *p* = 0.005) (Figure 4F,G).

### 2.5 Calcified MVs Mediate Advanced Mineralization

**FIGURE 5:**
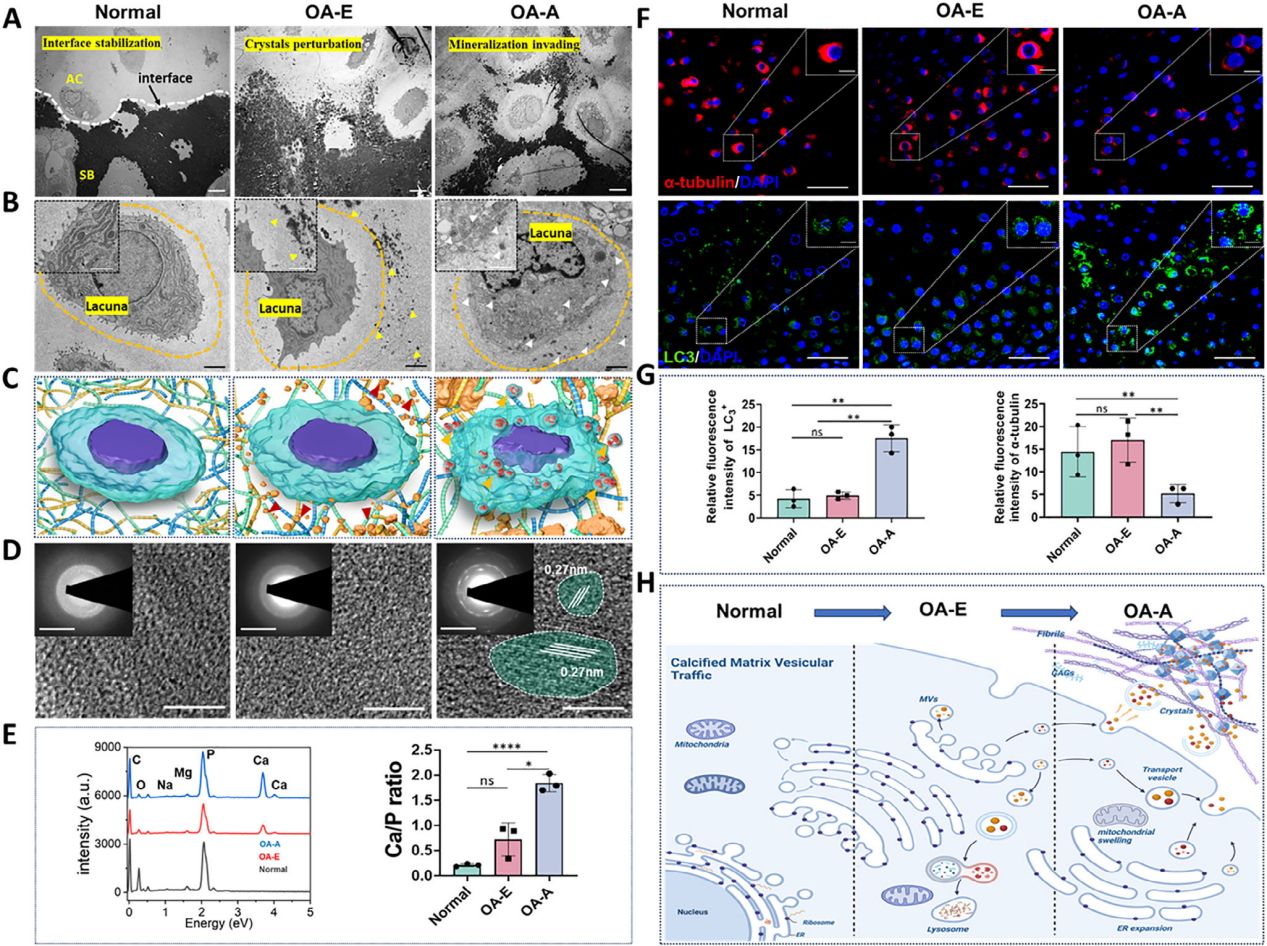
Calcified MVs mediate advanced mineralization. (A) TEM images showed the nano‐micro structure of interface and the relationship between mineral crystals and chondrocytes. Scale bar 10 µm. (B) TEM images showed the structure of hypertrophic chondrocyte in front of mineral interface. Scale bar 5 µm. Top left high magnification image showed local interesting area of chondrocytes in different OA samples. Yellow arrow indicated mineral crystals and white arrow indicated Ca‐containing MVs. Scale bar 500 nm. (C) 3D schematic diagram of hypertrophic chondrocytes structure changes during OA development. Red arrows indicated nano mineral crystals deposited surrounding of chondrocytes, and orange arrows indicated MVs formation and secretion in chondrocytes. (D) HRTEM and SAED images of the interesting area inside of chondrocytes in (B). Scale bar in HRTEM = 5 nm, in SAED = 5 1/nm. (E) Elemental analysis of the interesting area in (B) and quantitative analysis of the Ca/P ratio. Results are presented as means ± SD, ns, no significance, *p < 0.05, and ****p < 0.0001; statistical significance was calculated using one‐way ANOVA followed by Tukey's post‐hoc test for multiple comparisons, all tests were two‐sided. (F) Immunofluorescence staining of LC_3_ protein and α‐tubulin in osteochondral interface regions of different OA stages. Scale bar, 50 µm. Top right high magnification image showed local interesting area of chondrocytes in different OA samples. Scale bar 20 µm. (G) Quantitative analysis of the fluorescence intensity of LC_3_ expression and α‐tubulin in (F) (*n* = 3). Results are presented as means ± SD, ns, no significance. **p < 0.01; statistical significance was calculated using one‐way ANOVA followed by Tukey's post‐hoc test for multiple comparisons, all tests were two‐sided. (H) Schematic diagram of Ca‐containing MVs released from hypertrophic chondrocytes.

To explore the mineral origination of OA‐A, hypertrophic chondrocytes in front of osteochondral interface were further analyzed. Our findings demonstrate that calcified MVs mediate pathological mineralization in advanced OA through autophagic processes. TEM analysis revealed hypertrophic chondrocytes in OA‐A samples were enveloped by dense mineral matrices (Figure 5A–C and Figures S25, S26), with intracellular HAP nanocrystals identified by HRTEM/SAED (Figure 5D). EDS quantification showed MVs membranes contained Ca, P, and O, with Ca/P ratios increasing from 0.56 ± 0.04 in OA‐E to 1.45 ± 0.10 in OA‐A (Figure 5E and Figure S26). Immunofluorescence indicated autophagic flux disruption in OA‐A, evidenced by LC_3_^+^ upregulation (2.8‐fold, *p* < 0.01) and α‐tubulin reduction (62% decrease, *p* < 0.05) compared to normal controls (Figure 5F,G Figure S27). These findings establish a direct association between MVs‐mediated mineralization and hypertrophic chondrocyte autophagy in OA progression (Figure 5H).

### 2.6 Fibronectin and Vitronectin Bridge Early Pathological Mineralization to Advancement

**FIGURE 6:**
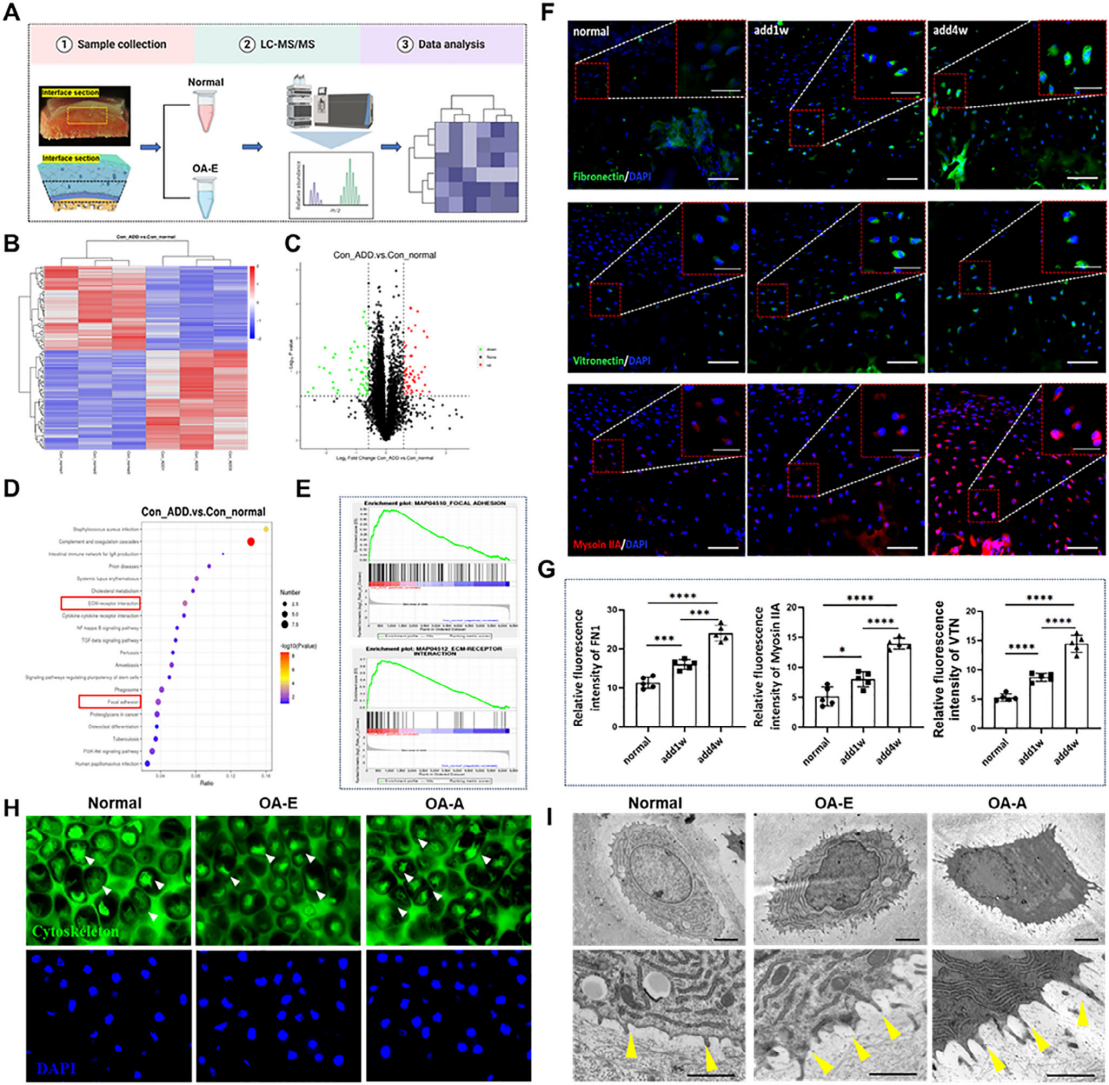
Fibronectin and vitronectin bridge early pathological mineralization to advancement. (A) Experimental procedure for the proteomic investigation. (B) Heatmap of differentially expressed proteins in osteochondral region of normal and OA‐E stages (*n* = 3). (C) Volcano plots of differentially expressed proteins in osteochondral region between normal and OA‐E stages (*n* = 3), showing that mechanical responding proteins, fibronectin, and vitronectin were upregulated. (D) KEGG pathways showing that ECM‐receptor interaction and focal adhesion pathway was enriched. (E) GSEA showing the enrichment of proteins associated with ECM‐receptor interaction and focal adhesion. (F) Immunofluorescence staining of fibronectin (green), vitronectin (green), and myosin IIA (red) in osteochondral interface tissues. Scale bar, 50 µm. Top right high magnification image showed local interesting area of chondrocytes in different OA samples. Scale bar 20 µm. (G) Quantitative analysis of the relative fluorescence intensity of fibronectin, vitronectin, and myosin IIA in (F) (*n* = 5). Results are presented as means ± SD, *p < 0.05, ***p < 0.001, and ****p < 0.0001; statistical significance was calculated using one‐way ANOVA followed by Tukey's post‐hoc test for multiple comparisons, all tests were two‐sided. (H) Cytoskeletal staining for hypertrophic chondrocyte in osteochondral interface from normal to OA‐A. White arrow indicates chondrocyte cytoskeleton. (I) TEM images showing the morphology of hypertrophic chondrocytes and their adhesion state to surrounding ECM. Yellow arrow indicates process of chondrocytes.

In OA‐E, mineral perturbation in the deep cartilage matrix resulted in a significant increasing stiffness in the ECM of hypertrophic chondrocytes. To analyze how the hypertrophic chondrocytes further promoting matrix mineralization in OA‐A, we performed proteomic analysis (Figure 6A). Proteomic analysis results revealed that 57 proteins upregulated in OA‐E cartilage, with fibronectin and vitronectin showing the most significant elevation among ECM‐interacting proteins (Figure 6B,C). KEGG pathway analysis demonstrated activation of ECM interaction and focal adhesion pathways (*p* < 0.001) (Figure 6D,E), indicating mechanosensitive responses to the mineral‐induced stiffness increase (2.52 ± 0.3 to 6.21 ± 0.5 GPa, *p* < 0.001). Immunofluorescence validation confirmed stage‐dependent upregulation of these proteins (1.2–1.5‐fold in OA‐E, 2.1–2.5‐fold in OA‐A vs normal, *p* < 0.01) (Figure 6F,G and Figure S28). In vitro experiment of human fibronectin protein cocultured with chondrocytes was performed to validated the relationship between the fibronectin and secretion of calcified matrix vesicles. The result showed that fibronectin could induce calcified nodule formation and calcified MVs secretion (Figure S29). Cytoskeletal analysis showed OA‐E chondrocytes developed elongated processes with membrane ruffling near ECM contact sites (Figure 6H,I), accompanied by microtubule disassembly (α‐tubulin reduction 62 ± 5%, *p* < 0.01) (Figure 5F and Figure S27). These structural changes correlated with autophagic vesicle formation (LC_3_^+^ vesicles increased 2.8‐fold, *p* = 0.003), establishing a mechanotransduction pathway linking matrix mineralization to cellular mineralization responses.

### 2.7 Mechanical Overloading Drives Pathological Mineralization via Fibronectin Upregulation

**FIGURE 7:**
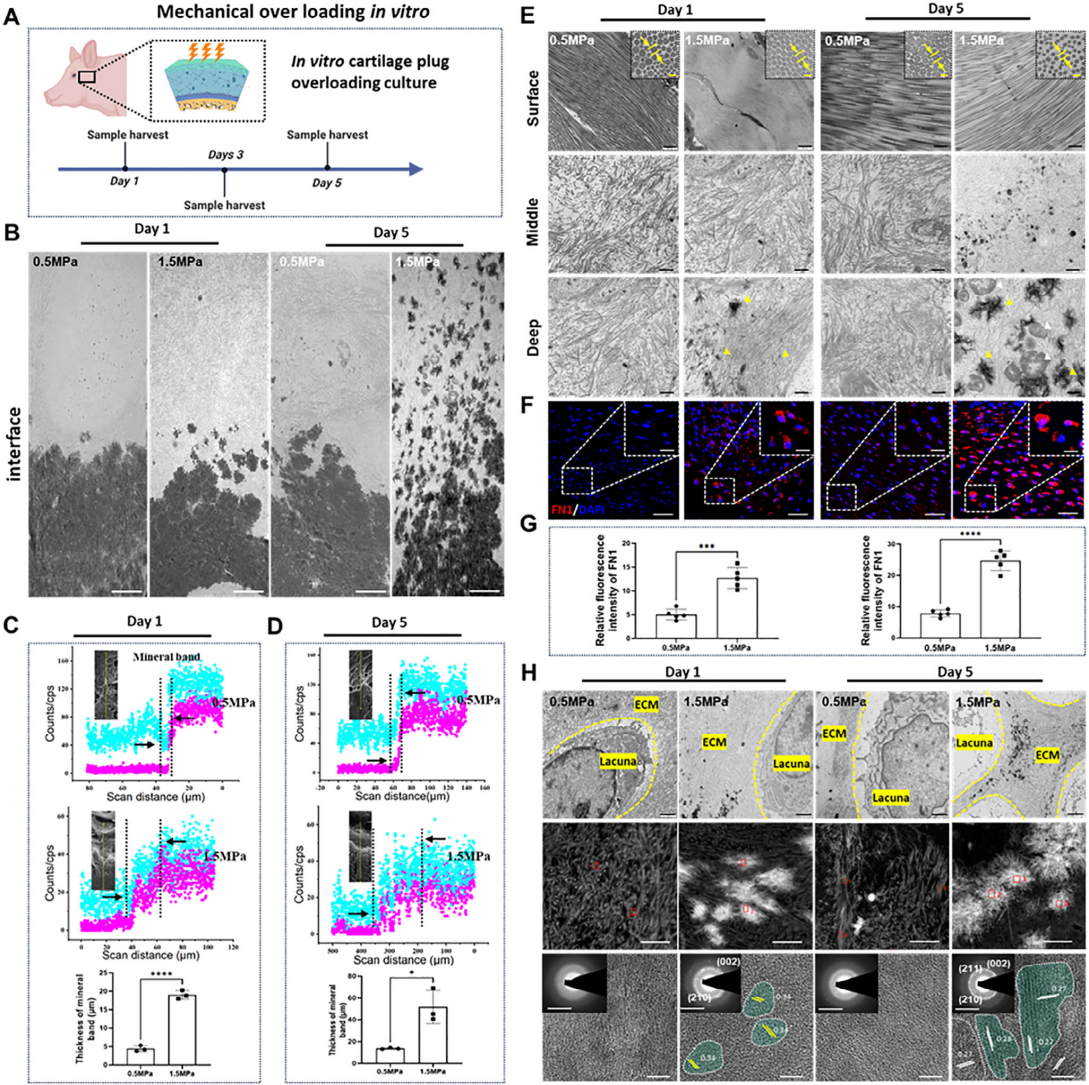
Mechanical overloading drives pathological mineralization via fibronectin upregulation. (A) Schematic diagram of in vitro cartilage plugs loading culture of porcine TMJ. (B) TEM images showing the structure of osteochondral interface in 0.5 and 1.5 MPa conditions. Yellow arrow indicated mineral crystals. Scale bar 2 µm. (C,D) SEM–EDS line scan to compare the thickness of mineral band from interface to overlying cartilage in 0.5 and 1.5 MPa conditions (*n* = 3). Results are presented as means ± SD, *p < 0.05 and ****p < 0.0001; statistical significance was calculated using two‐tailed Student's *t*‐test for multiple comparisons, all tests were two‐sided. (E) TEM images of the OA cartilage in 0.5 and 1.5 MPa conditions after 1 day to 5 days. Scale bar, 500 nm. High magnification of the area in surface layer showed cross section of collagen fibrils. Yellow arrow in surface indicates diameter of fibril, yellow arrow in deep indicates mineral crystals. Scale bar, 100 nm. (F) Immunofluorescence microscopy of the acetylation of fibronectin in chondrocytes in front of osteochondral interface. Scale bar, 50 µm. High magnification of the area, scale bar, 10 µm. (G) Quantitative analysis of the fluorescence intensity of fibronectin in (F) (*n* = 3). Results are presented as means ± SD, ***p < 0.001, and ****p < 0.0001; statistical significance was calculated using two‐tailed Student's *t*‐test for multiple comparisons, all tests were two‐sided. (H) TEM, HAADF (high‐angle annular dark field), HRTEM, and SAED images showed the mineral crystals deposited in the lacuna of hypertrophic chondrocytes in front of interface in 0.5 and 1.5 MPa conditions after 1 day to 5 days. TEM images scale bar 2 µm, HAADF images scale bar 200 nm, HRTEM images scale bar 5 nm, SAED images scale bar 5 1/nm.

To further verify the microstructural changes in in vivo cartilage OA progression mediated by mechanical overloading and its regulation of pathological mineralization, we performed in vitro porcine TMJ condylar cartilage plugs in vitro loading culture (Figure 7A and Figure S30). The results confirmed mechanical overloading (1.5 MPa) induced OA‐like mineralization patterns within 5 days, characterized by: (i) bottom‐up mineral progression from the interface (*p* < 0.001 vs 0.5 MPa controls); (ii) surface fibril disorganization (diameter reduction 38 ± 5%, *p* = 0.002); and (iii) microstructural changes mirroring in vivo OA (Figure 7B–D and Figure S31). Overloading conditions triggered interface perturbation within 24 h, with nanocrystal deposition in deep matrix layers, while surface/middle layers remained intact (Figure 7E and Figure S32). Immunofluorescence revealed concomitant fibronectin upregulation (3.1 ± 0.4‐fold, *p* = 0.003) and α‐tubulin downregulation (57 ± 6%, *p* < 0.01) (Figure 7F,G and Figure S33). Hypertrophic chondrocytes adjacent to the interface accumulated mineral crystals (5 ± 1 crystals/cell at day 1, increasing to 18 ± 3 by day 5, *p* = 0.001) under 1.5 MPa loading (Figure 7H).

### 2.8 Overloading Elimination Reverses the Mineralization and Alleviates OA Progression

**FIGURE 8:**
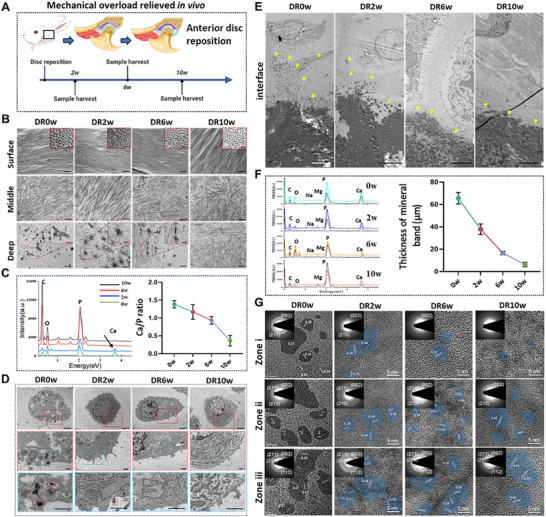
Overloading elimination reverses the mineralization and alleviates TMJOA progression. (A) Schematic diagram of in vivo displaced disk repositioned to eliminate the mechanical overloading. (B) TEM images showing the tempo‐spatial structure of overlying cartilage after displaced disk reposition. Scale bar, 200 nm. High magnification of the area in surface layer showed cross section of collagen fibrils. Scale bar, 100 nm. High magnification of the area depicted by the red rectangles showed calcified particulates and mineralized deposits. Scale bar, 100 nm. (C) TEM–EDS showing the weakening of mineralization in deep cartilage layer. (D) TEM images showed the structure of typical chondrocyte in front of mineral interface after disk reposition. Scale bar 5 µm. High magnification image showed local interesting area of MVs secretion. Red rectangles scale bar 500 nm, blue rectangles scale bar 200 nm. (E–G) TEM, TEM–EDS, HRTEM, and SAED images showing the reduction of mineralization from osteochondral interface after disk reposition. Yellow arrow indicated mineral crystals. TEM in E scale bar 10 µm, HRTEM scale 5 nm, SAED scale 5 1/nm.

From the perspective of above microscopic analysis, we found that the key to OA is mechanical overloading mediated osteochondral interface instability and ectopic deposition of interface‐derived nanocrystal fragmentation. Pathological cartilage mineralization initiated from the osteochondral interface to the overlying cartilage in OA‐A and further deteriorates the microscopic phenotype of cartilage. Therefore, to investigate the reversal of OA, we used a surgical model of disk repositioning (OA‐E) to eliminate joint overloading (Figure 8A). Results showed that cartilage mineralization significantly decreased, interface tend to completely restructure, simultaneously after displaced disk repositioned (Figure 8C,E–G). TEM images of the overlying cartilage showed that the middle layer cartilage fibrils restored disordered arrangement and surface fibrils orientated coherent ray pattern (Figure 8B and Figure S34). Meanwhile, the morphology and structure of chondrocytes in front of osteochondral interface area gradually returned to stable, and the secretion of calcified vesicles were significantly reduced (Figure 8D). Histology and mineral distribution analysis showed that OA cartilage was also reversed after disk reposition, the width of the mineral distribution was significantly reduced (Figures S35 and S36). MMP‐13 and decorin were widely regarded as key proteins in matrix degradation and collagen orientation regulation. During OA development, immunohistochemical staining results showed that expression of MMP‐13 was gradually increased in the surface layer, while decorin was significantly decreased in the middle layer (Figure S37). However, the opposite trend was observed in the displaced disk repositioned model (Figure S38).

## 3 Discussion

OA remains a highly prevalent and clinically intractable disease worldwide, underscoring the critical need to elucidate its pathological progression mechanisms [10, 40]. Although the histopathological hallmarks of OA are well characterized, the precise spatiotemporal sequence of phenotypic development and its underlying structural dynamics at high resolution remain poorly understood. To address these knowledge gaps, we developed a complete cycle rabbit TMJOA model driven by ADD‐induced mechanical overloading, complemented by comparative analyses of TMJOA and knee OA cartilage samples across four additional species (mice, rats, pigs, and humans). Although the TMJ and knee articular cartilage exhibit distinct structural compositions, and TMJ condylar cartilage shows significant interspecies variations, the osteochondral interface, which integrates overlying cartilage with subchondral bone, likely represents their most universal structural feature [41, 42]. Our previous studies of physiological osteochondral interfaces revealed that this transitional zone serves as both a mechanical pivot point and mineralization frontier, where the exquisite integration of mineralized structure and mechanical properties appears crucial for maintaining cartilage homeostasis [50]. We propose that mechanical overloading induced disruption of this mineralization‐mechanics coupling may represent a key initiating factor in osteoarthritis pathogenesis. Through this integrated multispecies approach, we systematically mapped the hierarchical structural evolution and compositional dynamics spanning nano to micro scales in OA pathogenesis. These findings provide evidence for osteochondral interface disruption as a potential initiating event in mechanical overloading induced OA, while also revealing novel molecular mechanisms that may contribute to OA progression.

Heterogeneity of OA phenotypes is largely determined by regional variations in articular cartilage, including differences in cell types and ECM structural components [16]. OA cartilage is characterized by matrix degradation, pathological mineralization, and SB remodeling, driven by metabolic imbalances in chondrocytes across different regions [7, 38, 40]. These pathological changes arise from complex interactions between chondrocytes and the ECM, as well as between organic and inorganic components within cartilage [43, 44]. Histologically, OA progression is marked by decreased expression of COL II and aggrecan alongside increased COL X and calcification‐specific proteins [21, 45]. However, while end‐stage OA is defined by advanced matrix breakdown and osteophyte formation, the underlying mechanisms driving these changes remain poorly understood. A deeper investigation into the early events preceding macroscopic cartilage lesions, particularly the material properties of affected tissues, could provide critical insights into OA pathophysiology [45]. Materials science approaches have significantly advanced our understanding of healthy cartilage, revealing its hierarchical structure and the mechanisms governing its mechanical and chemical properties during load dissipation [45, 46, 47, 48]. However, similar analyses have not been applied to OA cartilage, despite their superficial similarity to physiological cartilage. Adopting a materials science approach, we analyzed the material comprising OA cartilage lesions in vivo by examining overloading induced OA models in three different species and in human and porcine TMJOA samples. Combining SEM–EDS, TEM–EDS, SAED, HRTEM, and micro‐Raman, we first mapped spatiotemporal nano‐micro scale phenotype atlas of OA cartilage. In our study, nano mineral crystals deposited in ECM of deep cartilage was the earliest change in the nano‐micro structure and composition, prior to ECM degradation. TEM observations suggested the presence of Ca‐ and P‐rich nanocrystals with trace amounts of Na and Mg near but separate from the osteochondral interface, exhibiting morphologies resembling bone mineral clusters. Notably, while previous studies have often described cartilage mineralization as a late‐stage OA feature [49, 50], our high‐resolution analysis identified it as an early event, likely overlooked in past work due to limited nanoscale resolution and a focus on superficial cartilage changes. As OA progressed, nanocrystal accumulation in deep cartilage formed dense calcified plaques, while middle and superficial layers exhibited collagen fibril disorganization and matrix degradation. Intriguingly, mineralization was confined to deep cartilage, possibly due to its proximity to the osteochondral interface and region‐specific chondrocyte behavior [51]. Collagen fibrils, the fundamental units of cartilage ECM, underwent distinct zonal changes: surface fibrils unraveled and degraded into filamentous debris, while middle layer fibrils increased their alignment, likely compensating for the loss of mechanical integrity in degenerating cartilage.

Spatiotemporal heterogeneity of nano‐micro scale phenotypes in OA cartilage creates distinct mechanical responses, representing the tissue's adaptation to altered energy dissipation following overloading [17]. In early OA‐E, we observed significant mechanical enhancement in deep cartilage at both micro‐ and nanoscales, primarily due to nanocrystal deposition within the collagen fibril network, a reinforcement mechanism analogous to concrete structures. This observation appears consistent with reports from unilateral anterior crossbite rat models showing mechanical overloading associated deep cartilage mineralization and ECM stiffening [52]. However, this increased stiffness reduces mechanical heterogeneity in deep cartilage, leading to localized stress concentrations, impaired force transfer, and microcrack formation key early events in OA‐E pathogenesis [31]. These microcracks may propagate into the SB, potentially disrupting cartilage‐bone crosstalk and contributing to impaired tissue homeostasis and OA progression [20, 31]. Furthermore, deep cartilage mineralization reduces overall cartilage thickness, creating a vicious cycle of worsening mechanical overloading and continued disease advancement [50]. However, in the mechanical characterization component of this study, both nanoindentation and AFM were employed to assess the mechanical properties across the osteochondral interface. It should be noted that these techniques may lead to potential overestimation of strains and deviations from small‐strain theory, particularly in highly mineralized regions. Our methodology represents a deliberate trade‐off between achieving depth‐uniform measurements and minimizing local artifact risks. Consequently, we emphasize that our mechanical conclusions primarily focus on revealing relative mechanical gradients across tissue interfaces rather than providing absolute mineral‐phase property measurements.

The osteochondral unit comprising articular cartilage, SB, and their interface functions as an integrated biomechanical composite essential for load distribution during joint movement [7]. Although alterations in any component can disrupt joint integrity, the precise spatiotemporal sequence of pathological changes in OA remains an active area of investigation [17, 21]. Current evidence presents multiple plausible initiation sites including articular cartilage, osteochondral interface, and SB [7], with the relative importance of each likely varying across different OA subtypes and etiologies. While much attention has historically focused on the articular cartilage or SB in isolation [9, 11, 16], emerging data, including our previous work [53], highlight the osteochondral interface as a potentially critical but understudied component that may play particularly important roles in mechanical overloading induced OA. However, OA‐induced changes in cartilage mechanics compromise this function [16]. Clinical observations in obesity‐related OA reveal horizontal interface fissures resulting from mechanical mismatch: cartilage's higher Poisson's ratio causes greater lateral deformation under compression, constrained by the underlying mineralized tissue, generating destructive shear forces [31]. Our observations in mechanical overloading models are consistent with this proposed mechanism. We observed interface microfissures in early‐stage OA (OA‐E) accompanied by adjacent nanocrystal deposition that spread upward through the deep cartilage, potentially explaining histological observations of tidemark duplication. Supporting this, finite element analysis in disk displacement models demonstrated initial stress concentration at the osteochondral interface, while F‐CHP probes detected molecular‐level fibril damage at this very site. This process may contribute to mineral disintegration and altered cartilage‐SB crosstalk [53], suggesting a potential pathway for OA development in mechanical overloading models. The distinct mineralization patterns we observed may represent a characteristic response to mechanical stress, differing from inflammation‐driven or metabolic OA pathways. However, we emphasize that these observations do not exclude other initiation mechanisms that may operate in different OA contexts (e.g., inflammation‐driven or metabolic OA), nor do they preclude the possibility of parallel pathological processes originating from multiple joint tissues simultaneously. Our conclusion that deep zone mineral deposition is an early event primarily rests on its detection being the earliest pathological change observed during the OA‐E stage (1, 2, and 4 weeks post‐mechanical overloading), preceding matrix degradation in the superficial and middle zones. However, we must emphasize several caveats. Labeling this as the absolute initiating event requires stronger evidence. The temporal resolution of our experiments is on the scale of weeks, which may miss the earliest molecular or structural alterations occurring within hours to days after mechanical overloading. Given that mechanical overload acts first on the stiffer subchondral bone rather than the softer cartilage, early microtrauma of trabeculae or accelerated bone remodeling could plausibly precede nanocrystal deposition. Such events might even be the direct cause of the interface integrity loss and the subsequent release of mineral fragments. This possibility warrants further investigation in future studies.

Emerging evidence suggests that pathological cartilage mineralization could play a role in OA development, potentially acting as more than just a terminal disease manifestation [23, 26]. As show in SEM images in multiple publications by Boyde [3], the mineralized tidemark appears to become contiguous with the spherites in the soft cartilage. Thus, it appears that perhaps the hypertrophic chondrocytes may initiate mineral crystals but then these crystals enlarge with the minerals provided by fluid from the bone. While the mineral source remains controversial, our findings suggest the osteochondral interface serves as a key origin point [53]. In OA‐E, we observed mineral crystal perturbation at this interface, representing the potential initial pathological crosstalk between cartilage and subchondral bone that connects subsequent lesions in both tissues. Although hypertrophic chondrocyte‐mediated mineralization, characterized by Mg‐rich spherical particles likely derived from vesicles, has been well‐documented in OA [28, 51], our results reveal a more complex picture with stage‐dependent variations in mineralization mechanisms. While OA‐A involves cell‐mediated mineralization via LC_3_^+^ autophagic calcified vesicles [29], OA‐E mineralization occurs independently of chondrocyte activity. HRTEM and SAED analyses confirmed this distinction: OA‐E nanocrystals structurally resembled interface bone minerals, whereas OA‐A featured highly mineralized, chondrocyte‐derived deposits. This stage‐dependent mineralization pattern may reflect variations in biomineralization processes, potentially involving multiple pathways of crystal nucleation, growth, maturation and carbonization that could be influenced by noncollagenous proteins and other local factors [54, 55]. Chondrocyte‐derived minerals require carbonic anhydrase‐mediated carbonation for ECM integration [56], explaining the predominance of carbonaceous phosphate in OA‐E versus highly mineralized phosphates in OA‐A. These findings were further supported by differential expression of autophagy markers (LC_3_, α‐tubulin) between OA stages [29]. Concurrent ECM remodeling during OA progression creates a permissive environment for mineralization: degradation of inhibitors (proteoglycans, collagen‐II) alongside increased promoters (collagen‐I/X, Runx‐2) [23], disrupts collagen architecture [48] while elevating local Ca^2+^ and PO_4_^3−^ concentrations [57]. The resulting mineral deposits then propagate disease through crystal‐induced chondrocyte stress and hypertrophy [58], with hydroxyapatite crystal inflammation potentially amplifying mineralization via secondary cell‐mediated processes [51]. Fibronectin serves as a critical bridge anchoring chondrocytes to the ECM. Through integrin‐mediated binding to fibronectin, chondrocytes perceive key mechanical and topographical cues from the ECM. Previous studies have demonstrated that fibronectin is closely associated with pathological mineralization in connective tissues such as blood vessels and tendons. Fibronectin promotes the expression of osteogenesis‐related genes and facilitates calcification progression via mechanotransduction signaling pathways [59, 60, 61]. However, the bottom‐up mineralization pattern revealed in our results is likely closely related to the unique cartilaginous origin of the TMJ, which may differ fundamentally from large joints such as the knee. As a secondary cartilage, the TMJ derives from a distinct embryonic origin compared to the primary hyaline cartilage of the knee and possesses continuous growth and remodeling capacity [62]. Although the knee OA models of DMM recapitulate some similar pathological hallmarks, they are not sufficient to claim an identical disease‐initiating sequence to the TMJ.

The mechanical properties of the OA ECM undergo significant alterations, as demonstrated in our results, profoundly influencing chondrocyte phenotypic transitions [14]. Our study reveals that nanocrystal deposition in deep cartilage during OA‐E creates a stiffer matrix microenvironment near the osteochondral interface. This ECM stiffening represents a crucial mechanistic link that triggers subsequent pathological responses, including the release of calcified MVs from chondrocytes that promote cartilage mineralization and OA progression. Chondrocytes sense these mechanical changes through specialized cell‐ECM adhesions and mechanosensing structures [13], with proteomic analysis identifying fibronectin and vitronectin as key proteins in this process. These linker molecules mediate integrin‐ECM connections [63, 64], facilitating mechanical signal transduction from the extracellular environment to intracellular compartments, as confirmed by both proteomic and immunofluorescence data. The upregulated expression of fibronectin and vitronectin in OA‐E enhances chondrocyte anchoring within lacunae [63], while phalloidin staining and TEM observations demonstrate cytoskeletal reorganization and increased extended extracellular synapses‐clear indicators of stiffness‐induced structural and functional adaptations in chondrocytes. The released calcified MVs, which contain autophagy‐related proteins like LC_3_^+^ and exhibit autophagosome‐like dimensions [29], point to an autophagy‐mediated mineralization process. This secretory autophagy represents a precisely regulated mechanism operating in both physiological and pathological contexts, with microtubule stability serving as a critical control point [29]. As essential cytoskeletal components, microtubules coordinate organelle positioning and autophagosome‐lysosome fusion [65]. Their destabilization in OA disrupts autophagic flux, promoting calcified vesicle release [29] and thereby establishing a direct connection between ECM mechanical changes and pathological mineralization. This mechanobiological cascade may contribute to the characteristic phenotypic shifts in chondrocytes during OA progression.

While numerous in vivo and in vitro studies confirm mechanical overloading's crucial role in OA pathogenesis, most research has focused on chondrocyte signaling pathways, demonstrating how mechanical stimuli shift cartilage metabolism toward catabolic dominance and away from anabolic processes [13], ultimately leading to matrix degradation and tissue degeneration [13, 30]. However, our understanding remains incomplete regarding the immediate physical impacts of overload on the cartilage matrix itself structural alterations that occur before chondrocytes can detect and respond to mechanical stimuli [66]. This temporal disconnect is critical: while matrix changes happen instantaneously upon loading, cellular responses require time for signal transduction and protein synthesis through central dogma processes [67, 68]. To address this knowledge gap, we employed a combination of multispecies joint instability models and in vitro cartilage plug overloading systems. Our observations suggest that excessive mechanical loading may initially induce biomechanical alterations at the osteochondral interface, where disrupted interactions between organic and inorganic components could potentially lead to nanocrystal fracture and deposition in adjacent matrix regions. These early structural changes appear to modify the mechanical microenvironment of deep zone chondrocytes, possibly contributing to the establishment of conditions that may promote subsequent cartilage mineralization and OA progression in our experimental models. It is important to note that our observations are derived from mechanical overloading‐induced OA models (ADD and DMM). In other OA subtypes, such as those driven primarily by metabolic dysregulation or inflammation, the initial insult and pathological sequence may indeed follow a different spatiotemporal pattern.

Arthroscopic anchored repair of meniscal and joint disk tissues has emerged as an important clinical strategy for early OA intervention [33, 36]. This minimally invasive procedure involves arthroscopically repositioning displaced joint disks or menisci and securing them to the articular cartilage surface, thereby reducing mechanical loading and frictional stress on cartilage surfaces [35]. While this approach has demonstrated clinical efficacy in alleviating OA symptoms and slowing disease progression [35], the precise mechanistic basis for these therapeutic effects remained unclear. Our study provides novel nano‐micro scale insights into this clinical intervention by performing joint disk repositioning in OA‐E models following comprehensive microscopic characterization. The results demonstrated that eliminating mechanical overloading through this procedure may lead to three key therapeutic effects: (1) reduction of interface mineralization, (2) attenuation of bottom‐up mineralization patterns, and (3) decreased secretion of calcified vesicles by chondrocytes. Importantly, these changes were associated with significant improvement and stabilization of cartilage microstructure. These findings provide initial insights into potential nano‐micro architectural mechanisms underlying the therapeutic effects of meniscal or joint disk repositioning. Importantly, our in vivo observations after disk repositioning, which show reduced interface mineralization and top‐down crystal regression, present a key unresolved question. Specifically, it remains unknown whether these crystals are actively cleared by cells (e.g., chondrocytes or osteoclasts) or simply encapsulated by new matrix to form a stable interface. This critical distinction cannot be resolved with our present results and requires future study.

This study reveals overloading's role in osteochondral mineralization, yet key questions remain. Six critical areas require investigation: (1) the reversibility of overloading induced mineralization, necessitating longitudinal recovery studies; (2) development of more physiologically relevant models incorporating systemic factors to bridge the in vitro‐in vivo gap; (3) expansion of sample diversity and size to improve translational validity; (4) longer‐term observations to distinguish transient mineralization from progressive pathology; (5) While our data demonstrate osteochondral interface disruption in mechanical overloading models, we recognize that other OA subtypes may follow different initiating sequences, potentially involving synovial inflammation or chondrocyte senescence as primary drivers; and (6) While we identified a novel mechanism in the TMJ, definitive parallel testing in the knee joint with the same depth of analysis was beyond the scope of this paper and a side‐by‐side comparative investigation of the osteochondral interface in different joint types is need to represent an essential and logical next step for the field. Addressing these gaps may advance our understanding of OA pathogenesis and inform therapeutic development.

Interface‐derived nanocrystal fragmentation and ectopic deposition induced by mechanical overloading initiates OA. Nanocrystals deposition in deep cartilage ECM changed mechanical microenvironment of hypertrophic chondrocytes in front of osteochondral interface. Upregulated fibronectin and vitronectin promoted calcified MVs secretion, which induced bottom‐up ECM mineralization and OA progression.

**FIGURE 9:**
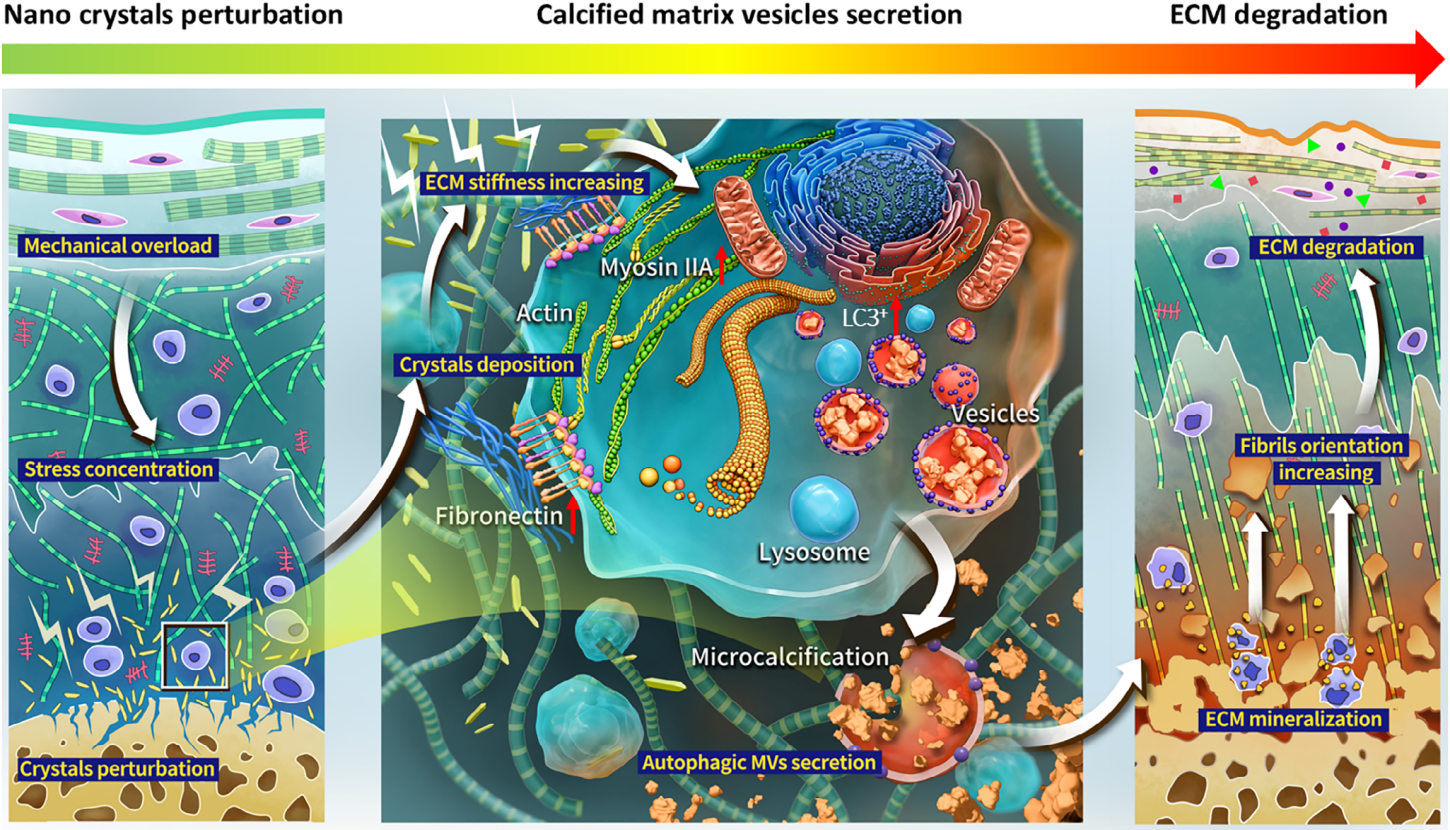
Schematic diagram showing the synopsis of the findings. Interface‐derived nanocrystal fragmentation and ectopic deposition induced by mechanical overloading initiates OA. Nanocrystals deposition in deep cartilage ECM changed mechanical microenvironment of hypertrophic chondrocytes in front of osteochondral interface. Upregulated fibronectin and vitronectin promoted calcified MVs secretion, which induced bottom‐up ECM mineralization and OA progression.

In summary, the current study provides evidence that perturbations in nanomineral crystals at the osteochondral interface could participate in the initiation of pathological cartilage mineralization during OA development, possibly influencing the emergence of disease‐associated nano‐micro scale tissue alterations. Deposition of nanocrystals in deep ECM induced mechanical stiffness increasing, which triggered chondrocyte mechanosensing. Subsequently, release of calcified MVs was facilitated and further promoted the bottom‐up mineralization of ECM and OA progression (Figure 9). The findings from this study add to the growing body of knowledge about OA progression mechanisms and may provide considerations for future therapeutic research. While our multimodel approach (ADD/DMM) reveals consistent mineralization patterns in mechanical overloading contexts, further studies are needed to examine these phenomena in other OA subtypes.

## 4 Materials and Methods

### 4.1 Study Design

The rabbit TMJOA model served as the primary experimental system throughout this study. Additional models, including human and porcine samples, were employed solely for supplementary validation of Result 1, specifically to assess the cross‐species and interorgan universality of OA nano‐micro architectural phenotypes. TMJOA and knee OA animal models were constructed by ADD and DMM in three different species (mouse, rat, and rabbit). Human and porcine TMJOA condyles were collected by clinical end‐stage OA patients with joint replacement and heads of porcine from local abattoir. The study was approved by the ethics committee of the Sichuan university West China Hospital of Stomatology (WCHSIRB‐D‐2023‐583, WCHSIRB‐D‐2022‐246, and WCHSIRB‐CT‐2022‐132). The cartilages were harvested and histologically stained to identify different OA stages. First, nano‐micro structural (TEM, SEM, AFM, SAED, and micro‐CT), compositional (Raman, TEM–EDS, and SEM–EDS), and mechanical (Nanoindentation and AFM) characteristics from surface to deep regions of cartilage were analyzed to study the stages‐specific and location‐specific process in OA cartilage. Second, osteochondral interface was focused and explored its underlying function of OA initiation. TEM and Raman were used to track the nano mineral crystals from the osteochondral interface at different OA stages. SEM–EDS line scanning was used to quantify the width of the bottom‐up mineralization advancing from the interface to the overlying cartilage. Furthermore, F‐CHP and FEA were applied to feature the collagen triple helix injury and mechanical distribution at osteochondral interface, respectively. Third, HRTEM and SAED were used to reveal the assembly patterns of mineral crystals in different OA stages, which was to clarify the original resource of crystals in OA‐E. Meanwhile, elastic modulus of chondrocyte lacuna was tested by AFM‐indentation to reveal the mechanical microenvironment alteration induced by nanocrystals deposition. Fourth, proteomics was used to screen the target proteins up‐regulated by chondrocytes, which was bridged cartilage mineralization from OA‐E to OA‐A. Fifth, cartilage plugs were in vitro loading cultured in Flexcell FX5000 Compression system to stimulate the mechanical overloading condition of in vivo articular cartilage, which was to validate the process of OA initiation. Finally, displaced disk repositioned in vivo was applied to eliminate the mechanical overloading and expected to reverse the OA development. The experimental scheme is illustrated in Figure S1. More detailed experimental methods and procedures are provided in Supporting Information.

### 4.2 Statistical Analysis

Preprocessing procedures for data were performed prior to statistical analysis, including transformation of nonnormal data, normalization of variables to a common scale, and outlier detection using the interquartile range method. Experimental results were expressed as mean ± SD. The sample size (*n*) for each statistical analysis refers to the number of independent biological replicates, with specific values indicated in the figure legends (e.g., *n* = 3 represents three independent samples). Statistical differences were assessed using one‐way analysis of variance (ANOVA) followed by Tukey's post‐hoc test for multiple comparisons, and two‐tailed Student's *t*‐test for pairwise comparisons. The significance level (alpha value) was set at 0.05. Prior to analysis, the assumptions for ANOVA (normality via Shapiro–Wilk test and homogeneity of variance via Levene's test) were verified, and data meeting these assumptions were included. Statistical significance was defined as *p < 0.05, **p < 0.01, ***p < 0.001, ****p < 0.0001, and ns represents no significant difference. All statistical analyses were performed using GraphPad Prism version 8.0 (GraphPad Software, Inc., CA, USA).

## Author Contributions

N.J and R.R. contributed equally to all of this study, including the experimental performing, data acquisition and analysis, and manuscript drafting. Z.S, P.Y.C., and S.C.Z. contributed to data interpretation. R.R. and J.H.Z. contributed to animal experiments. R.R. and J.H.Z. contributed to electron microscopy experiments. Z.L. contributed to manuscript revision. C.L. and S.S.Z. contributed to the study conception and design, data interpretation, and manuscript revision. All authors have read and approved the current version of the manuscript.

## Funding

This study was supported by grants from the National Key R&D Project of China, Ministry of Science and Technology of the People's Republic of China [Grant No. 2023YFC2509200], the National Natural Science Foundation of China [Grant Nos. 82571123, 82501190, 82301109], and the Sichuan International Science and Technology Innovation Cooperation Project [Grant No. 2025YFHZ0191].

## Ethics Statement

The study was approved by the ethics committee of the Sichuan university West China Hospital of Stomatology (WCHSIRB‐D‐2023‐583, WCHSIRB‐D‐2022‐246, and WCHSIRB‐CT‐2022‐132).

## Conflicts of Interest

The authors declare no conflicts of interest.

## Supporting information




**Supporting File**: advs74001‐sup‐0001‐SuppMat.docx.

## Data Availability

The data that support the findings of this study are available from the corresponding author upon reasonable request.
