# Supplementary material for: Mechanical Overloading‐Induced Nanomineral Crystal Perturbation from the Osteochondral Interface: A Potential Initiator of Osteoarthritis
Source: Adv Sci (Weinh). 2026 Jan 26;13(27):e16893. doi: 10.1002/advs.202516893 (PMC13170212; doi:10.1002/advs.202516893)
Supplement: Supplementary file 1 — Supporting File: advs74001‐sup‐0001‐SuppMat.docx. [file ADVS-13-e16893-s001.docx]

Supplementary Materials for

Mechanical Overloading-Induced Nanomineral Crystal Perturbation from the Osteochondral Interface: A Potential Initiator of Osteoarthritis

Nan Jiang^1, 2^†^,^ Rong Ren^1, 2^†, Zhan Su^1, 2^, Jiahao Zhou^1, 2^, Pinyin Cao^1, 2^

Sicheng Zhang^3^, Zhen Li^4^*, Chao Li^3^*, Songsong, Zhu^1, 2^*

*Corresponding author. Email: zss_1977@163.com (SS, Zhu), lichao@scszlyy.org.cn (C, Li), zhen.li@aofoundation.org (Z, Li)

**Supplementary Materials**

Materials and Methods

Supplementary Text

Figs. S1 to S38

Table. S1

**Materials and Methods**

Group designation and experimental scheme are described in Fig. S1.

**Animal OA model**

**1. ADD induced TMJOA and DMM induced knee OA model**

Male New Zealand white rabbits (6 months old; weight, 1.5 to 2.0 kg), male Sprague-Dawley rats (6 weeks old; weight, 140 to 160 g), male C57 mouse (6 weeks old; weight, 70 to 90 g) were provided by the animal center of the Sichuan University. All experimental protocols met the National Institutes of Health Guidelines for the Care and Use of Laboratory Animals and were reviewed and approved by the Institutional Animal Care and Use Committee of the Sichuan University. ADD and DMM procedures were used to induce osteoarthritic-like changes in the left TMJ and knee joint cartilage of above three species. The experimental observation time points were set at 0 weeks post administration add0w, add2w, add4w, add8w, and add12w. OA Stages estimated by ORASI score system using HE staining image. Normal was defined as add0w, OA-early (OA-E) was defined as add2w and add4w, and OA-advanced (OA-A) defined as add8w and add12w. There was n=3 biological replicates in every time point. Control groups were subjected to the same procedure but without disc and meniscus displacement. All experimental animals were sacrificed humanely using anesthetic overdose, following established ethical standards for animal experimentation. The rabbit ADD-TMJOA model was employed as the primary experimental model in this study due to several key advantages: its moderate body size, close anatomical similarity of TMJ structure to humans, excellent consistency in model establishment, and optimal observation time window, making it a highly suitable in vivo animal model for this research. Other two rodents OA models were used for validation the nano-micro phenotypes universality.

**2. Porcine TMJOA cartilage preparation**

In conducting condylar samples acquisition form a local abattoir, we found a certain proportion of condyles with TMJOA, which were used as porcine-derived OA samples. TMJOA condyle samples were dissected from the heads of porcine (male, aged 7~8 months, n=3). Before the next high-resolution analysis, all tissues were confirmed by histological analysis and then dissected into small pieces and washed with sterile phosphate-buffered saline (PBS) before storage at -80 ℃ when not undergoing treatment and analysis. The collected samples were then used for the high-resolution characterizations to validation of nano-micro structural phenotypes in porcine TMJ cartilage.

**3. Human TMJOA cartilage sample preparation**

All samples of TMJOA cartilages were collected from patients undergoing artificial TMJ replacement surgery in Department of Orthognathic and TMJ Surgery, Sichuan University West China Hospital of Stomatology (n=3). Informed consent was obtained from all patients. All experimental procedures were approved by Ethics Committee of West China Hospital of Stomatology, Sichuan University (Approval No. WCHSIRB-D-2022-246 & No. WCHSIRB-CT-2022-132). The load-bearing middle region of the anterior slope of condyles was carefully collected, which contained articular cartilage, interface, and SB plates (size: ~0.5 cm × 0.5 cm, depth: 4-5 mm), washed with sterile phosphate-buffered saline (PBS), and stored at -80 ℃ for further treatment. The collected samples were then used for the high-resolution characterizations to validation of nano-micro structural phenotypes in human TMJ cartilage.

**4. ADD repositioned model**

Only New Zealand white rabbits (6 months old; weight, 1.5 to 2.0 kg) were used for ADD repositioned model. ADD rabbits were randomly selected for disc repositioning surgery 2 weeks after the initial ADD surgery. After anesthesia by pentobarbital sodium injection through the marginal ear vein at 0.3% ml·kg^−1^ body weight (P3761, Sigma-Aldrich, USA), another incision was made along the previous incision, the TMJ capsule was exposed. The suture was carefully removed with the intact disc preserved. Then the displaced disc was released from adhesion and was pulled back on the top of the condyle. The muscle and skin were sutured. The re-sham group went through the second operation that exposed the TMJ capsule again without changing the disc position. The same postoperative care as initial surgery was provided.

**Histology**

All cartilage samples were fixed in 4% paraformaldehyde for 48 h, and decalcified in 10% EDTA-2Na (w/v) solution for at least 4 weeks. Then, all samples were dehydrated and embedded in paraffin. 7-μm thick sections were cut for, Hematein eosin (HE), Safranin-O (SO) staining and Alcian blue staining to assess OA histopathology according to the established scoring system. The system divided the samples into the main 4 stages (0-4), where normal cartilages were graded to be 0 and OA cartilages were graded as 1-2 (OA-E) and 3-4 (OA-A). Since the cartilage was almost denuded at grade 4, these samples were not considered for further materials analysis. Also, subgrade 0.5 was utilized in the grading.

For calcium distribution analysis, fresh condyle samples were fixed by 4% paraformaldehyde for 48 h, embedded in Methyl Methacrylate and then sectioned into 100-µm slices using a hard tissue slicer (SP1600; Leica). Sections were stained by von Kossa reagents (Ab150687, Abcam, USA) and Alizarin Red S staining (40 mM, pH 4.2; Millipore Sigma, Burlington, USA) for osteochondral interface thickness measurement.

**Immunofluorescent Staining**

Immunofluorescent analysis was performed according to a standard method. 7-μm thick sections were treated with a heat-induced antigen retrieval process in sodium citrate solution at 65 ℃ overnight, washed with PBS solution, and blocked with 2% (wt/v) bovine serum albumin (BSA, Sigma) for 1 h at room temperature. Samples were then incubated with primary antibodies overnight at 4 ℃, washed with PBS-Tween (0.01% Tween) and incubated with secondary antibodies at room temperature for 2 h. The following antibodies were used: anti-LC3B (1:200, bs-2912R), anti-α-tubulin (1:200, bsm-33039M), anti-fibronectin (1:200, bs-0666R), anti-vitronectin (1:200, bs-1932R), anti-myosin IIa (1:200, Cell Signaling Technology, 3403T) and secondary antibodies conjugated with Alexa Fluor 488 and 568 (1:200, Thermo Fisher Scientific). The imaging was performed with a confocal microscopy (FV3000, Olympus, Tokyo, Japan).

**F-CHP staining**

CHP labeled with the fluorophore 5-FAM (F-CHP) was obtained from 3Helix (catalog number: FLU300, Salt Lake City). All samples were sectioned in 5-μm slices for staining preperation. Stock F-CHP was diluted with PBS to a concentration of 20 µM. Because the CHP slowly self-assembles into its own triple helix over time, losing its driving force to hybridize with collagen, its solutions must be heated to dissociate the trimers into single CHP strands before use. Therefore, CHP solutions were heated for 6 min using a hot plate set to 70 °C and subsequently cooled on ice for 2 min to room temperature and immediately used for staining. Cartilage sections were incubated in 100 µL of the activated CHP solution for at least 1 hr at 4 °C. Samples were then rinsed (3 times in 300 µL of PBS for 10 min each at 4 °C) and photographs were taken by a confocal microscopy (FV3000, Olympus, Tokyo, Japan).

**Micro-computed tomography**

All TMJ samples were scanned using a high-resolution micro-CT system (Scanco, Switzerland). Briefly, specimens were scanned at 70 keV and 200 μA. Two-dimensional slices with an 8 μm isotropic resolution was generated and used for 3D reconstruction of the TMJ specimen.

**SEM and EDS analyses**

All OA cartilage cryo-cut samples were thawed at room temperature and washed with DI water several times, followed by dehydration through graded ethanol treatments (30, 50, 70, 80, 90, 95, 100, and 100 % (v/v)) for 15 min in each solution. Then, the samples were critical point dried (EM CPD300, Leica) and sputter coated with gold. Imaging was performed (Helios G4 UC, Thermo Fisher Scientific) at 15 Kv accelerating voltage. EDS line scan and point scan were performed to identify the element distribution and width of the osteochondral interface and local element composition. In each observation time point, nine replicates were used to minimize the impact of such errors on the data.

**Raman spectroscopy**

All fresh OA condyle samples were embedded by OCT and cryo-cut in 10 μm thickness sections and washed by PBS to remove the OCT. The sections were immersed in PBS and kept frozen at – 20°C until use. Sections were thawed to room temperature prior to spectra collection and kept moist by dripping PBS buffer during spectra collection. The Micro-Raman spectroscopy was performed to measure the composition and distribution of whole cartilage and osteochondral interface using a confocal Raman microscope (LabRAM HR Evolution, Horiba, France) equipped with a 532 nm laser and the laser spot size as 0.5μm. Spectra were collected in the 400~2000 cm^-1^ using an electron-multiplying charge-coupled device detector at a spectral resolution of ~5 μm^-1^. The characteristic peak of the amid I in collagen can be observed at 1247 cm^-1^ in the Raman spectrum. It represents the SO_3_^-^ stretch at 1490 cm^-1^, which can be regarded as the characteristic peak of proteoglycan. Phosphate ν1 stretching bands appeared at 960 cm^-1^ for HAP. Raman spectroscopy images were obtained by continuous scanning with the area of 50 × 300 μm with Step 5 μm. Each spectrum was collected with an acquisition of 1 s and 10 accumulations. No sample degradation was noticed using this parameter. The peak heights of 1247 cm^-1^, 1490 cm^-1^ and 960 cm^-1^ were measured and to compare the changes of collagen, proteoglycan and HAP, respectively. The LabSpec6 was used for handling spectra baseline correction, background subtraction and normalization.

**AFM**

The Force-Displacement (FD) measurements of the AFM were performed to describe the nanomechanical properties of OA cartilage tissue. 50-um-thick cryo-cut samples were washed and immersed in PBS solution for further evaluation. We used a spatially controlled instrument (Dimension ICON, Bruker Nano) and Si cantilevers (SCANASYST-AIR, Bruker, USA) with a tip radius ≈ 50 nm, a determined spring constant 41.61 N/m, and a back angle α = 35°. Measurements for a 256×256 array of FD data were taken of three different OA regions in 5×5 μm^2^ large area. The tip approach speed was 1 μm/s until a force of 25 nN was reached, followed by tip retraction at the same speed. Hertz model with a conical tip was assumed to analyze the FD data to fit Young’s modulus. From maps of FD data, we wrote our MATLAB scripts to construct profiles of the indentation modulus. Due to the limitations of the testing machine, all measurements were operated in air. To ensure measurement accuracy, we monitored AFM tip wear by regularly examining tip geometry and sharpness using standardized calibration samples (e.g., mica or silicon) before and after experiments. A strict tip replacement protocol was implemented when wear or damage was detected. Additionally, we verified data consistency by comparing force-distance curves from the beginning and end of each experiment, excluding any data showing significant deviations indicative of tip wear.

**Nanoindentation**

Fresh cartilage tissues were dissected into small pieces about 2 mm-thick along the longitudinal direction of the osteochondral interface, and immersed in PBS solution for further measurement. Indentation studies were performed with a nano-indenter (PIUMA, Optics 11, Amsterdam Netherlands). A spherical nanoindentation probe with a radius of 8.5 μm and a stiffness of 50.57 N m^-1^ were used, respectively, with an indentation depth of 5000 nm. The loading and unloading rates were set by 2500 nm/s, and hold times was set by 1s. The specific waveform was force-displacement-time curve. To map the modulus transition of the whole thickness cartilage, a nanoindentation test matrix (step: x and y-50×300 μm) was performed from surface to subchondral region and the step size was set by 30 μm. Nanoindentation line scan tests were performed along the slice from the cartilage surface to the SB. To ensure reliability, we manually excluded all curves exhibiting deviations from typical elastic-plastic behavior (e.g., sudden displacement bursts, irregular unloading slopes). This rigorous curation ensured that only data adhering to Hertzian contact mechanics assumptions were retained. Using the Hertz model, the effective Young’s modulus was calculated from the slope of the unloading curve by a power law fit to 20–90% of the unloading curve. Energy dissipation can be calculated by determining the area between the loading and unloading curves. To mimic the physiological environment, all measurements were operated in PBS solution.

**TEM, EDS, HRTEM and SAED analyses**

The cartilage samples (1-2 mm, including osteochondral interfaces, overlying cartilage and SB) were fixed in 2.5% glutaraldehyde solution for 24 h, washed with PBS solution, stained with 1% OsO4, dehydrated, and embedded in a spur resin. The specimens were next cross-sectioned in Lerca EM UC7 ultratome at 70 nm onto bare 200-mesh copper grids and imaged in an aberration corrected scanning transmission electron microscope (FEI Titan G2 80-200 microscope equipped with a Super-X EDS detector) at 200 kV. HAADF-TEM, TEM-EDS mapping, and SAED patterns were performed at regions of interest. Each spectrum was calibrated, normalized, and background subtracted and processed by principal component analysis. Before each experiment, the electron probe of the microscope was aligned with a DCOR plus spherical aberration corrector using a gold standard sample. No obvious damage was observed during the experiments. Gatan Digital Micrograph software was used for STEM image, HRTEM and SAED.

**Proteomic analysis**

**1. TMT Labeling of Peptides:** Osteochondral interface tissues (n=3) from normal and OA-E were prepared by cryo-sectioning. After being washed with PBS, the tissues were grinding through pestles under liquid nitrogen condition until it became power, cells were lysed in cold modified RIPA buffer (50 mm Tris-HCl, pH 7.5, 1 mm EDTA, 150 mm NaCl, 1% N-octylglycoside, 0.1% sodium deoxycholate, complete protease inhibitor mixture) and incubated for 30 min on ice. After that, lysates were cleared by centrifugation. 200 μg protein samples from the tissues were counted by BCA assay. Each protein sample was taken, and the volume was made up to 100 μL with DB dissolution buffer (8 m Urea, 100 mm TEAB, pH 8.5). Trypsin and 100 mm TEAB buffer were added, and the sample was mixed and digested at 37 °C for 4 h. Then, trypsin and CaCl_2_ were added before digesting the sample overnight. Formic acid was mixed with the digested sample, pH adjusted to under 3 and centrifuged at 12000 g for 5 min at room temperature. The supernatant was slowly loaded to the C18 desalting column, washed with washing buffer (0.1% formic acid, 3% acetonitrile) three times, then eluted with an elution buffer of 0.1% formic acid, 70% acetonitrile. The eluents of each sample were collected and lyophilized. 100 μL of 0.1 m TEAB buffer was added to reconstitute, and 41 μL of acetonitrile-dissolved TMT labeling reagent was added, and the sample was mixed with shaking for 2 h at room temperature. The reaction was stopped by adding 8% ammonia. All labeling samples were mixed with equal volume, desalted, and lyophilized.

**2. Separation of Fractions and LC-MS/MS Analysis:** Mobile phases A (2% acetonitrile) and B (98% acetonitrile) were used to develop a gradient elution. The lyophilized sample was dissolved in 2% acetonitrile solution and centrifuged at 12 000 g for 10 min. Then, the sample solution was fractionated with a C18 column (Waters BEH C18 4.6 × 250 mm, 5 μm) on a Rigol L3000 HPLC system. Finally, the collected fractions were lyophilized and dissolved in 0.1% formic acid. Shotgun proteomics analyses were used for transition library construction using an EASY-nLC 1200 UHPLC system (Thermo Fisher) equipped with a Q Exactive HF mass spectrometer (Thermo Fisher) operating in the data-dependent acquisition mode. Peptides were separated in a home-made analytical column and then analyzed with the Q Exactive HF mass spectrometer (Thermo Fisher).

**3. Data Analysis:** Following TMT labeling and LC-MS/MS analysis, the raw peptide intensity data were normalized using the internal reference scaling method to correct for technical variation and ensure quantitative comparability across all samples prior to statistical analysis. Proteome Discoverer 2.4 (PD 2.4, Thermo) was used to search for the resulting spectra with the following parameters: mass tolerance for precursor ions was 10 ppm; mass tolerance for product ion was 0.02 Da; Carbamidomethyl was specified as fixed modifications; Oxidation of methionine (M) and TMT plex were specified as a dynamic modification. Acetylation, TMT plex, Met-loss, and Met-loss + Acetyl were specified as N-Terminal modification in PD 2.4. A maximum of two missed cleavage sites were allowed. The identified peptide spectrum matches and protein were retained and used with no more than 1.0% FDR. A T-test was used to analyze the protein quantification results. The proteins whose quantitation was significantly different between experimental and control groups (P < 0.05 and |log_2_FC| > 1.5 [fold change, FC]) were defined as differentially expressed proteins.

**FEA**

Finite element analysis (FEA, Abaqus/CAE 6.13) was used to simulate the model. The model was divided into 100 layers for discretization with assuming the modulus in each layer was identical. The interface model was meshed using Plane 8-node biquadratic shell elements and assigned linear elastic material properties. The number of elements and nodes were 10000 and 30401, respectively. The elastic modulus of each layer in the simulation was taken from the model function we set and the Poisson’s ratio was taken 0.49 for disc, 0.46 for cartilage and 0.2 for SB. Meanwhile, the load for compression displacement loading is set as 50μm. The von Mises stress was selected to measure the mechanical behavior of the interface region.

**Cartilage plugs *in* *vitro* loading culture**

Healthy condylar samples were dissected form head of porcine (male, aged 7~8 months, n=3), which were obtained from a local abattoir. The cartilage sampling was performed following a standardized protocol. All procedures, including the harvesting, sectioning, medium addition, and placement of the mandibular condylar cartilage explants into the loading dishes, were performed under aseptic conditions within a laminar flow hood. The cartilage explants were collected exclusively from the superior aspect of the condylar apex. The harvested explants were then measured and examined, and only those with consistent morphological characteristics, such as cartilage thickness and texture across the cross-section, were selected for subsequent loading experiments. Cartilage samples were dissected into small plugs (Standardization of Plug Sizes: 1cm×1cm) including cartilage interface and SB and washed with sterile PBS for next experiment. Cartilage plugs were then cultured and under loading by Flexcell FX5000 Compression system. The culture medium (DMEM F12 medium with 10% fetal bovine serum and 1% penicillin-streptomycin) was replenished every 48 hours to ensure a continuous supply of nutrients, and other culture conditions 37℃, 5%CO₂ levels was set. Before experiment, loading conditions was calibrated, all mechanical loading experiments were performed using unconfined compression. In Flexcell FX5000 compression system, the loading mechanical stress was set by 0.5MPa (physiological stress), 1.0MPa (mild overloading stress) and 1.5MPa (severe overloading stress), frequency of loading was set by 1Hz, respectively. After continuous loading culture in 1, 3 and 5 days, cartilage plugs were collected and for histological and micro-nano phenotypes were characterized. The blank control group in the preliminary experiments had already ruled out any effects from the culture conditions.

**In vitro chondrocyte co-culture with fibronectin**

The fresh adult porcine condylar cartilage was obtained. The superficial fibrous layer was removed under a stereomicroscope, and the remaining cartilage tissue was digested with type II collagenase to extract primary chondrocytes. The cells were passaged to generation P3. These P3 cells were then seeded into 6-well plates and cultured in DMEM/F12 medium supplemented with 5% BSA and 1% penicillin-streptomycin. After cell adhesion, they were treated with 2mg/mL and 5 mg/mL of human recombinant fibronectin protein (MCE HY-P3160) for co-culture. After 7 days of culture, Alizarin Red staining and transmission electron microscopy (TEM) were performed.

**Statistical analysis**

Preprocessing procedures for data were performed prior to statistical analysis, including transformation of non-normal data, normalization of variables to a common scale, and outlier detection using the interquartile range method. Experimental results were expressed as mean ± SD. The sample size (n) for each statistical analysis refers to the number of independent biological replicates, with specific values indicated in the figure legends (e.g., n = 3 represents three independent samples). Statistical differences were assessed using one-way analysis of variance (ANOVA) followed by Tukey's post-hoc test for multiple comparisons, and two-tailed Student's t-test for pairwise comparisons. The significance level (alpha value) was set at 0.05. Prior to analysis, the assumptions for ANOVA (normality via Shapiro–Wilk test and homogeneity of variance via Levene's test) were verified, and data meeting these assumptions were included. Statistical significance was defined as *p < 0.05, **p < 0.01, ***p < 0.001, ****p < 0.0001, and ns represents no significant difference. All statistical analyses were performed using GraphPad Prism version 8.0 (GraphPad Software, Inc., CA, USA).

**Supplementary Text**


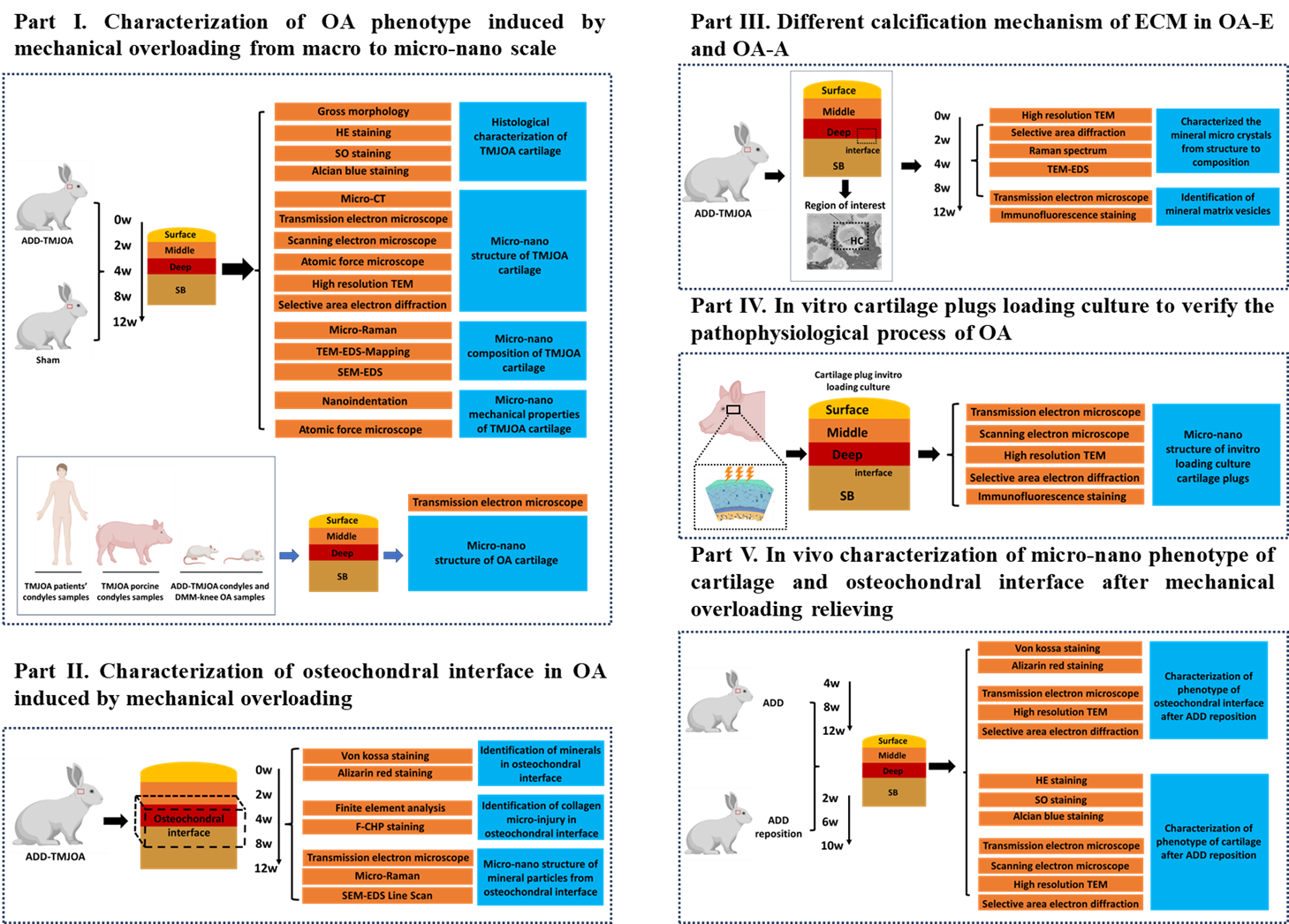


**Figure. S1. Flow chart illustrating the sequence of experiments performed in this study.**


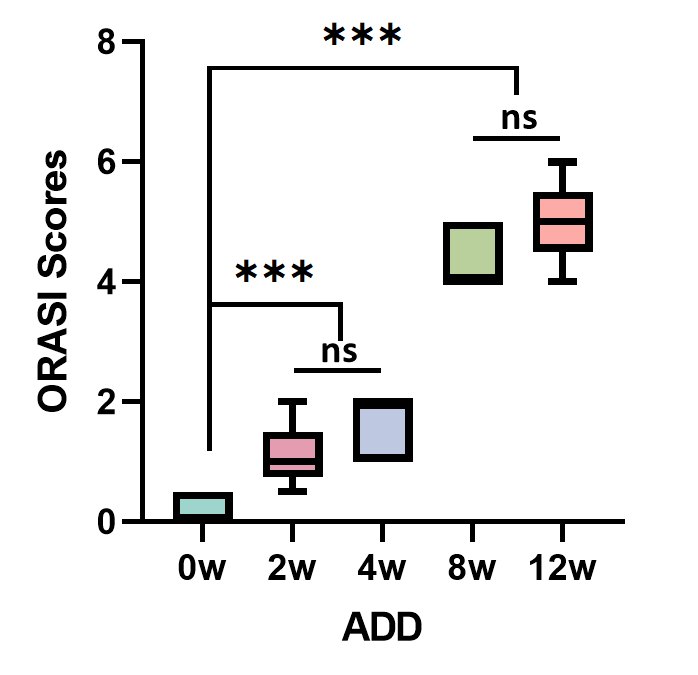


**Figure S2. Rabbit TMJOA Stages estimated by ORASI score system using HE staining image.** Normal was defined as add0w, OA-early (OA-E) was defined as add2w and add4w, and OA-advanced (OA-A) defined as add8w and add12w, n=3. ns, no significance; ***, P＜0.001.


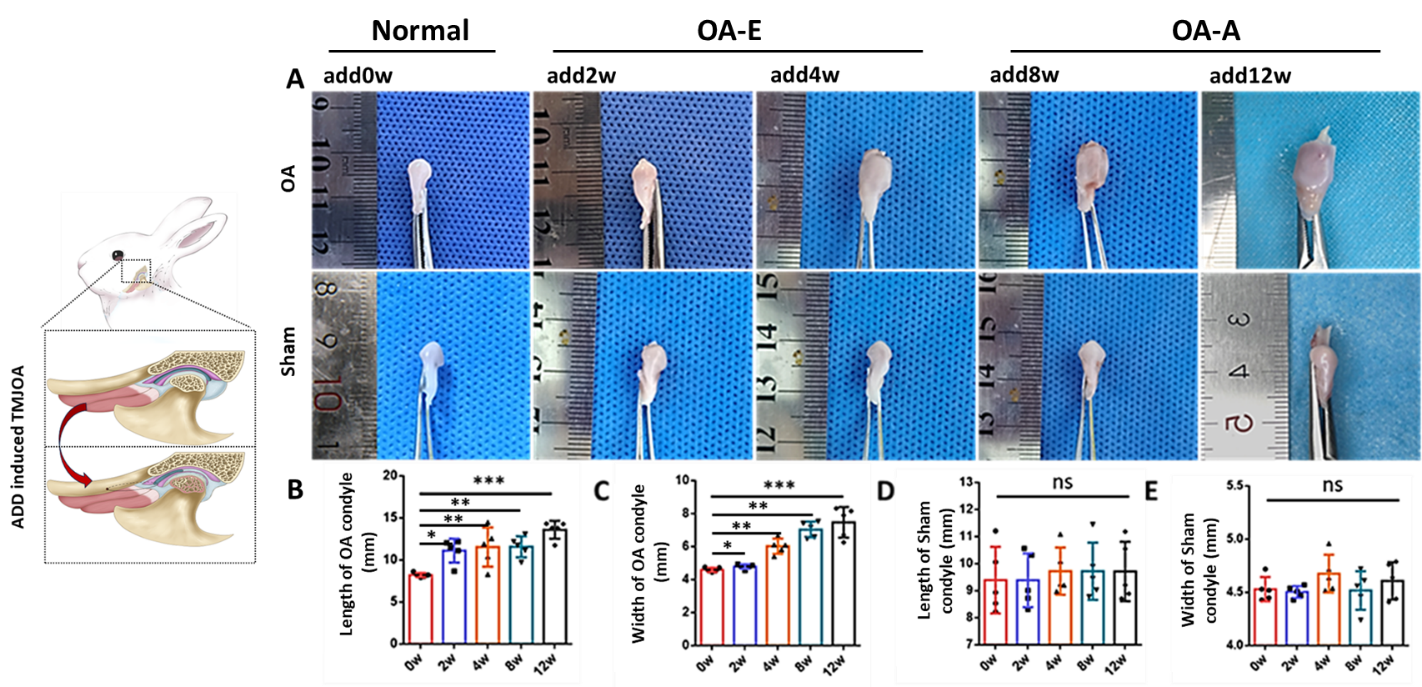


**Figure. S3. Gross morphology of TMJOA condyle (Rabbit TMJOA models).** A. Macroscopic images of condyle in ADD induced TMJOA and sham group; B-C. Length and width of condyle in OA (n=5). D-E. Length and width of condyle in sham group (n=5). All data were presented as mean ± standard deviation. Statistical analyses are performed by paired t-test and one-way ANOVA. ns, no significance; *, P < 0.05; **, P < 0.01; ***, P＜0.001.


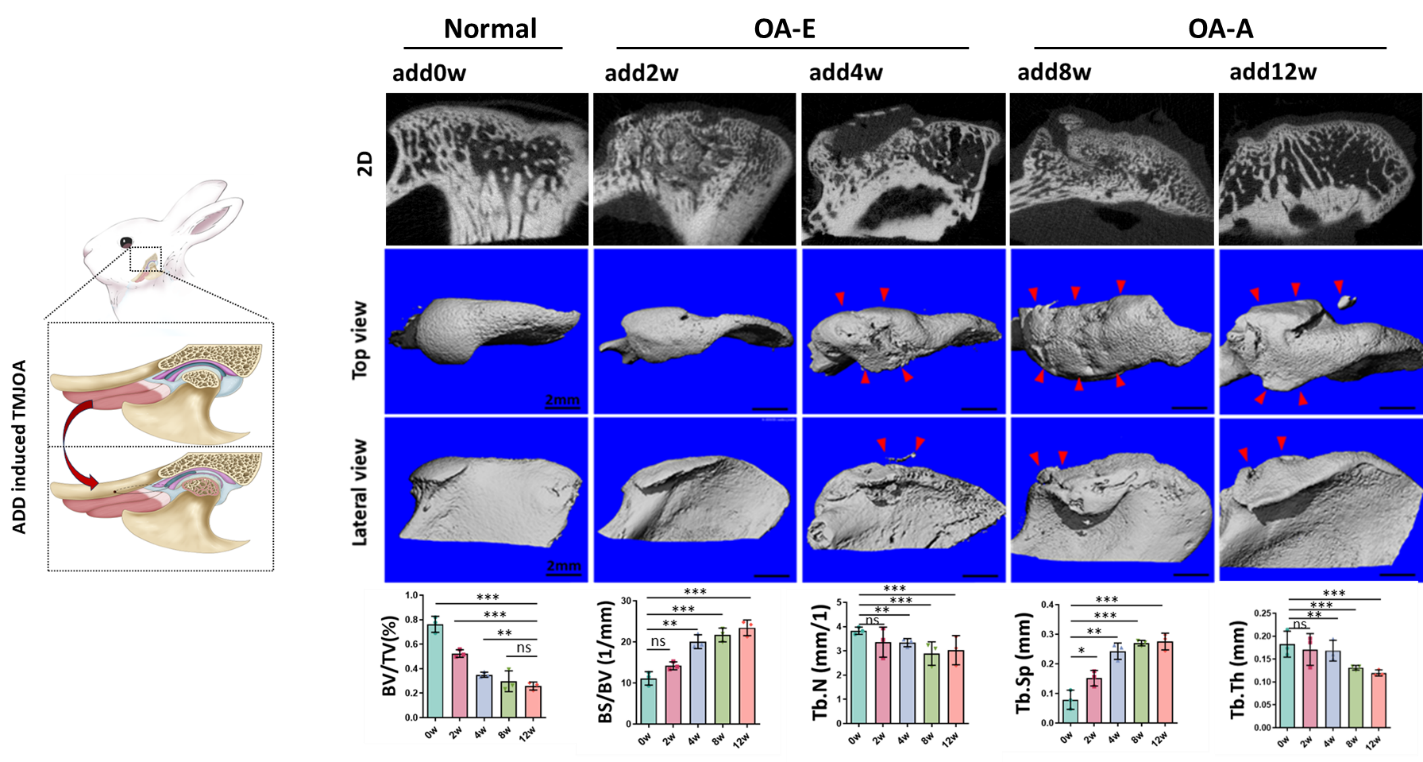


**Figure. S4. Representative micro-CT images and parameters of subchondral bone remodeling of the TMJ condylar head from the TMJOA side with pathological cartilage mineralization (red arrows) (Rabbit TMJOA models).** Scale bars, 2 mm. All data were presented as mean ± standard deviation, n=3. Statistical analyses are performed by paired t-test. ns, no significance; *, P < 0.05; **, P < 0.01; ***, P＜0.001.


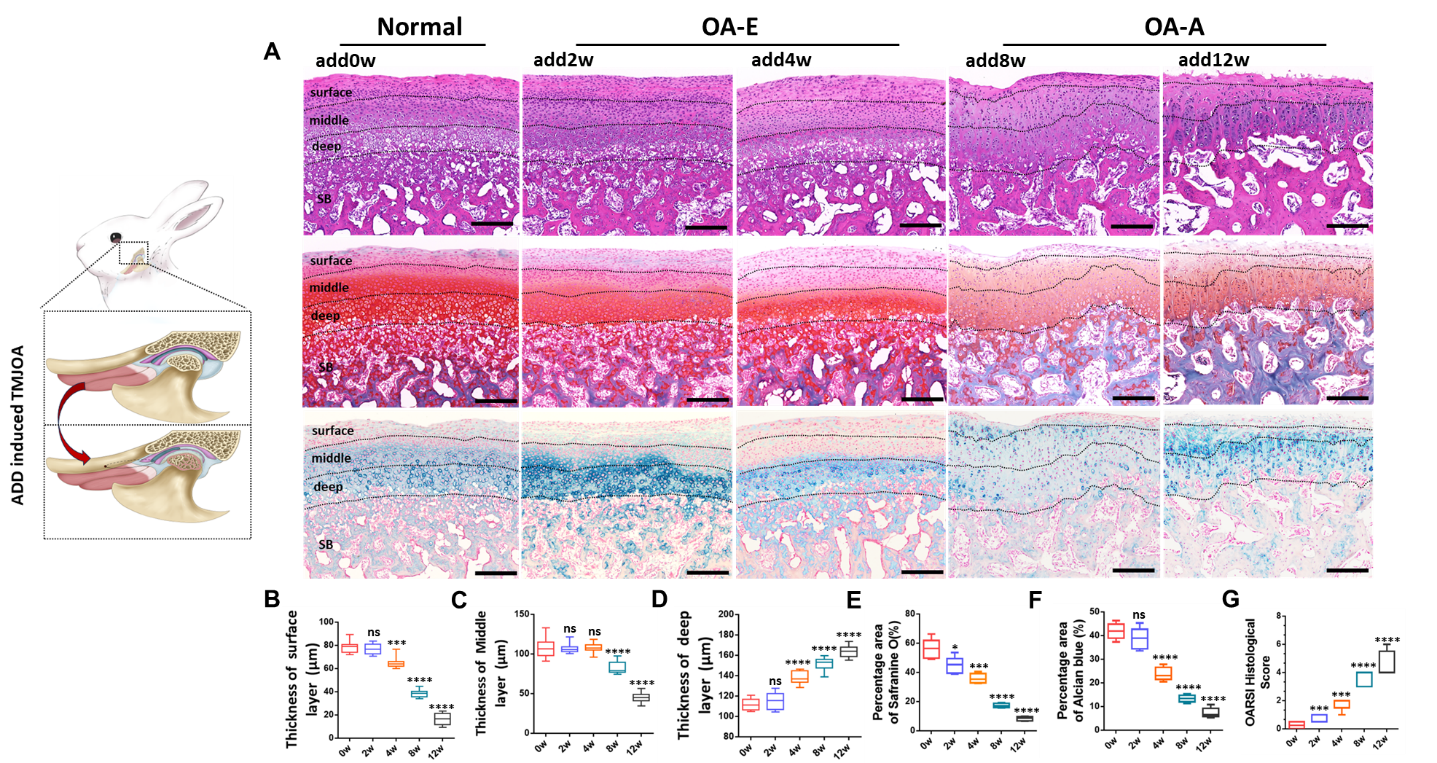


**Figure. S5. Histological features of condylar cartilage in ADD induced TMJOA (Rabbit TMJOA models).** A. HE, SO and Alcian blue staining images, respectively; B-D. Quantitative thickness of three condylar cartilage layers (n=3); E-F. Percentage of GAGs positive area quantification by SO and Alcian blue staining (n=3); G. The Osteoarthritis Research Society International (OARSI) histological score of the osteoarthritis (n=3). All data were presented as mean ± standard deviation. Statistical analyses are performed by paired t-test. ns, no significance; *, P < 0.05; ***, P < 0.01; ****, P＜0.001.


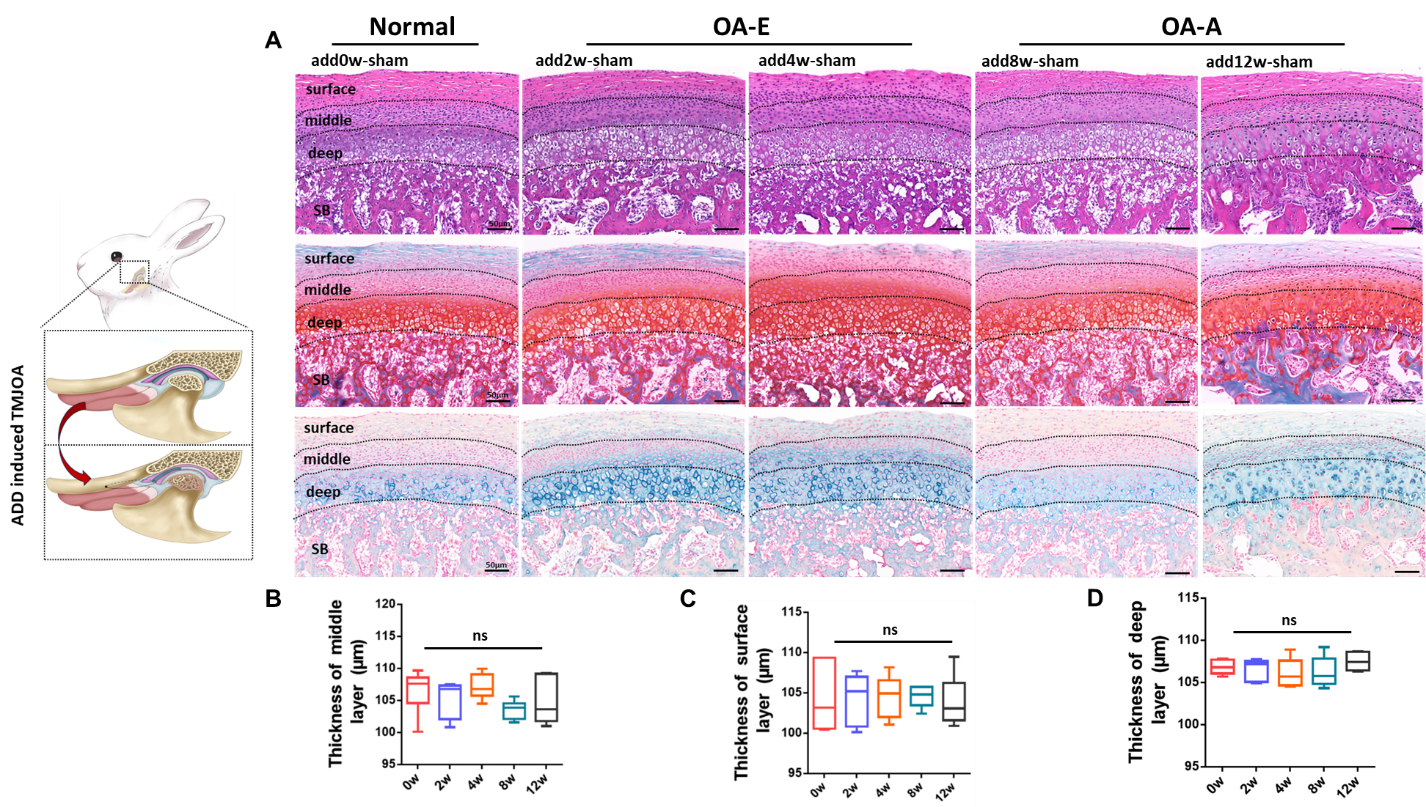


**Figure. S6. Histological features of condylar cartilage in TMJOA sham group (Rabbit TMJOA models).** A. HE, SO and Alcian blue staining images, respectively; B-D. Quantitative thickness of three condylar cartilage layers (n=3). All data were presented as mean ± standard deviation. Statistical analyses are performed by one-way ANOVA. ns, no significance.


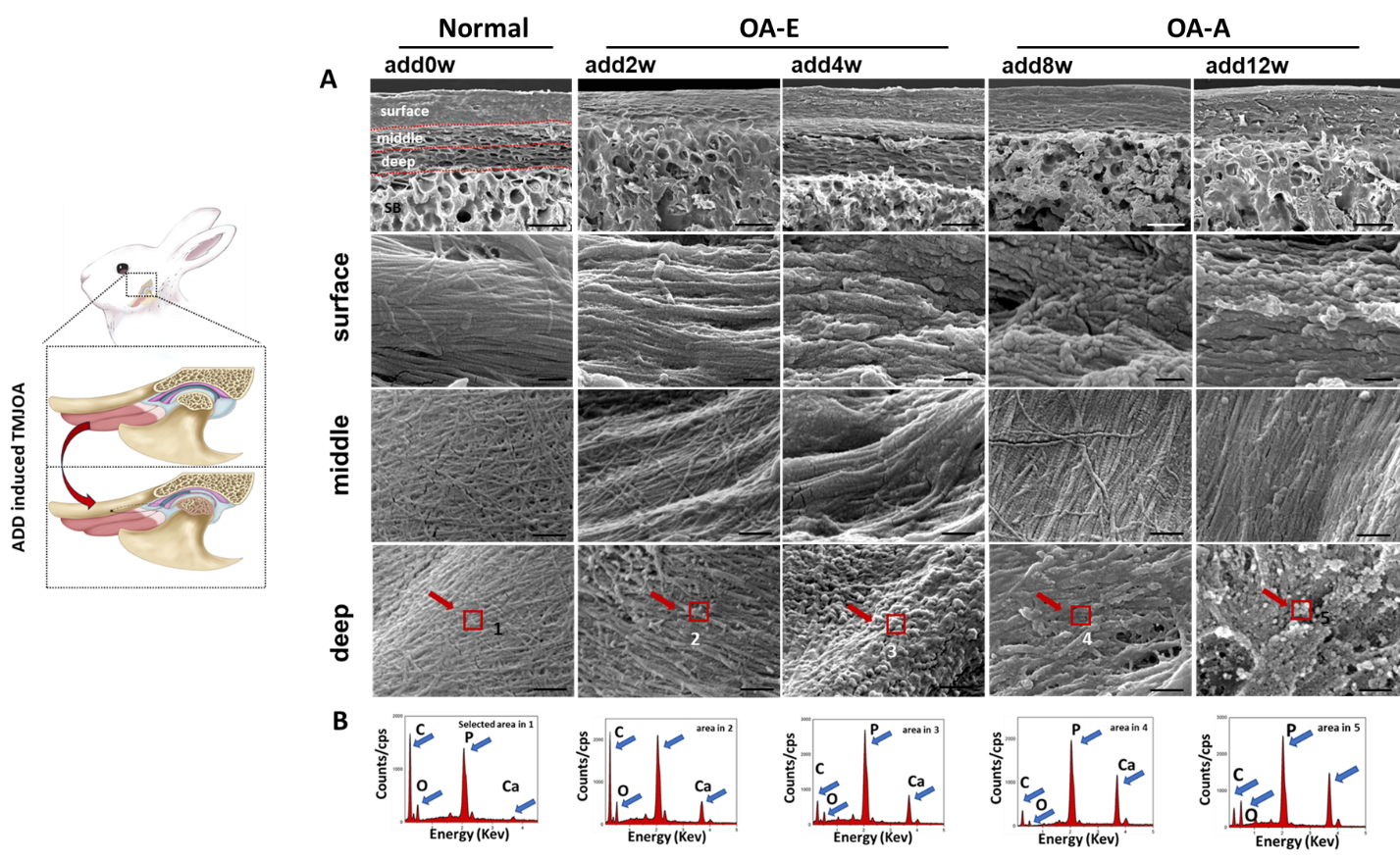


**Fig. S7. Nano-micro structure and composition characteristics of cartilage during OA development (Rabbit TMJOA models).** A. SEM images of TMJOA cartilage during OA progression; scale bar, low magnification image was 100μm, high magnification image was 200nm; B. Elemental analysis of the calcified particulates indicated by the red arrow area of deep layer in A.


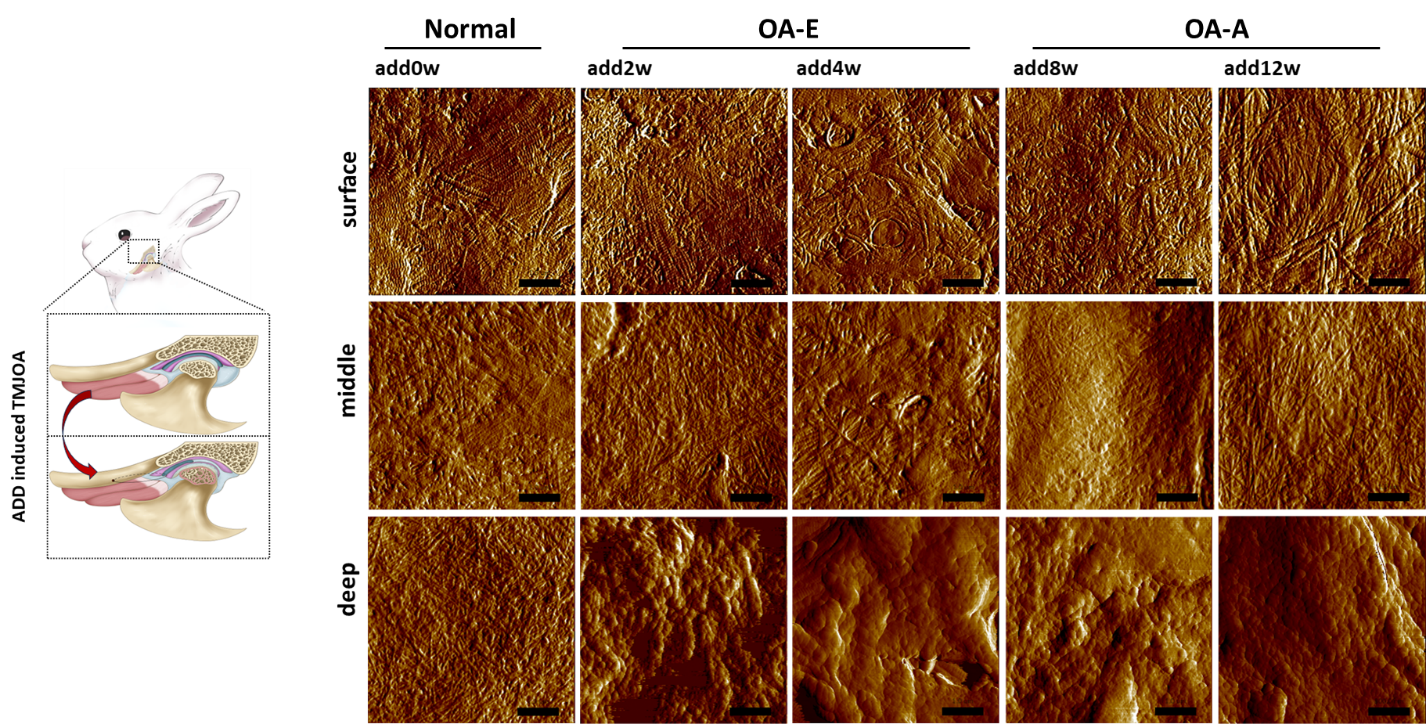


**Fig. S8. Nano-micro structure characteristics of cartilage during OA development characterized by AFM (Rabbit TMJOA models).** Scale bar, 1μm.


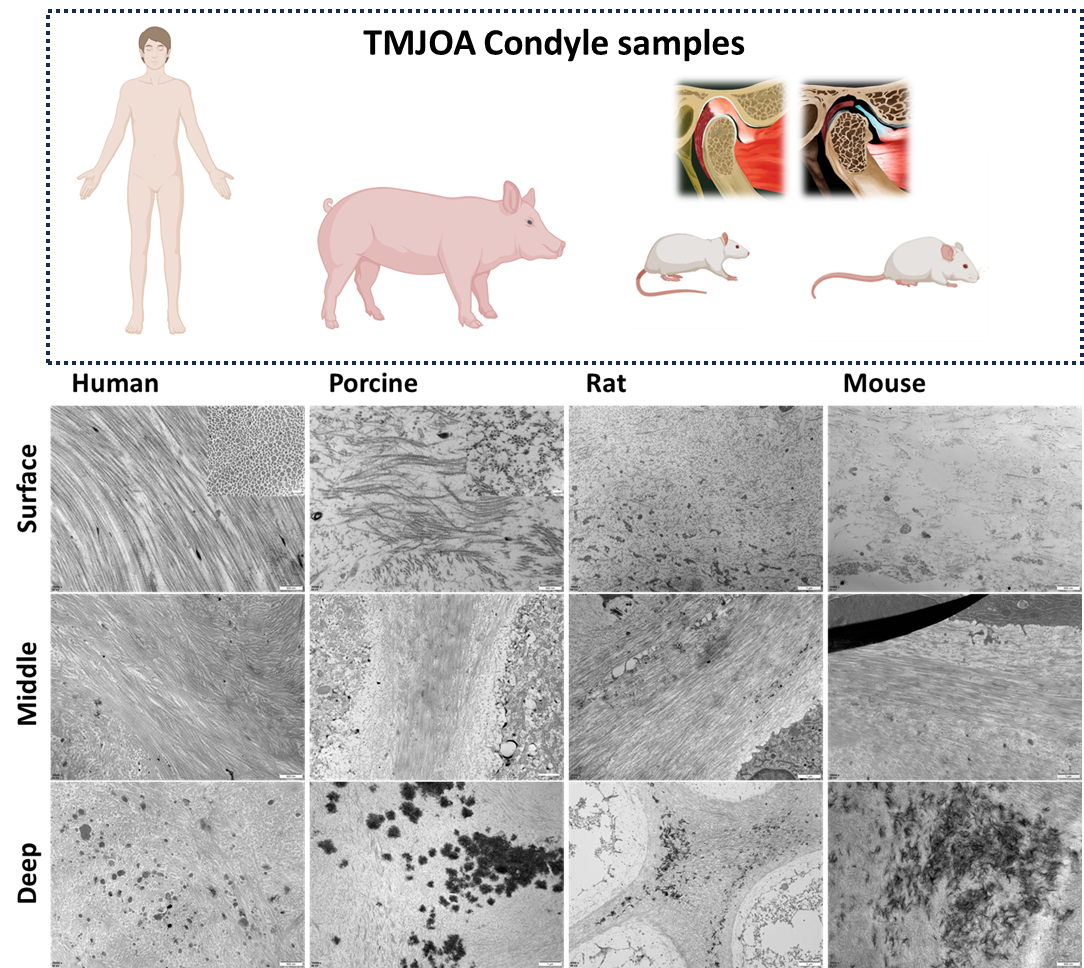


**Fig. S9. TEM images of rat and mouse TMJOA cartilage induced by ADD and human, porcine TMJOA cartilage samples.** Validation of nano-micro structural phenotypes in porcine, human, rat and mouse TMJ cartilage. Human samples (n=3); Porcine samples (n=5); Rat samples (n=5); Mouse samples (n=5).


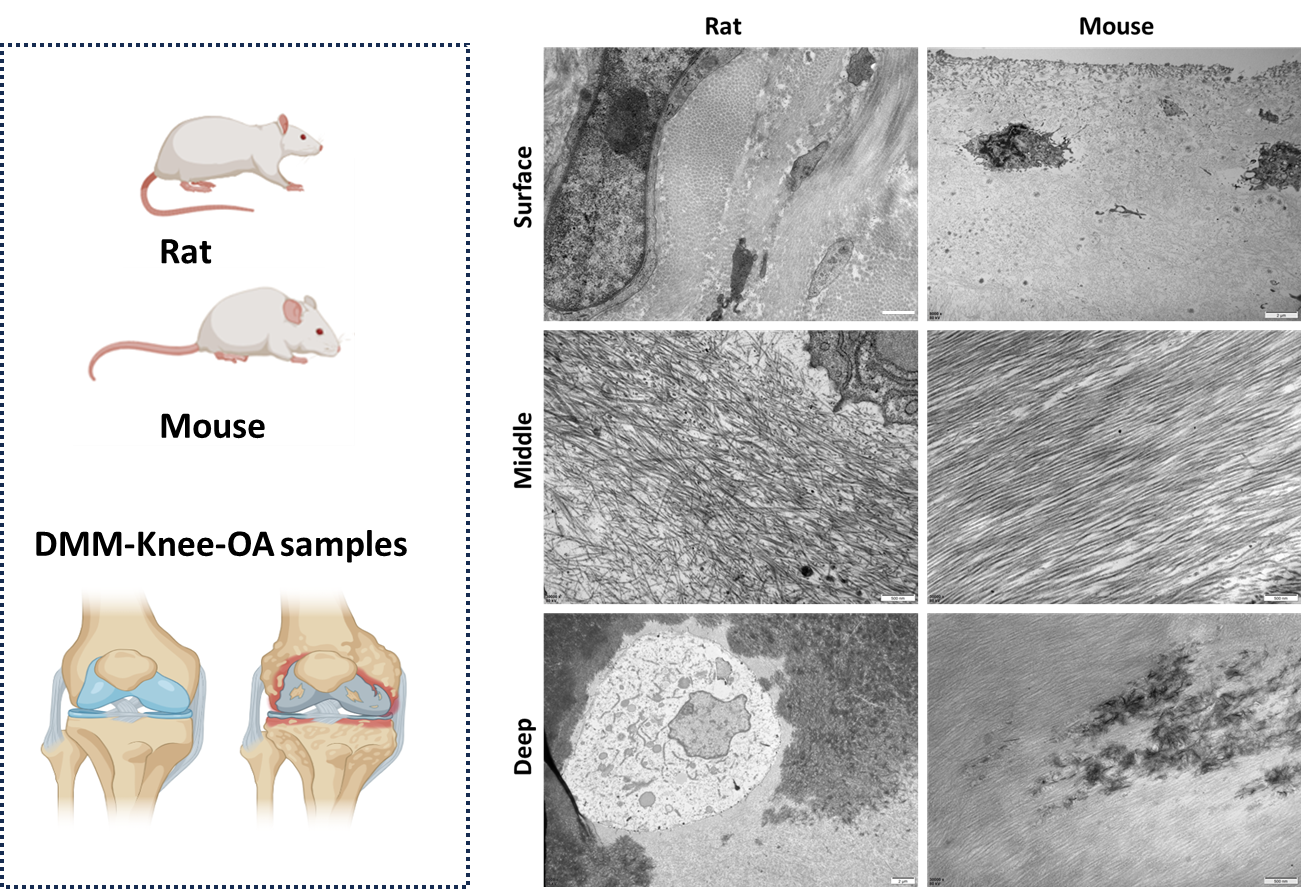


**Fig. S10. TEM images of rat and mouse knee OA cartilage induced by DMM.** Validation of nano-micro structural phenotypes in rat and mouse knee cartilage. Rat samples (n=5); Mouse samples (n=5).


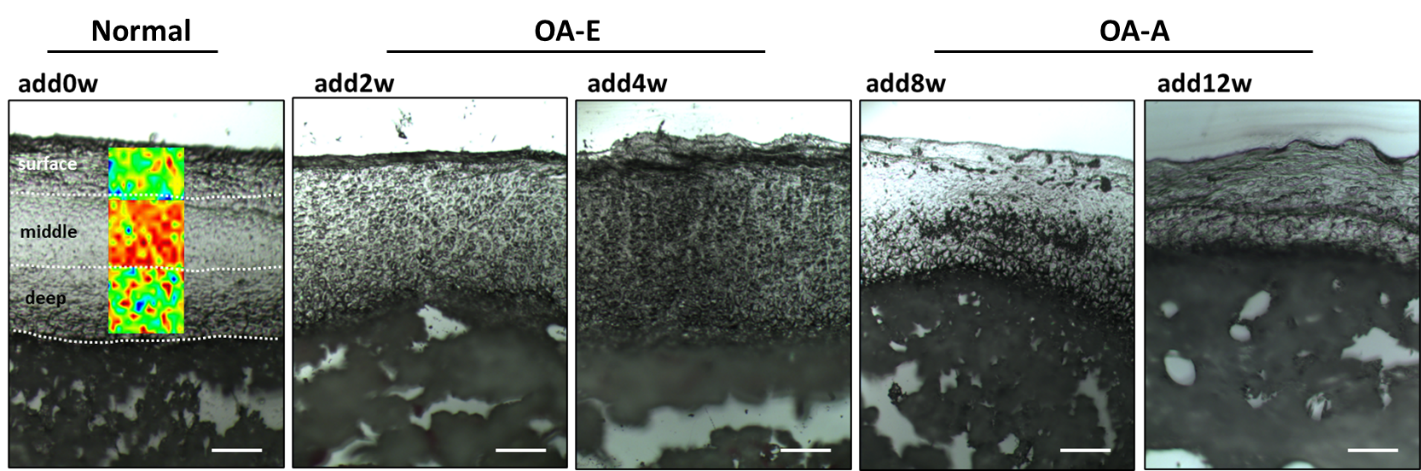


**Fig. S11. Method of micro-Raman mapping analysis and area selected of cartilage during OA development (Rabbit TMJOA models).** Scale bar, 100μm.


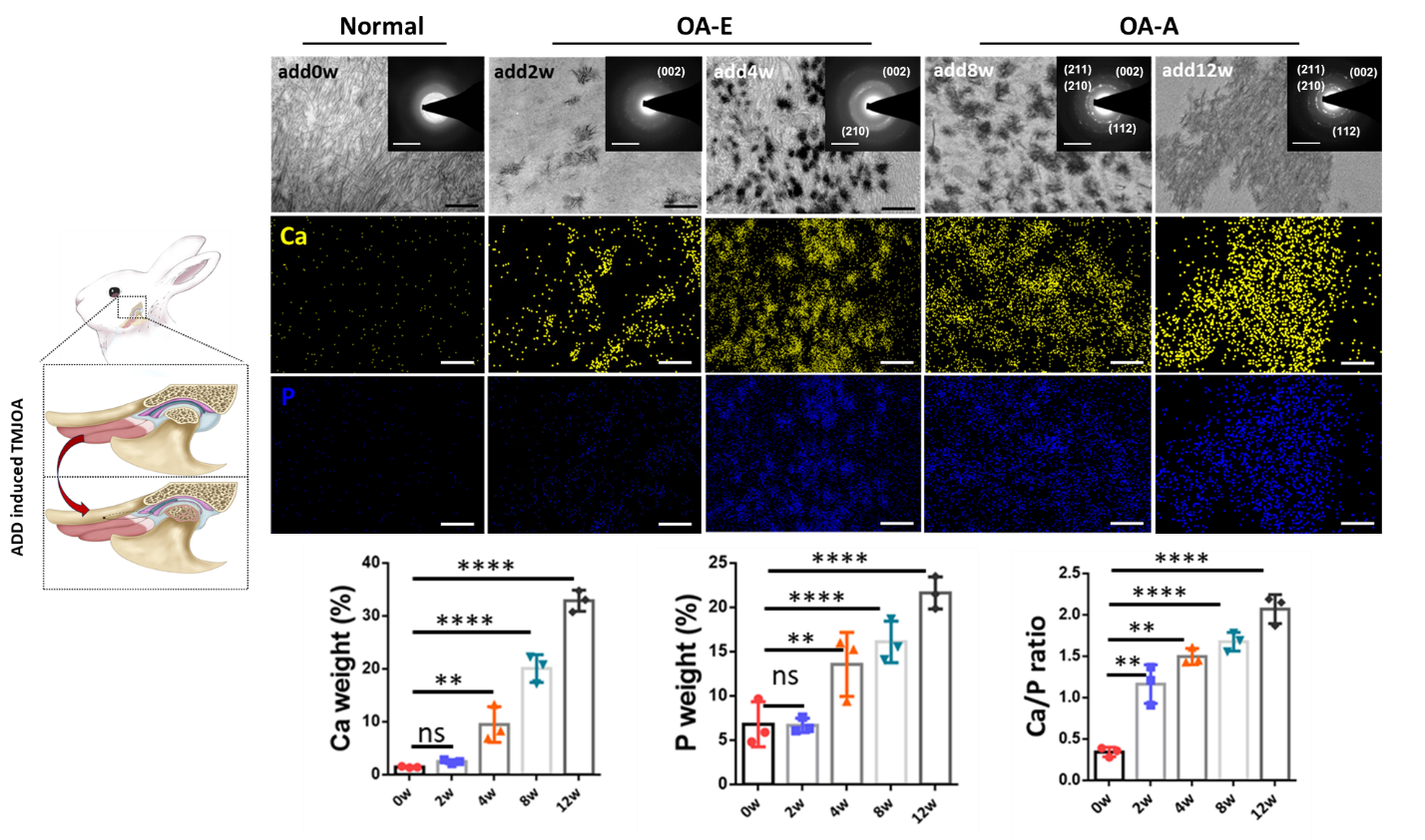


**Fig. S12. TEM and SAED images of typical crystals deposited in deep ECM (Rabbit TMJOA models).** Elemental mapping of the mineral crystals deposited in deep layer of OA cartilage by TEM-EDS and quantitative analysis of Ca and P distribution in the cartilage (n = 3). ns, no significance. **, P < 0.05; ****P < 0.01. Scale bar in TEM and TEM-EDS = 500 nm, in SAED = 5 1/nm.


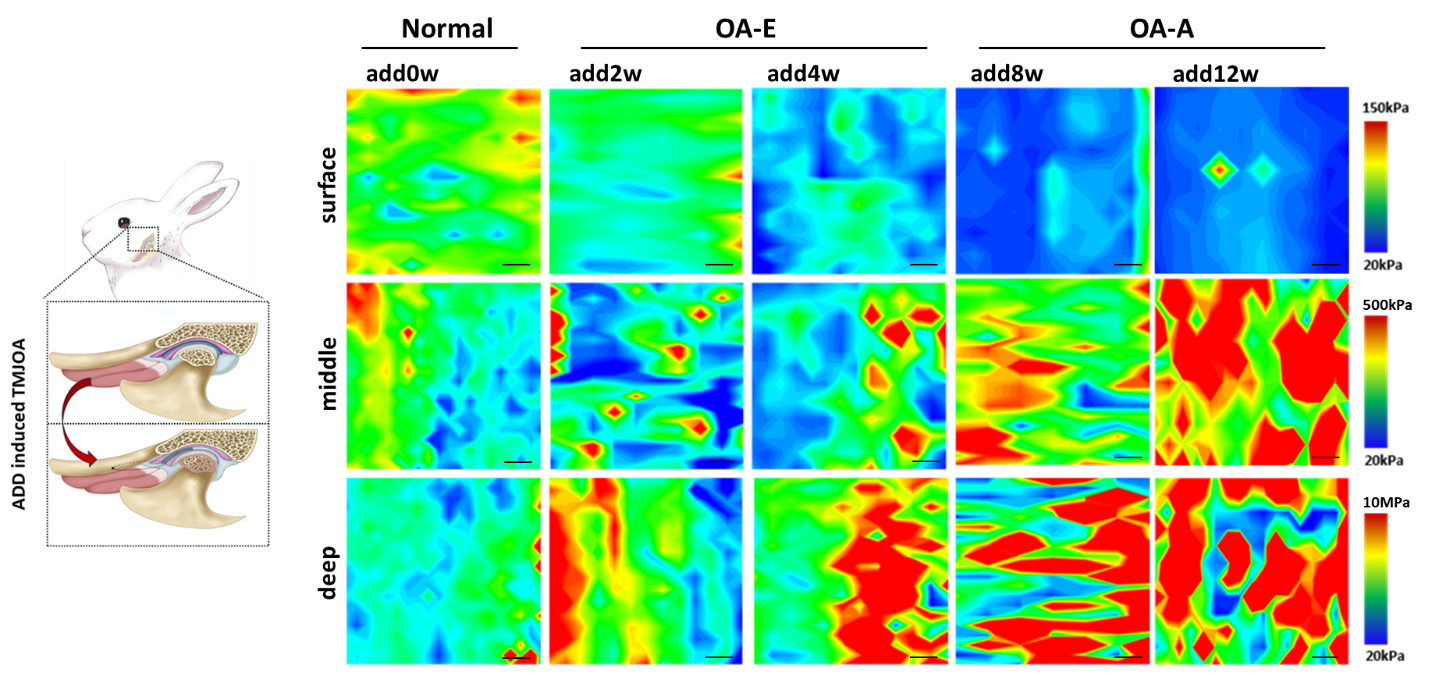


**Fig. S13. Height map of elastic modulus during OA development characterized by nanoindentation (Tip radius diameter ≈ 8.5μm) (Rabbit TMJOA models).** Scale bar 10 μm.


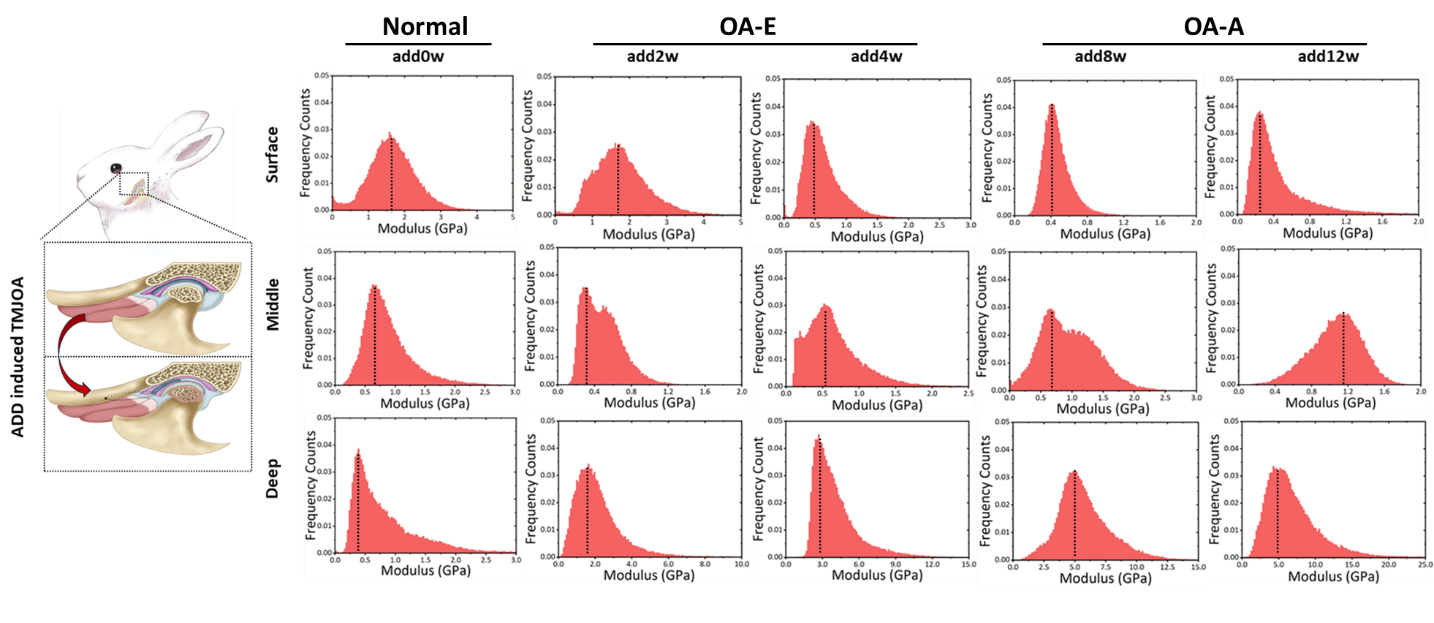


**Fig. S14. Elastic modulus distribution during OA progression obtained by AFM-indentation (Tip radius diameter ≈ 50nm) (Rabbit TMJOA models).**


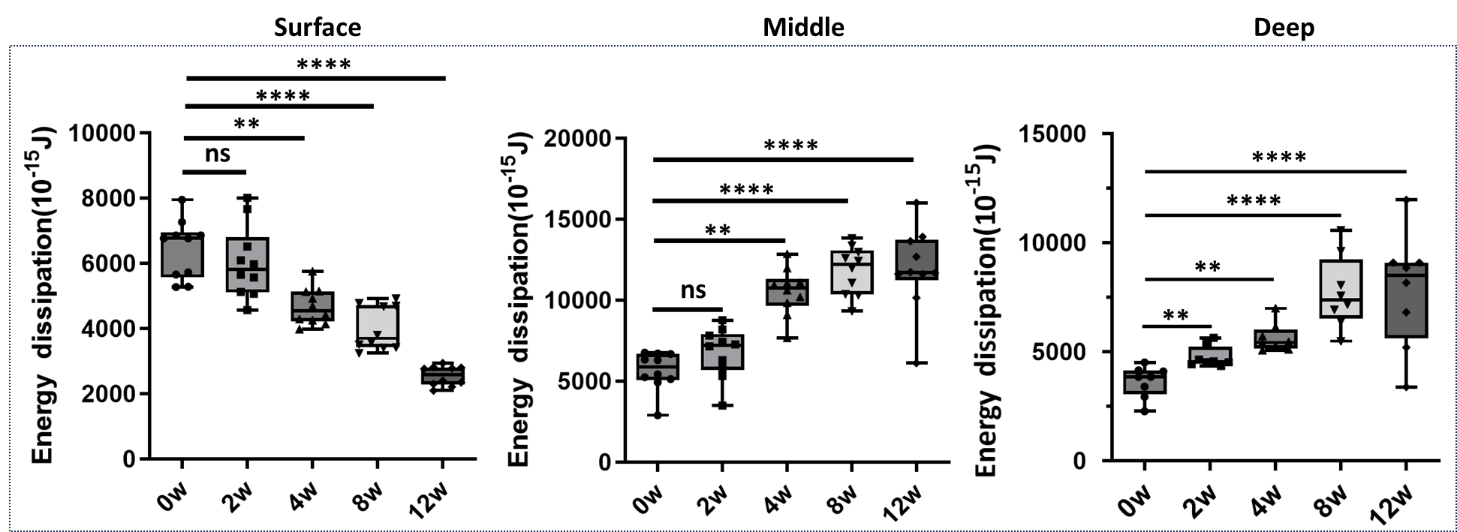


**Fig. S15. Energy dissipation was quantified and subjected to a statistical assessment during OA development (Rabbit TMJOA models).** The energy dissipation value is quantified by the area under the force-displacement hysteresis curve. (n = 10), ns, no significance. **, P < 0.05; ****P < 0.01.


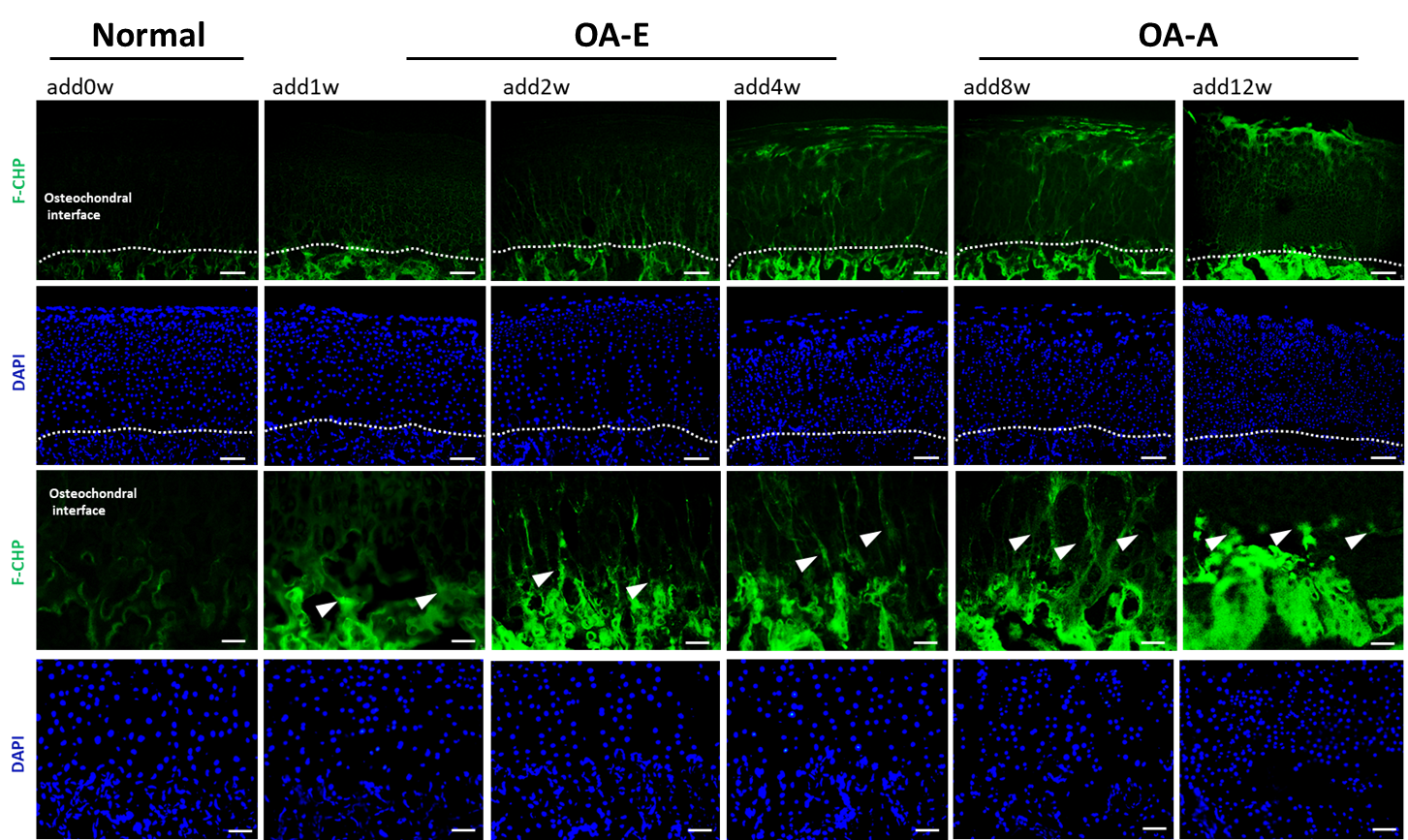


**Fig. S16. F-CHP staining for detection the location of collagen fibril trible-helix injury during OA development (Rabbit TMJOA models).** White arrows indicate collagen fibril injury. Upper row scale bar, 100 μm; Low row scale bar, 20 μm.


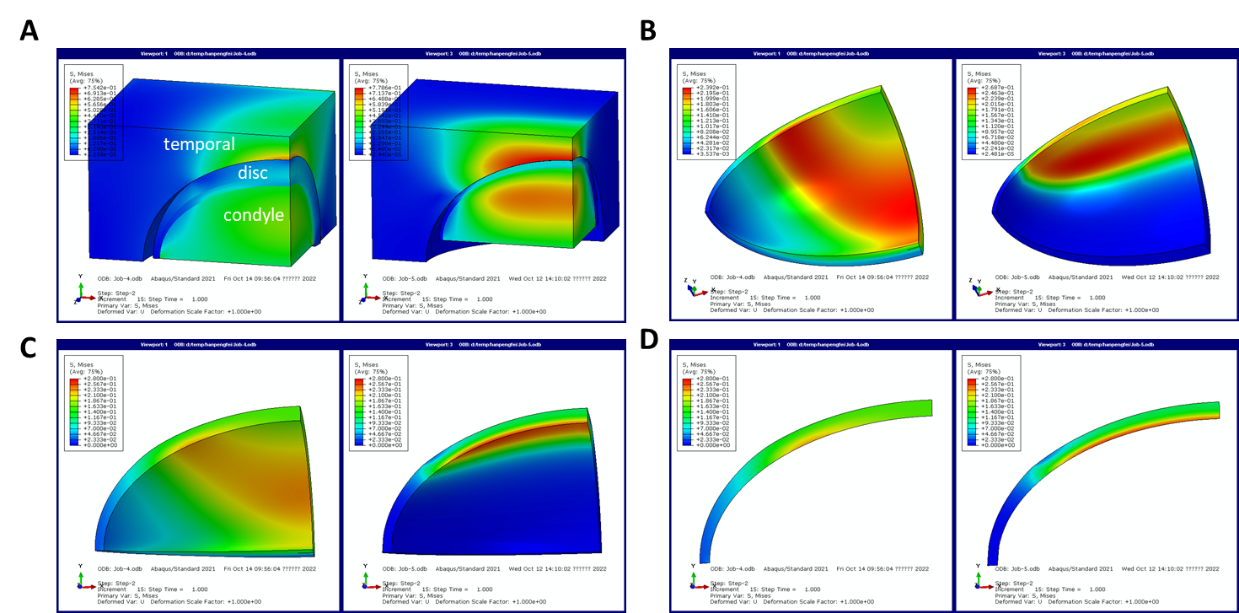


**Fig. S17. FEA of articular cartilage loading stress distribution of normal and ADD stages.** A. left side is normal state and the mechanical distributed homogeneously; Right side is ADD state and the stress distribution at the bone-cartilage interface becomes concentrated without the mechanical buffering provided by the articular disc. B-D. Left side represents normal state and right side represents ADD state.


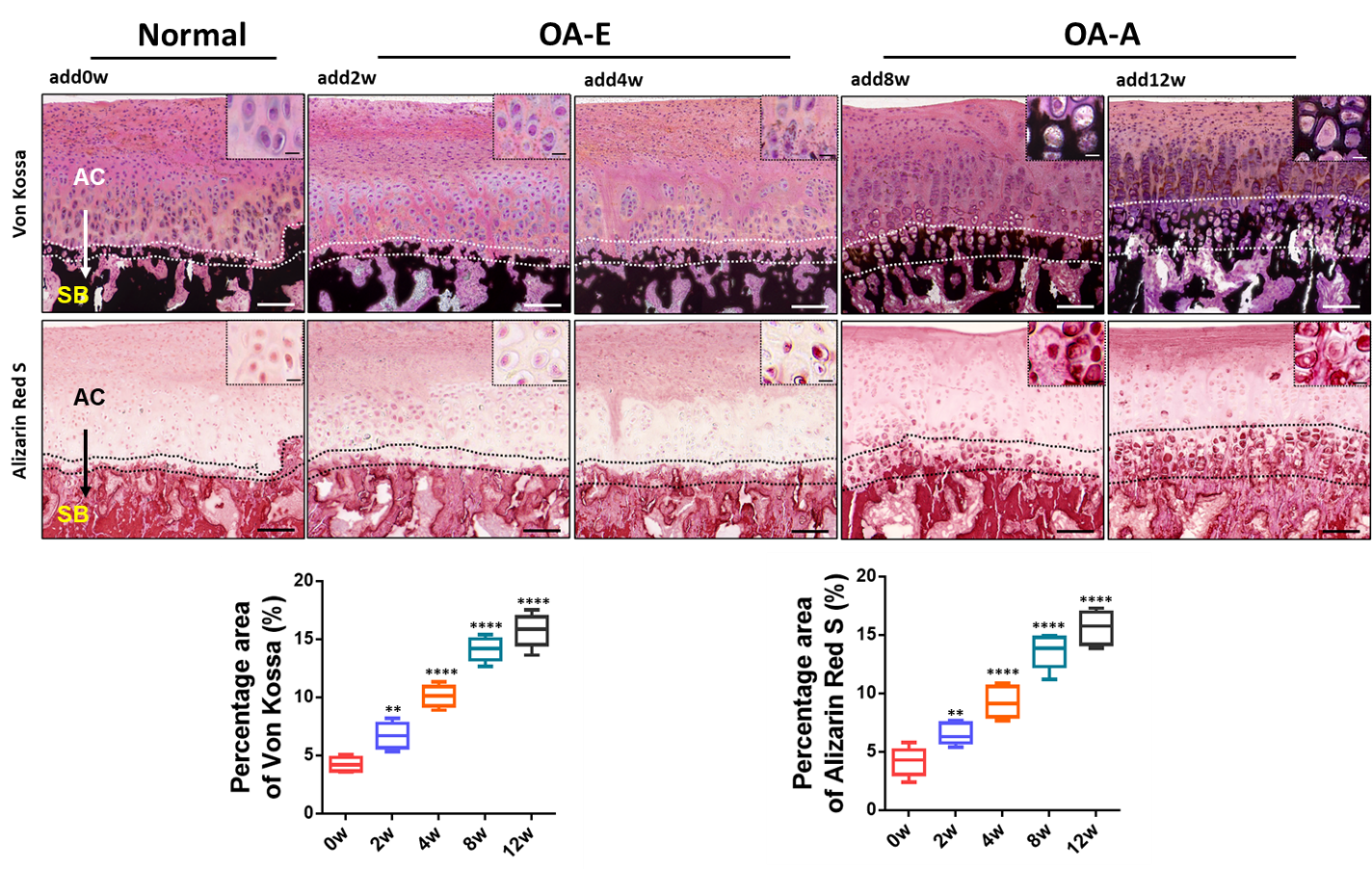


**Fig. S18. Von Kossa and Alizarin Red S staining and calcium distribution in osteochondral interface of OA cartilage during OA development (Rabbit TMJOA models).** AC, articular cartilage; SB, subchondral bone. Scale bar 50 μm. n = 3, **, P < 0.05; ****P < 0.01.


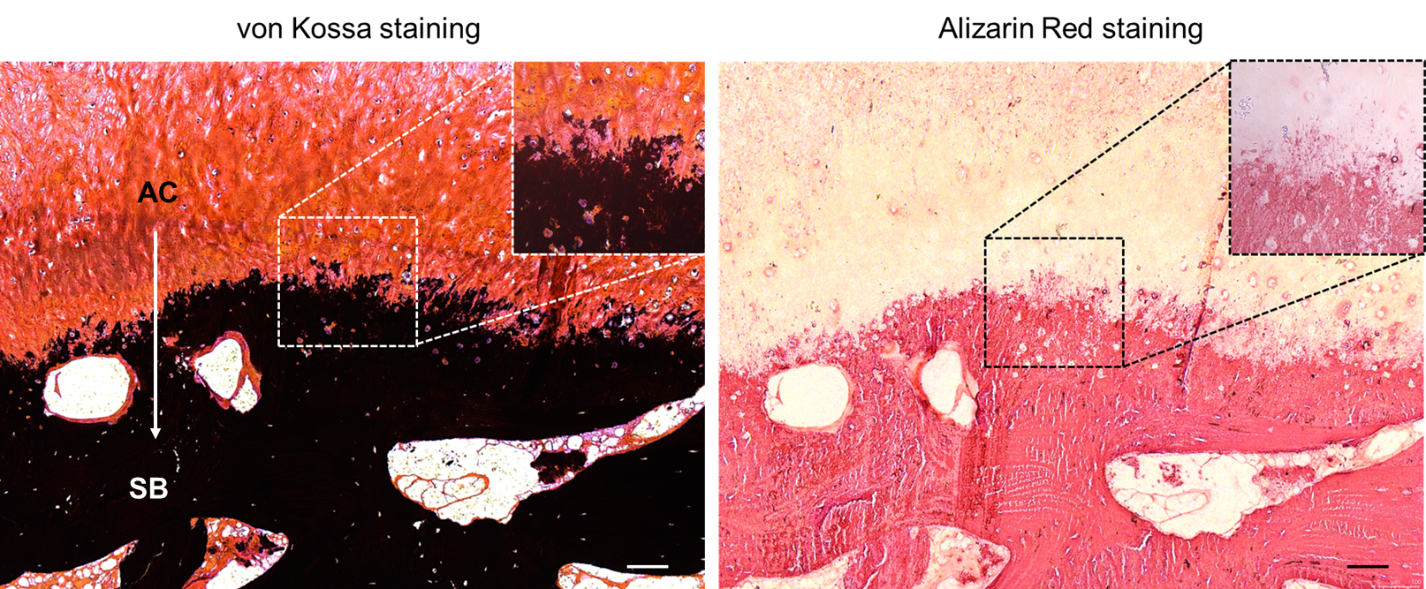


**Fig. S19. Von Kossa and Alizarin Red S staining of human TMJOA cartilage.** AC, articular cartilage; SB, subchondral bone. Scale bar 100μm.


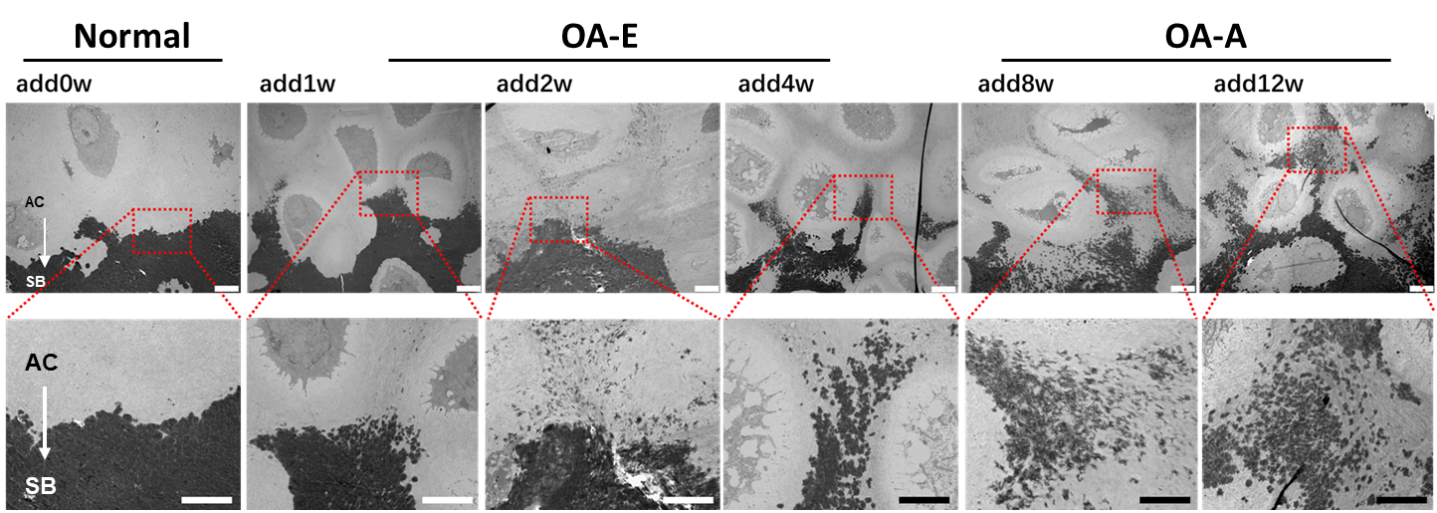


**Fig. S20. Representative TEM images of osteochondral interface during OA development (Rabbit TMJOA models).** AC, articular cartilage; SB, subchondral bone. Upper row scale bar was 10 μm and lower row scale bar was 1 μm.


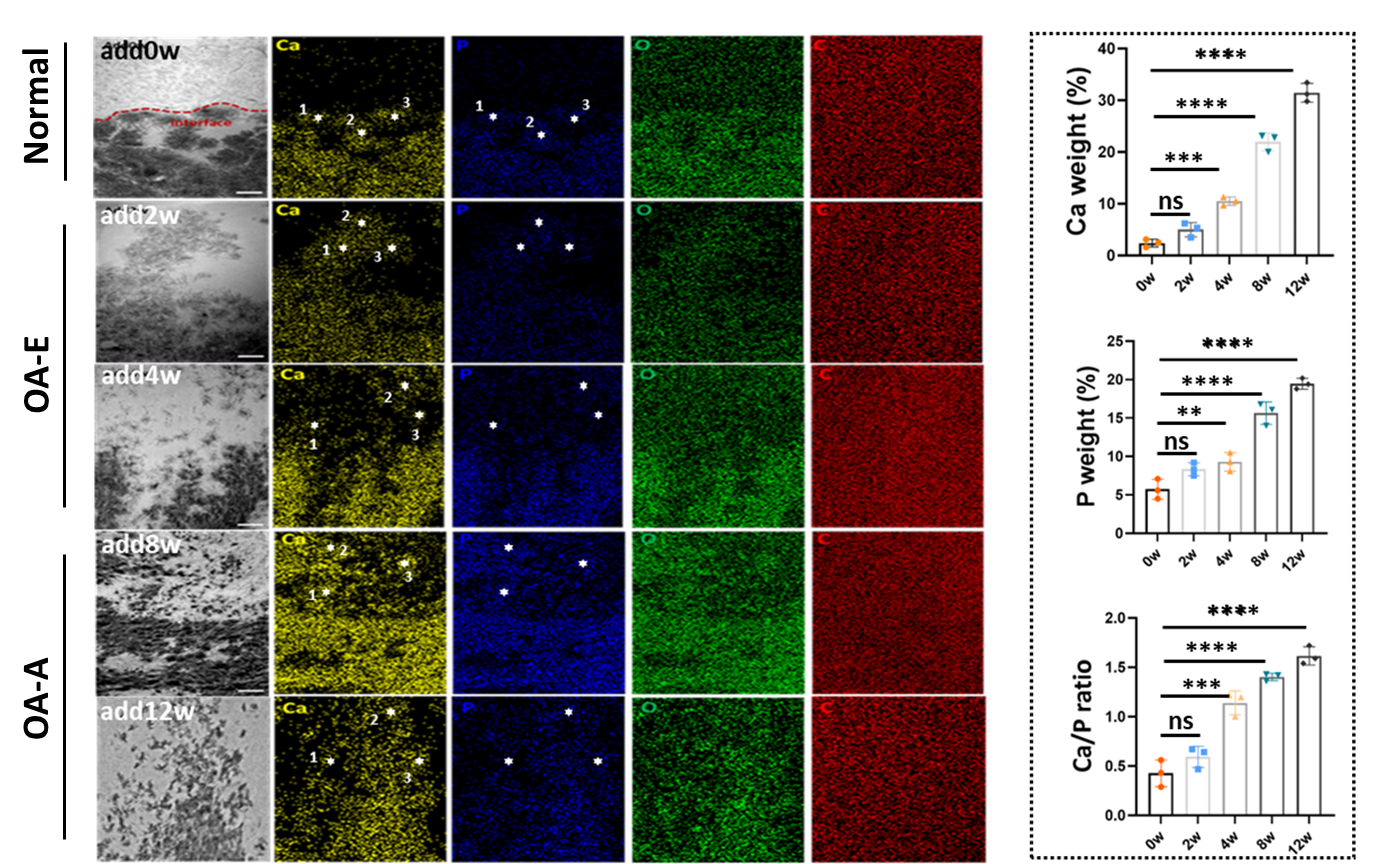


**Fig. S21. Elemental mapping of the osteochondral interface and quantitative analysis of Ca, P and Ca/P ratio (Rabbit TMJOA models).** Scale bar, 500 nm. n=3, ns, no significance. **, P < 0.05; ***P < 0.01; ****P < 0.001.


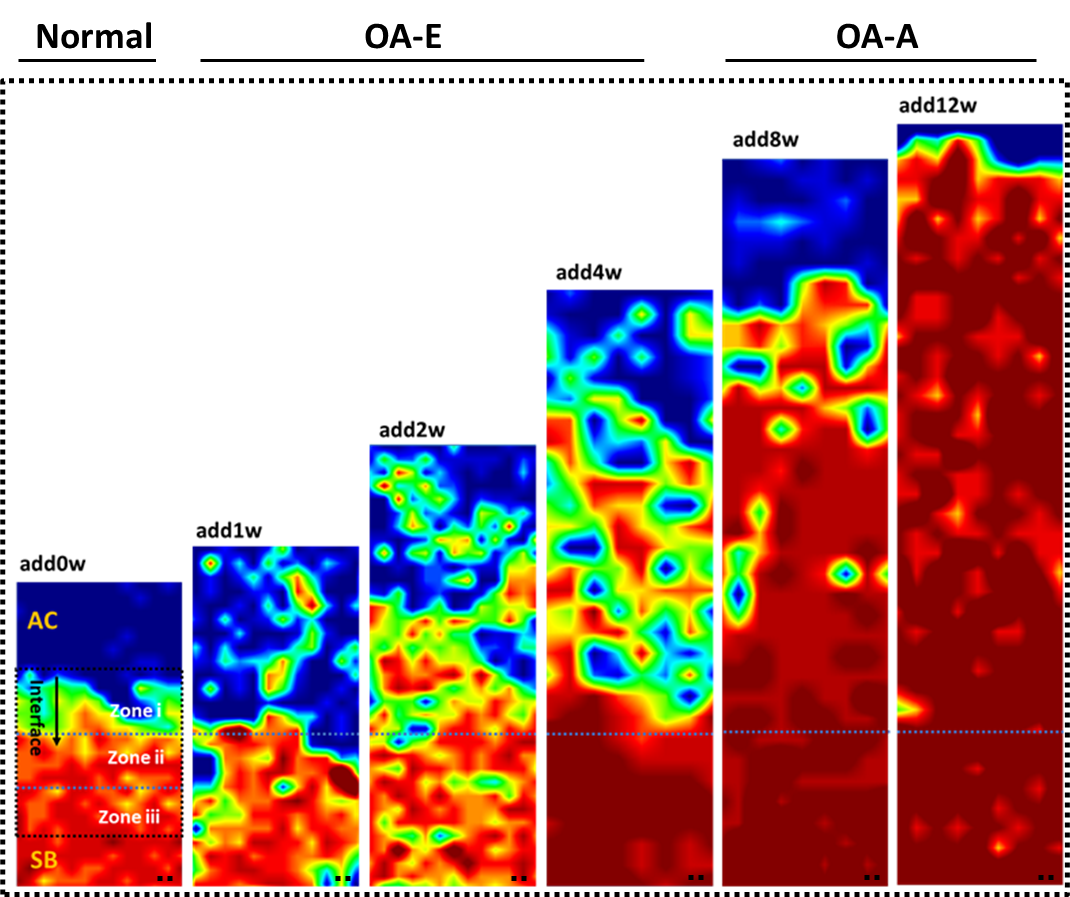


**Fig. S22. Micro-Raman mapping of osteochondral interface by 960 cm^-1^ (PO_4_^3-^) (Rabbit TMJOA models). AC, articular cartilage;** SB, subchondral bone. Scale bar, 5μm.


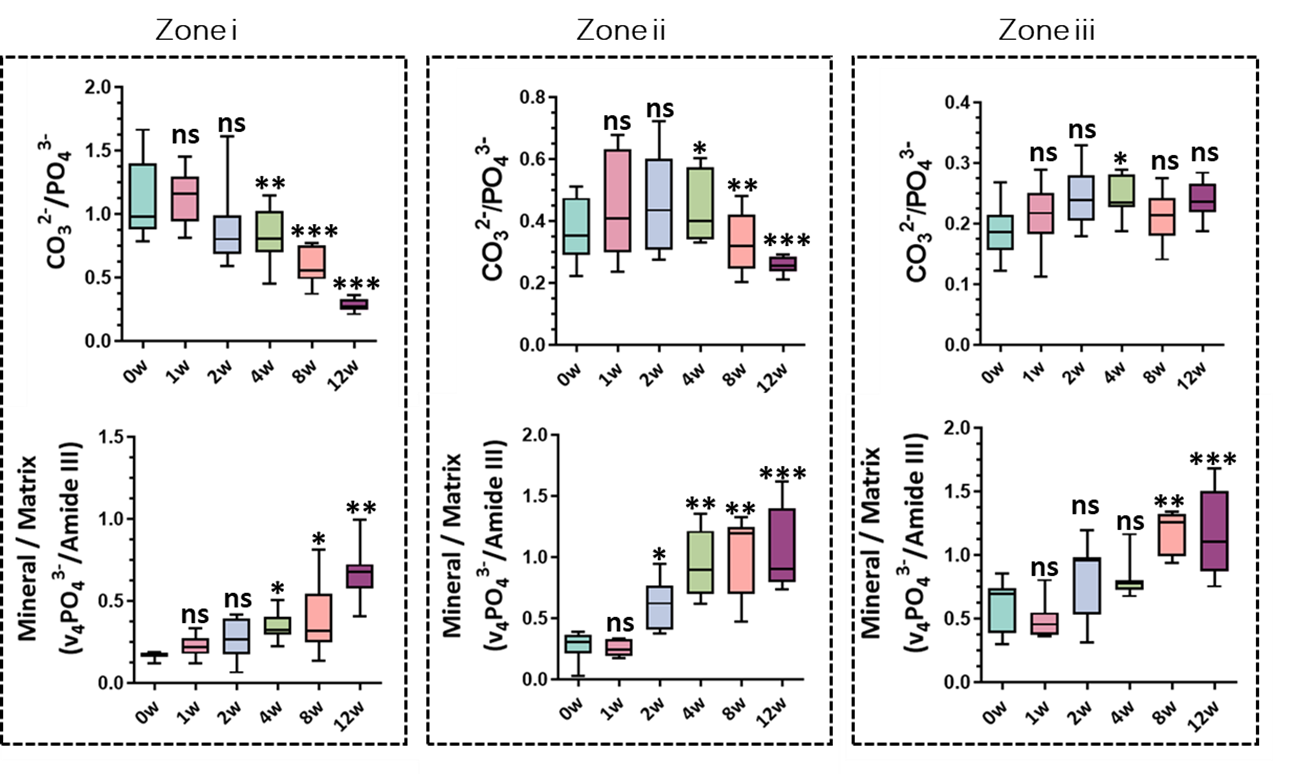


**Fig. S23. Quantitative analysis of CO_3_^2-^/PO_4_^3-^ ratio and mineral/matrix ratio in three area (zone i to zone iii) of the osteochondral interface (n = 3) (Rabbit TMJOA models).** ns, no significance. **P* < 0.05, ***P* < 0.01, and ****P* < 0.001.


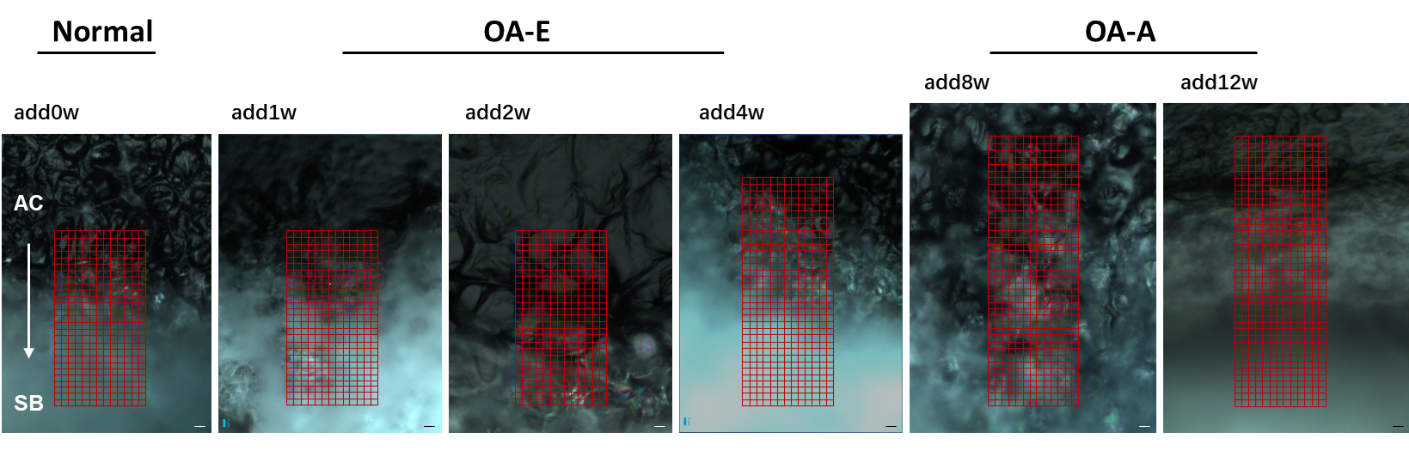


**Fig. S24. Method of micro-Raman mapping analysis in OA cartilage osteochondral interface (Rabbit TMJOA models).** AC, articular cartilage; SB, subchondral bone. Step size, 1 μm.


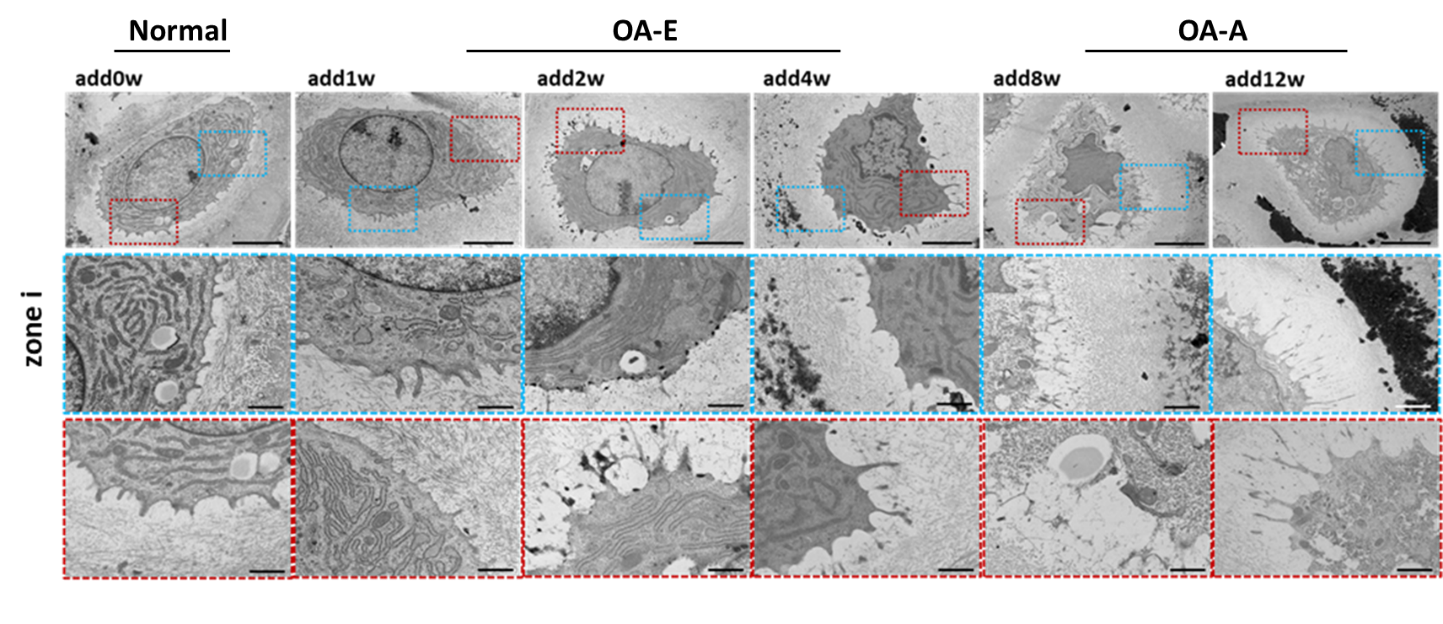


**Fig. S25. Representative TEM images of chondrocytes in front of osteochondral interface during OA development (Rabbit TMJOA models).** Scale bar, low magnification image was 10 μm, high magnification image was 1 μm.


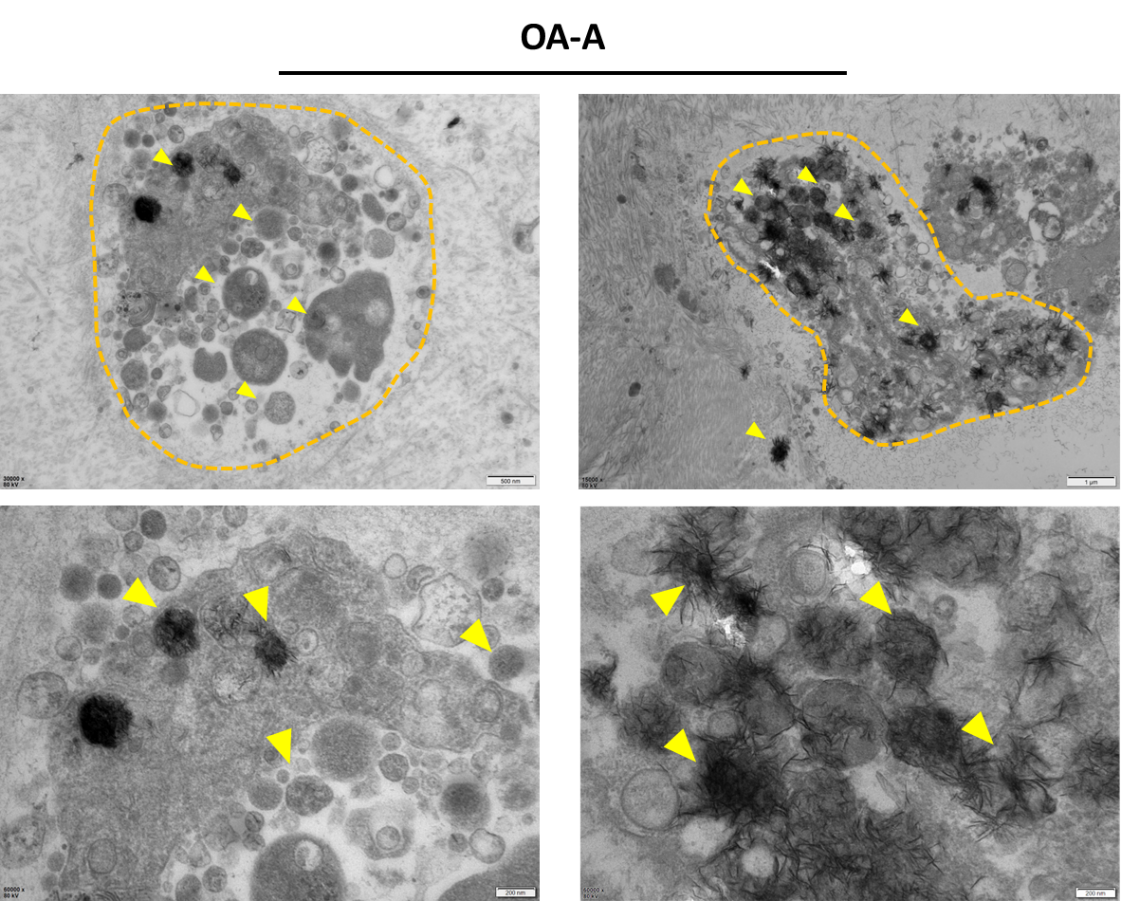


**Fig. S26. Hypermineralized calcified MVs in OA advancement (yellow arrow indicated) (Rabbit TMJOA models).**


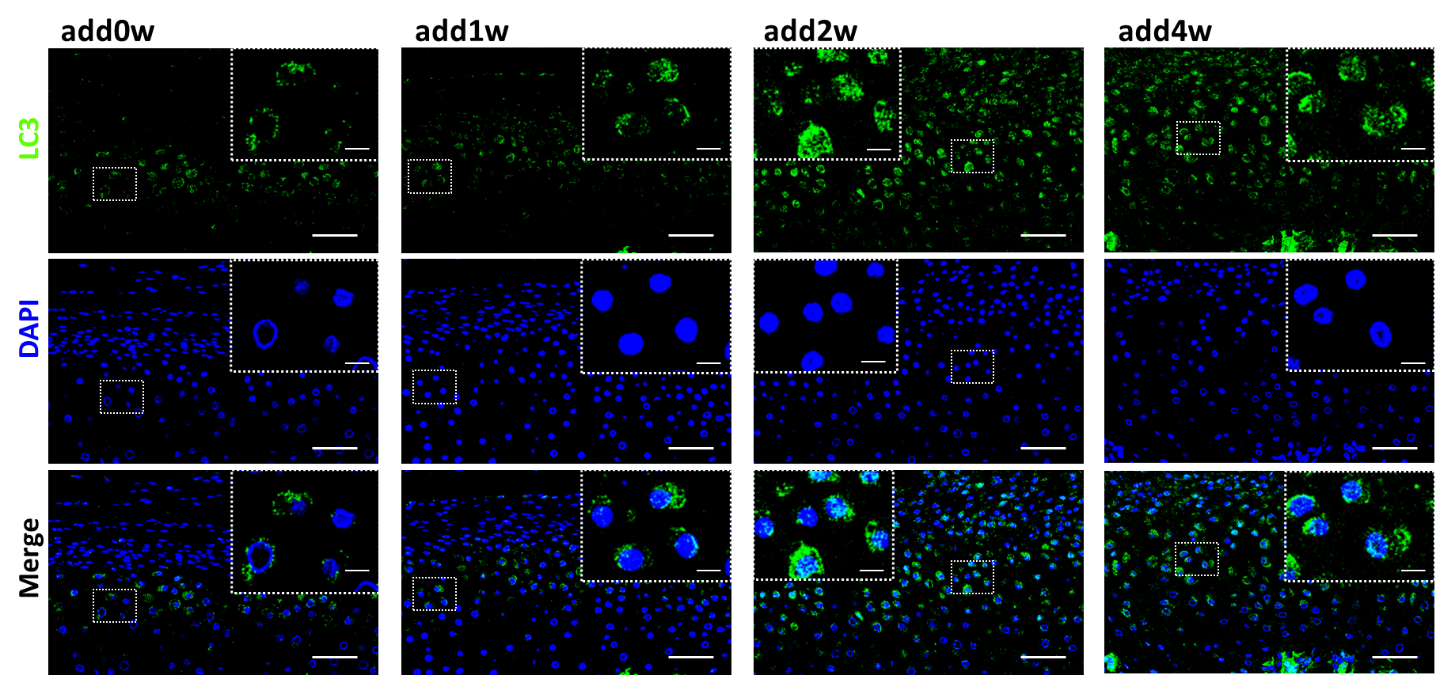


**Fig. S27. Immunofluorescence staining of LC3 represented autophagic matrix vesicles in front of osteochondral interface regions during TMJOA progression (Rabbit TMJOA models).**  Low-magnification scale bar: 250 μm, high-magnification scale bar: 25 μm.


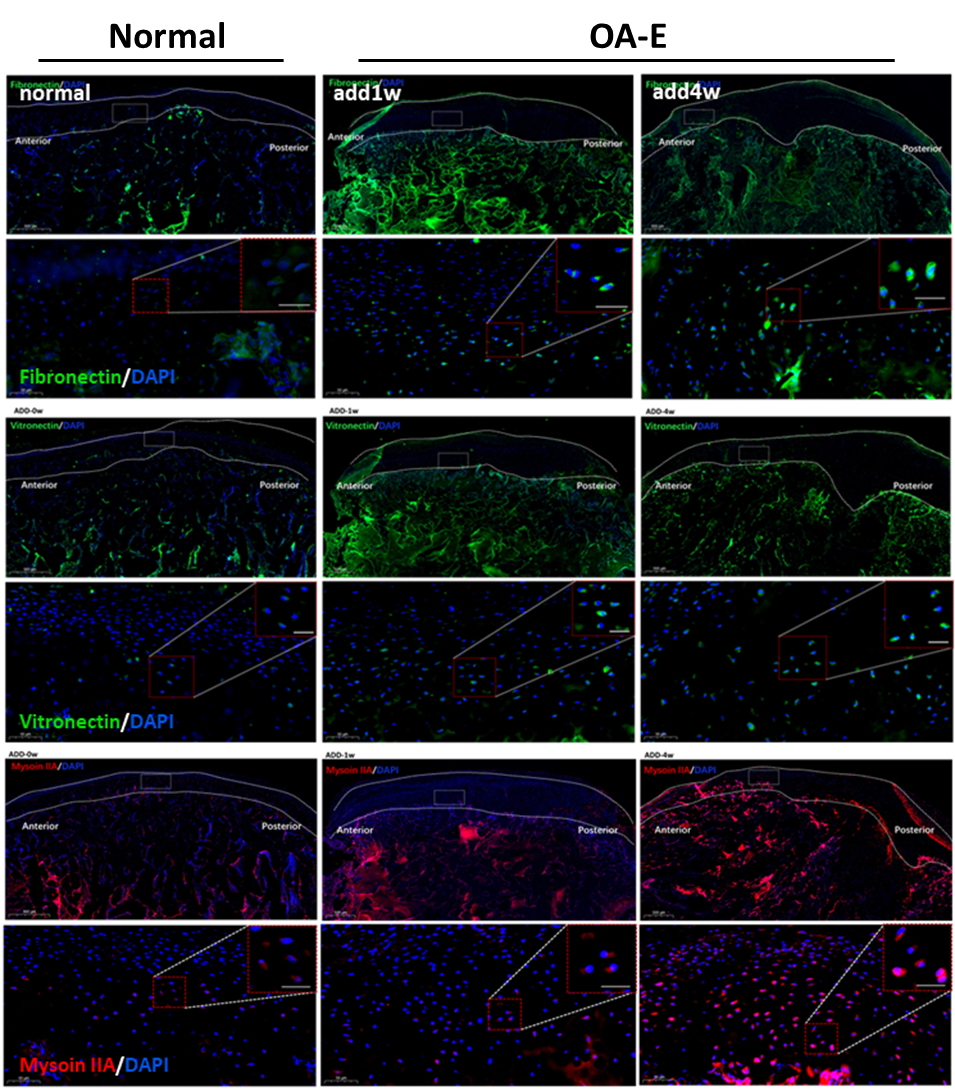


**Fig. S28. Immunofluorescence staining of fibronectin, vitronectin and myosin IIA protein represented changes of mechanical microenvironment in chondrocytes in front of osteochondral interface regions during TMJOA progression** **(Rabbit TMJOA models).** Fibronectin and vitronectin, green; Myosin IIA, red; DAPI, blue. Low magnification image scale bar: 500 μm; High magnification image scale bar: 50μm.

**
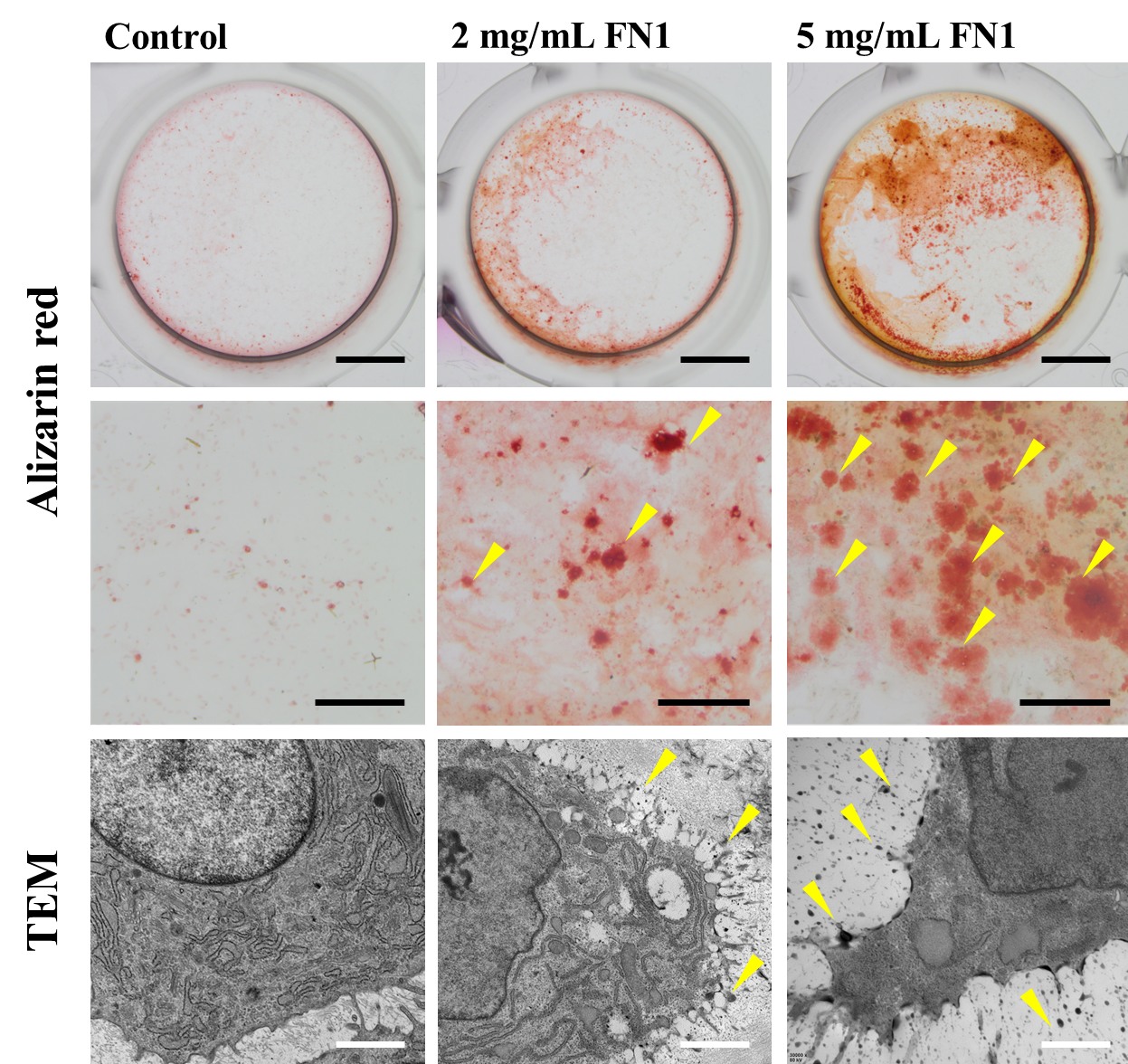
**

**Figure S29. Fibronectin was co-cultured with chondrocytes to induce the secretion of calcified matrix vesicles.** Alizarin Red staining and TEM were used to observe the morphology of calcified vesicles secreted both inside and around the chondrocytes. Scale bar, upper line 10mm; middle line 50 μm; deep line 2μm.


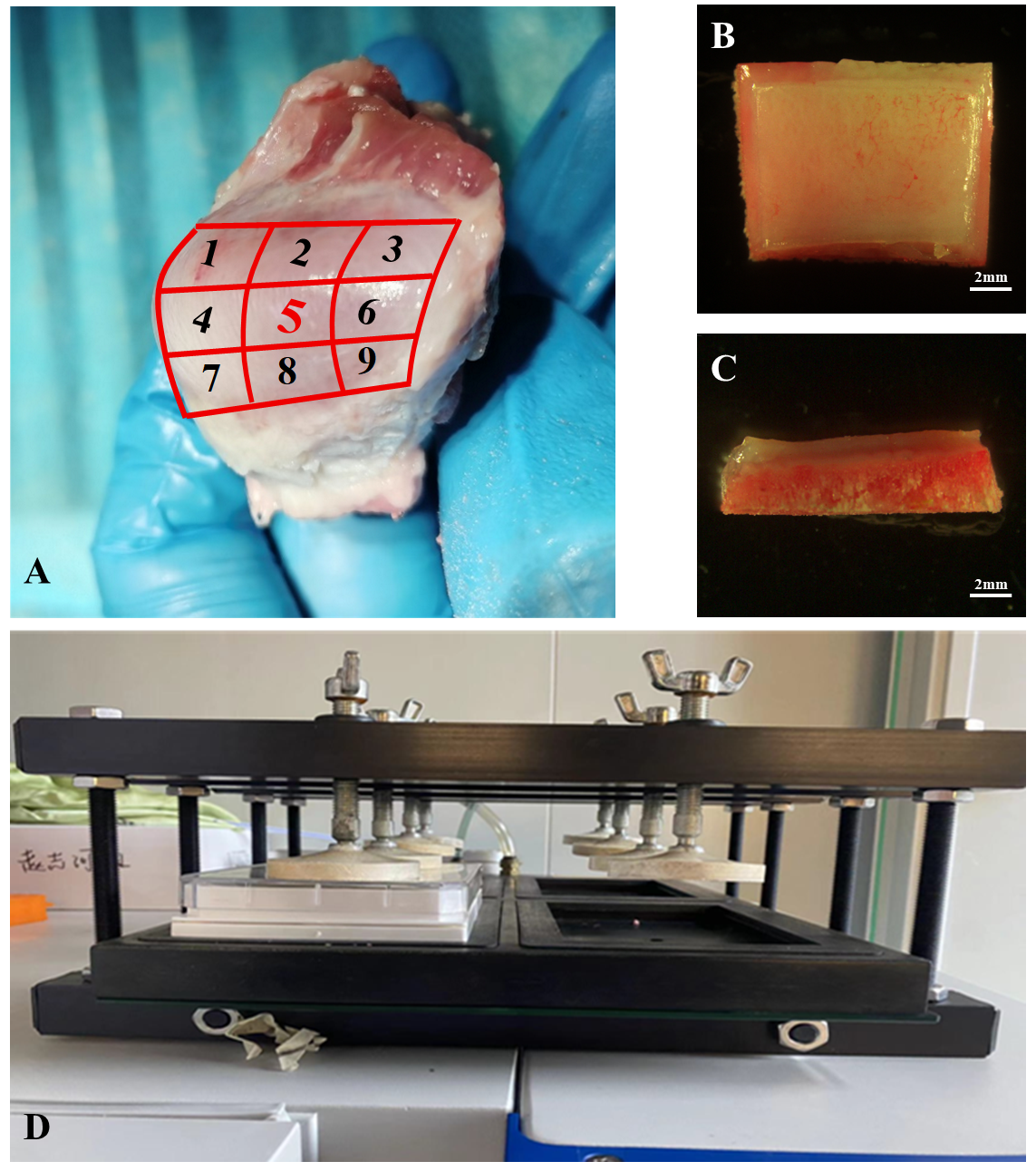


**Fig. S30. Invitro cartilage plugs loading culture method performed by Flexcell-5000^TM^ Compression System (Porcine TMJ condylar cartilage plugs). A. Standardized sampling locations in condylar cartilage: The superior surface of the condylar cartilage was equally divided into 9 sections, with the central section 5 being the cartilage block selected for the experiment; B. Top view of a 1cm square cartilage block; C. Cross-sectional view of the cartilage block; D. Flexcell-5000^TM^ Compression System.**


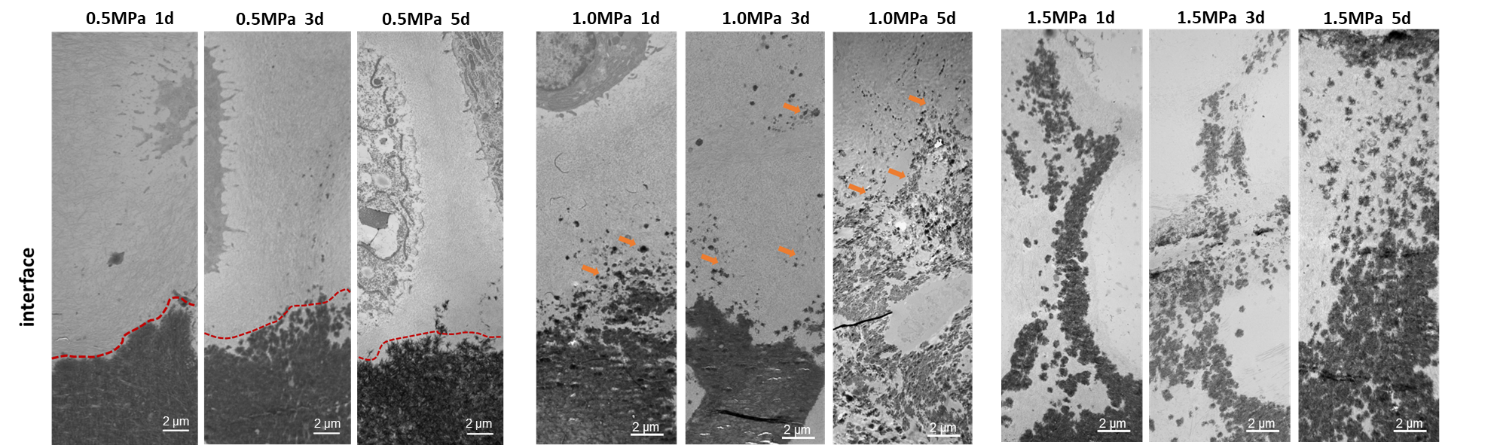


**Fig. S31. TEM images of osteochondral interface in cartilage plugs in invitro loading culture system from 0.5 MPa to 1.5 MPa and 1 day to 5 days (Porcine TMJ condylar cartilage plugs).** Scale bar 2μm.


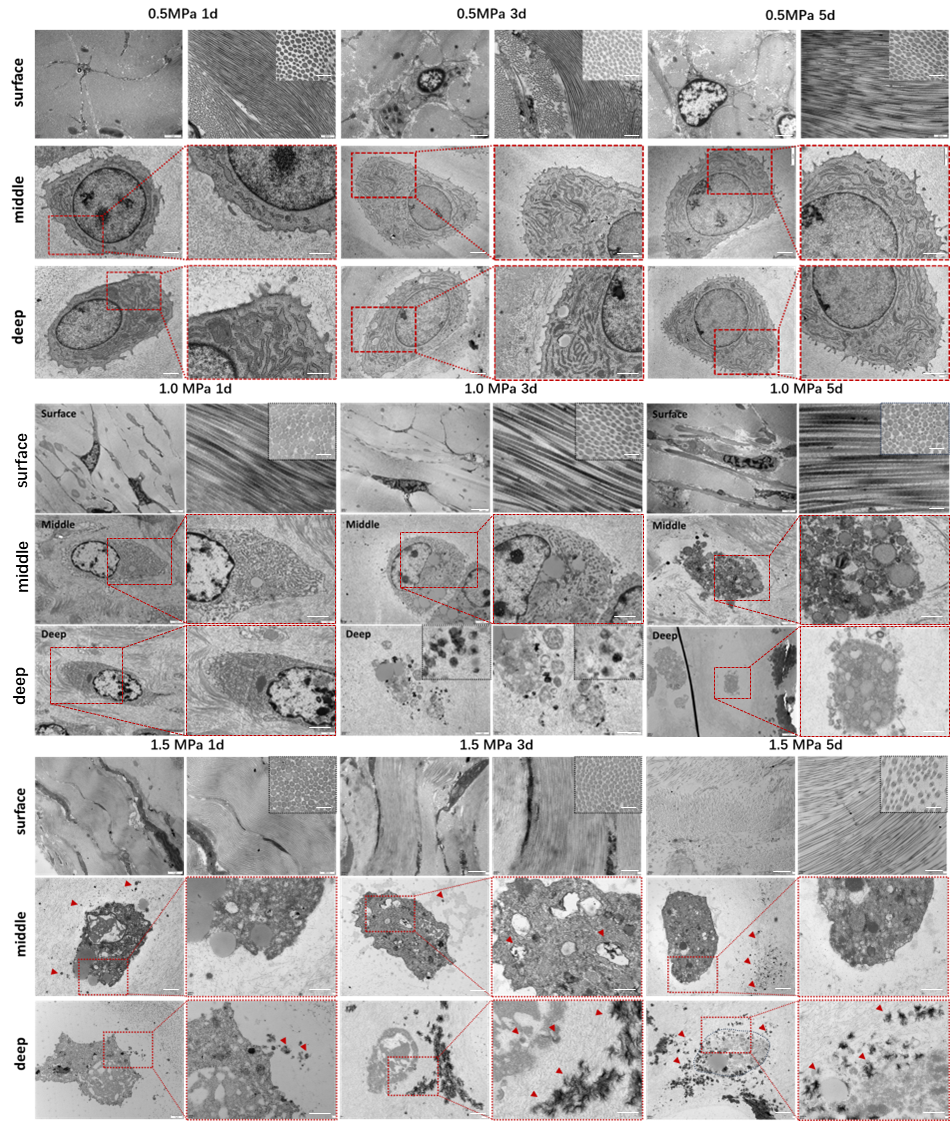


**Fig. S32. TEM images of micro structure of cartilage plugs in invitro loading culture system from 0.5 MPa to 1.5 MPa and 1 day to 5 days (Porcine TMJ condylar cartilage plugs).**


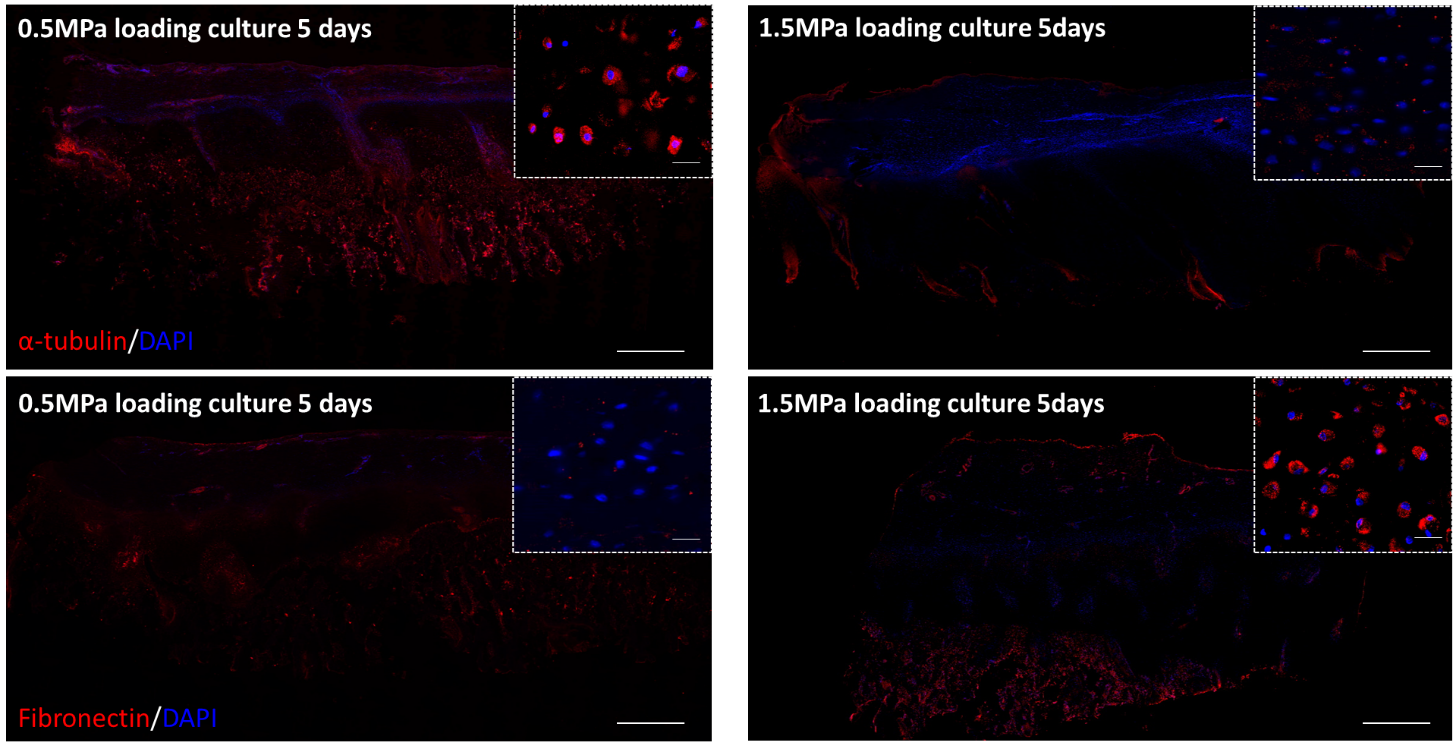


**Fig. S33. Immunofluorescence staining of fibronectin and α-tubulin protein in cartilage plugs in invitro loading culture system from 0.5 MPa to 1.5 MPa for 5 days (Porcine TMJ condylar cartilage plugs).** Fibronectin, α-tubulin, red; DAPI, blue. Low magnification image scale bar: 500 μm; High magnification image scale bar: 50μm.


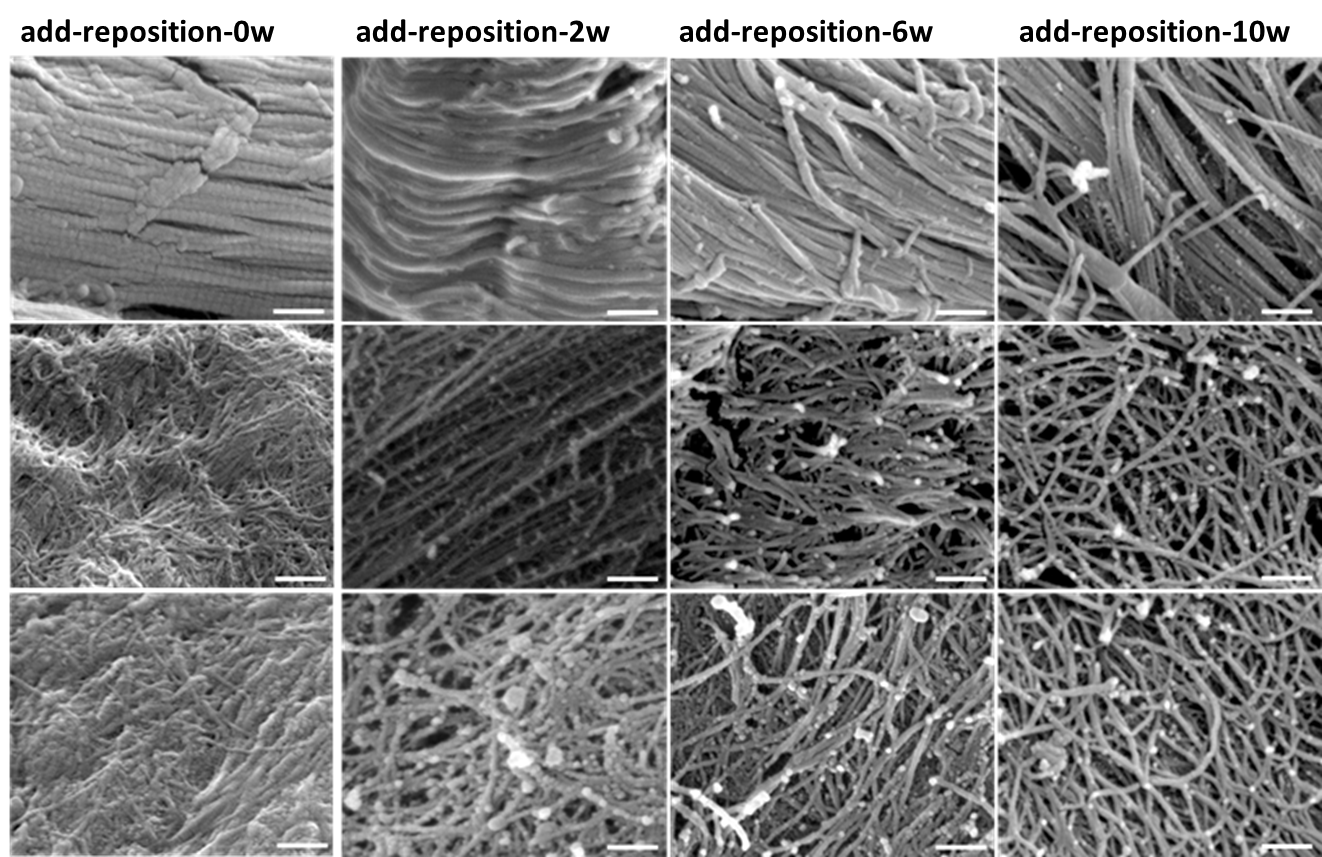


**Fig. S34. SEM images of micro structure of cartilage in ADD repositioned models (Rabbit TMJOA models).** Scale bar, 200nm.


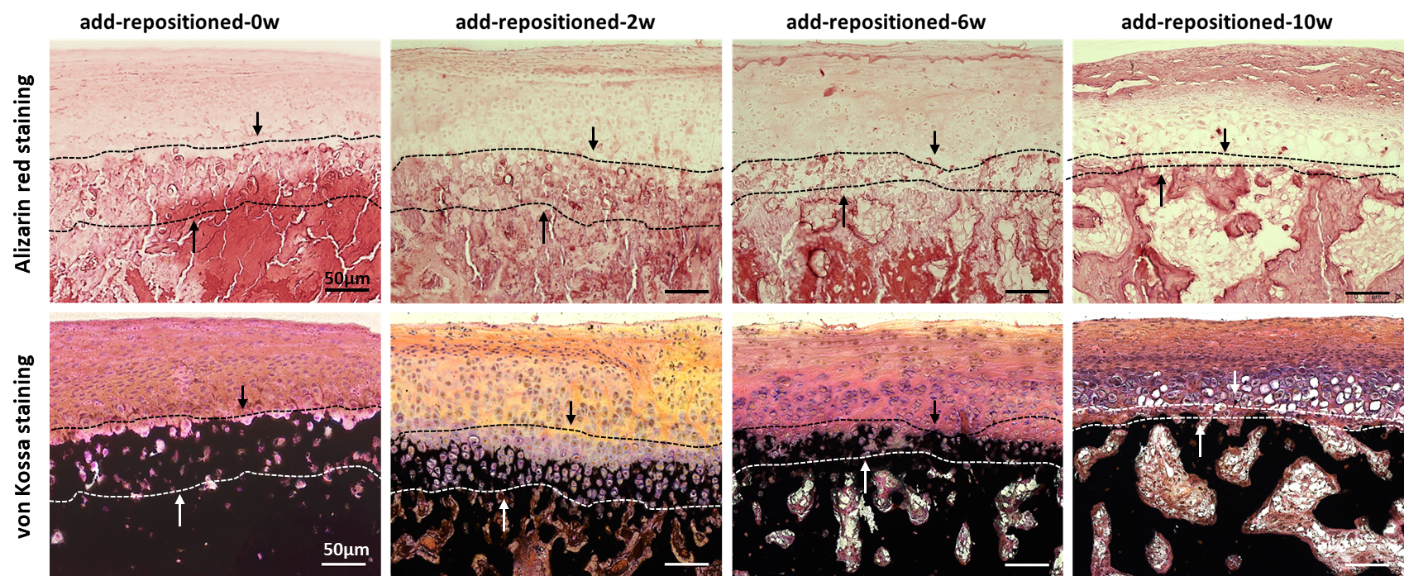


**Fig. S35. Von Kossa and Alizarin Red S staining and calcium distribution in osteochondral interface of ADD repositioned group during OA reversion development (Rabbit TMJOA models).** Scale bar 50 μm.


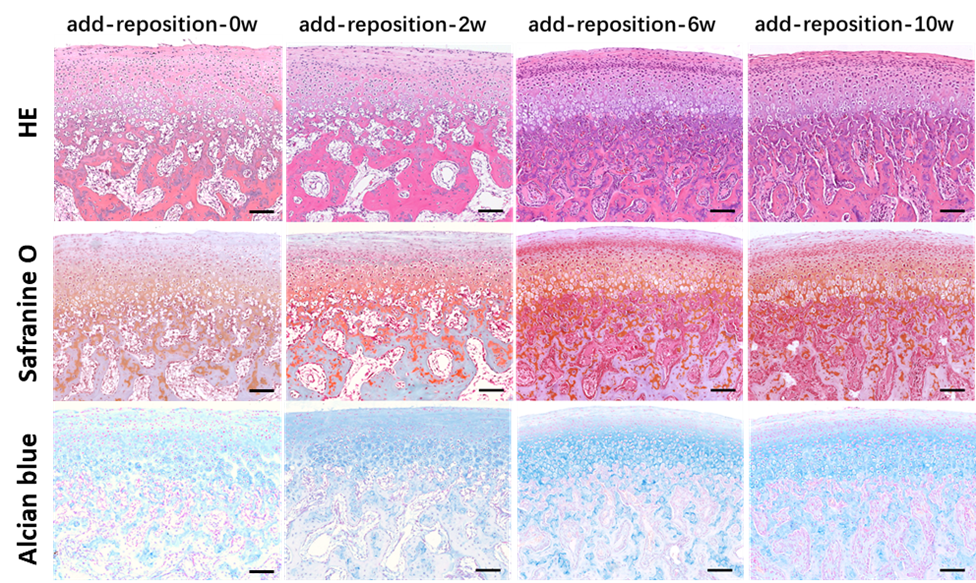


**Fig. S36. Histological features of condylar cartilage in ADD repositioned group during OA reversion development (Rabbit TMJOA models).** Scale bar, 50 μm.


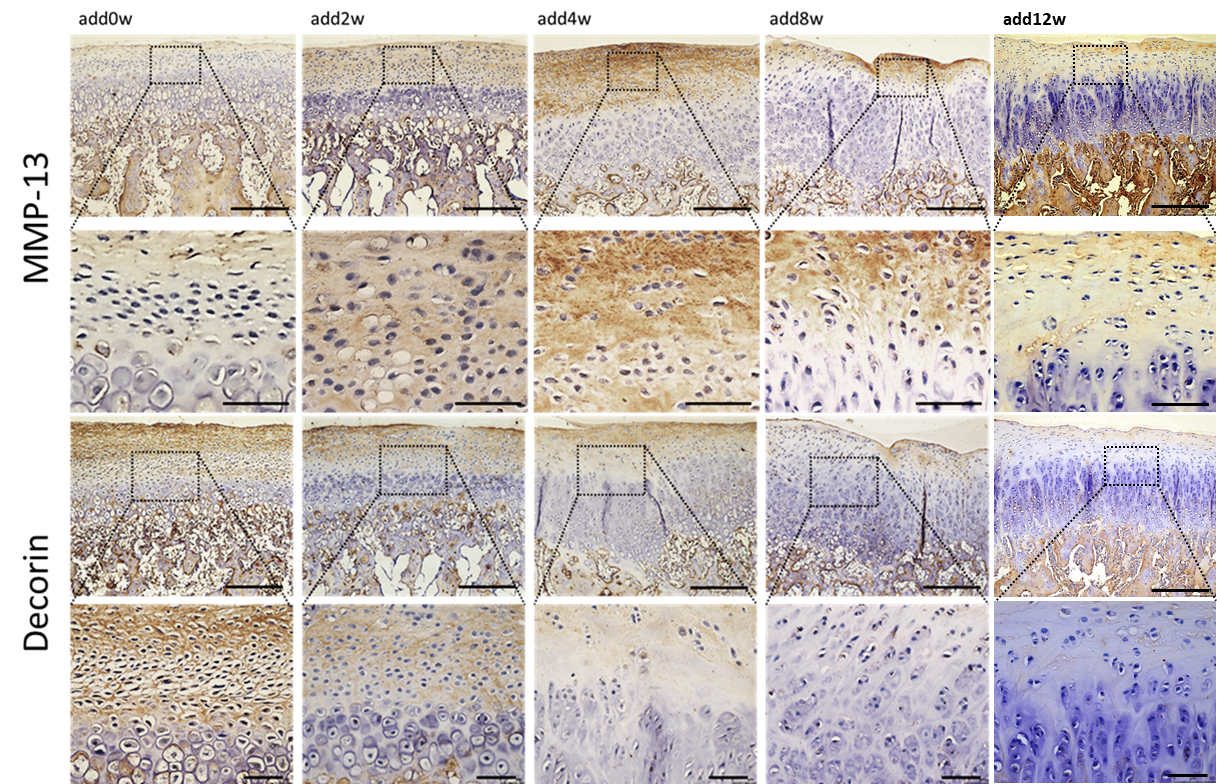


**Fig. S37. Immunohistochemical staining of MMP-13 and decorin mediating matrix degradation and fibril orientation in surface and middle layer during OA progression, respectively (Rabbit TMJOA models).** Low magnification image scale bar: 250 μm; High magnification image scale bar: 50μm.


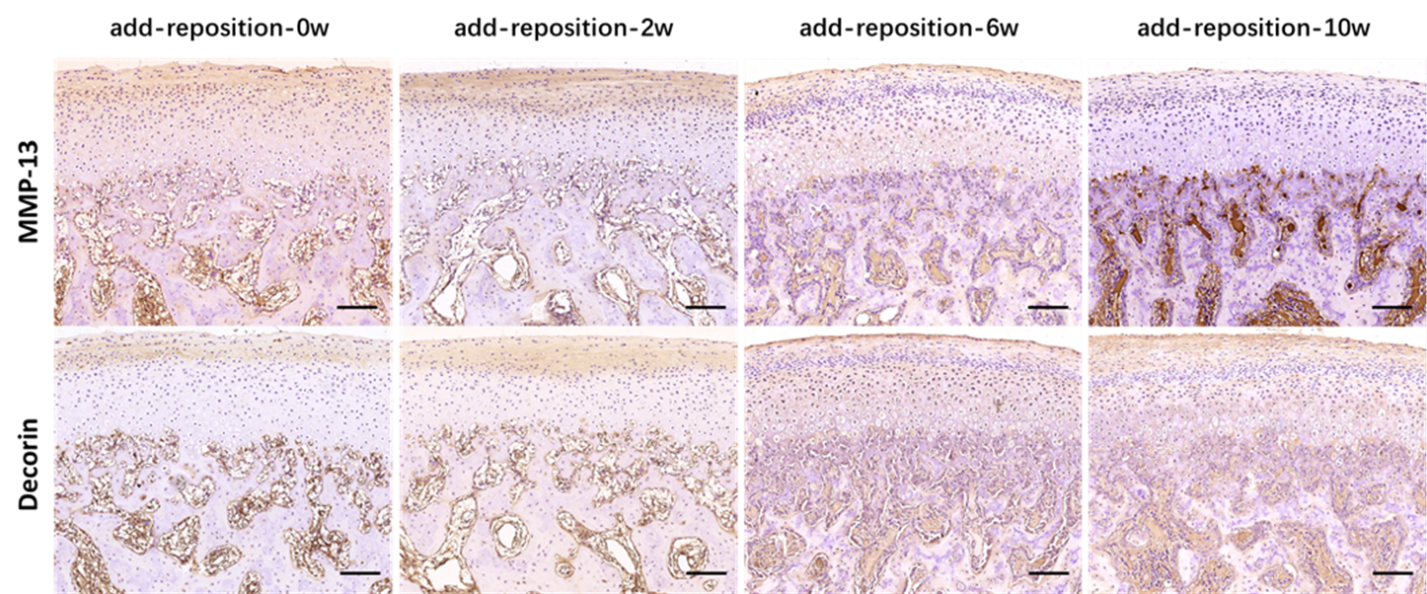


**Fig. S38** **Immunohistochemical staining of MMP-13 and decorin in ADD repositioned group during OA reversion development (Rabbit TMJOA models).** Scale bar, 50 μm.

**Table. S1. OA cartilage samples from five species of this study**

| **Sample**  **species** | **Experiment**  **section** | **Replicates Number** | **Ages** |
| --- | --- | --- | --- |
| **Human** | **Section1** | **N=3 /time point** | **34.2±6.8 yrs** |
| **Porcine** | **Section1** | **N=5 /time point** | **7-8 months** |
| **Rabbit** | **Section1-6,8** | **N=3 /time point** | **6 months** |
| **Rat** | **Section1** | **N=5/time point** | **8 weeks** |
| **Mouse** | **Section1** | **N=5 /time point** | **6 weeks** |
